# Meta-Analysis of Repository Data: Impact of Data Regularization on NIMH Schizophrenia Linkage Results

**DOI:** 10.1371/journal.pone.0084696

**Published:** 2014-01-14

**Authors:** Kimberly A. Walters, Yungui Huang, Marco Azaro, Kathleen Tobin, Thomas Lehner, Linda M. Brzustowicz, Veronica J. Vieland

**Affiliations:** 1 Battelle Center for Mathematical Medicine, The Research Institute at Nationwide Children's Hospital, Columbus, Ohio, United States of America; 2 Department of Genetics, Rutgers University, Piscataway, New Jersey, United States of America; 3 Genomics Research Branch, The National Institute of Mental Health, Bethesda, Maryland, United States of America; University of Turin, Italy

## Abstract

Human geneticists are increasingly turning to study designs based on very large sample sizes to overcome difficulties in studying complex disorders. This in turn almost always requires multi-site data collection and processing of data through centralized repositories. While such repositories offer many advantages, including the ability to return to previously collected data to apply new analytic techniques, they also have some limitations. To illustrate, we reviewed data from seven older schizophrenia studies available from the NIMH-funded Center for Collaborative Genomic Studies on Mental Disorders, also known as the Human Genetics Initiative (HGI), and assessed the impact of data cleaning and regularization on linkage analyses. Extensive data regularization protocols were developed and applied to both genotypic and phenotypic data. Genome-wide nonparametric linkage (NPL) statistics were computed for each study, over various stages of data processing. To assess the impact of data processing on aggregate results, Genome-Scan Meta-Analysis (GSMA) was performed. Examples of increased, reduced and shifted linkage peaks were found when comparing linkage results based on original HGI data to results using post-processed data within the same set of pedigrees. Interestingly, reducing the number of affected individuals tended to increase rather than decrease linkage peaks. But most importantly, while the effects of data regularization within individual data sets were small, GSMA applied to the data in aggregate yielded a substantially different picture after data regularization. These results have implications for analyses based on other types of data (e.g., case-control GWAS or sequencing data) as well as data obtained from other repositories.

## Introduction

For the past two decades, NIMH-funded investigators conducting genetic research have been strongly encouraged to contribute biospecimens, along with whatever corresponding genotypic and phenotypic information they have assembled, to a centralized repository housed at the Center for Collaborative Genomic Studies on Mental Disorders at Rutgers University and Washington University. The repository grows immortalized cell lines, supplies DNA to researchers, and also provides downloadable copies of clinical and genotypic data files through the Human Genetics Initiative (HGI, nimhgenetics.org). The HGI is an enormous resource for the psychiatric genetics community, particularly insofar as it facilitates joint analysis of multiple studies, allowing analyses based on far larger sample sizes than can be accomplished by any one research project. (Note that the HGI has recently been renamed the NIMH Repository and Genomics Resource.)

At the same time, large-scale data repositories such as the HGI present challenges that must be approached with care. Differences due to study design, ascertainment strategies, and evolving standards in psychiatric genetic practice make it challenging to standardize data across contributing studies for purposes of combined analysis. Additionally, while errors in their own data are likely discovered by researchers before publication, there are no formal mechanisms to ensure corresponding corrections in repository files after the initial data deposit. Researchers may also have access to additional clinical information not conveyed by the repository files, perhaps because they were never recorded in standardized digital form in the first place, not collected under the same funding mechanism or time frame as the primary data, or not requested by the repository at the time of deposition. Data regularization efforts at the repository itself may also introduce discrepancies, for example when annotations describing non-standard use of diagnostic codes by a specific study are not prominently featured in data summaries. As a result, data found in repositories tend to differ from data as published by the primary investigators. Indeed, it is frequently not possible to completely reconcile simple quantities of data (number of cases or families) between HGI files and published reports describing the same data sets.

We report here results from the Combined Analysis of Psychiatric Studies (CAPS), a collaborative project with the HGI, a primary aim of which is to review and regularize HGI data across multiple studies, returning to the community sets of data specifically configured for cross-study analysis. In this paper, we describe the methods used to regularize the data in application to all available HGI multiplex family data for schizophrenia, and show the impact both on the data themselves and on linkage analysis results. One advantage of working with these older data is that we can handle them now with the benefits of hindsight. We expect lessons learned from this exercise to apply to future uses of very different types of data and multiple data repositories. All protocols and data files used here are available to the research community at https://www.nimhgenetics.org/projects/CAPS.

## Methods

In this section we (i) give an overview of available *Studies and Subjects* and (ii) describe *Preliminary Data Handling*, used to prepare files for subsequent processing steps. We distinguish two separate versions each of the genotypic and phenotypic data. The first version (HGI-Geno and HGI-Pheno) contains the data as downloaded from the HGI with minimal manipulations (as described below); this is the version that most end-users of the repository would presumably rely on for their own analyses. The second version (CAPS-Geno and CAPS-Pheno) represents the data after processing under our regularization protocols. Details regarding the four corresponding data sets are given in subsections (iii)–(vi) respectively. Finally, we (vii) describe *Statistical Methods* used in assessing the impact of data regularization on linkage results.

### (i) Studies and Subjects

We started with all available data as of April 2011, at which point the HGI had data on seven schizophrenia studies with multiplex families and sufficient genotypic data to perform genome-wide linkage analysis. For brevity, we refer to the 7 studies as Study 1-Study 7. Table 1 provides summary information on each study, including HGI sample sizes and corresponding sample sizes from the original publications reporting on these data. In aggregate the HGI files data contain 1,552 pedigrees, with phenotypes available for 6124 individuals (average 3.9 per pedigree), and genotypes available for 6,512 individuals (average 4.2 per pedigree). Note that this excludes 100 pedigree IDs found in either the HGI “distribution” (phenotype) or genotype file that were dropped prior to data processing (including effective trios, families duplicated between Study 5 and Study 6, families not found in the distribution file, and a small number of unresolvable IDs across the two sets of HGI files).

**Table 1:** Overview of Included Studies and Sample Sizes.

Study	NIMH study	NIMH data	Ancestry^1^	Reference families	Download families^2^	Processed families^3^	Analysis families^4^	Reference
Study 1	0	1	African American	30	28	28	19	[12]
Study 1	0	1	European American	43	46	45	21	[13]
Study 2	6	11	African American	146	146	146	86	[14]
Study 2	6	11	European American	263	266	266	166	[14]
Study 3	3a	12	Han Chinese	606	574	571	497	[15]
Study 4	15	21	European American	43	40	40	17	[9]
Study 5	13	22	Hispanic	99	95	94	69	[16]
Study 6	13	23	Hispanic	175	185	175	101	[17]
Study 7	22	24	African American	217^5^	172	169	44	[18]
Total				1622	1552	1534	1020	

^1^ Total sample sizes by Population Group are N = 346 African American, N = 352 European American, N = 574 Han Chinese and N = 280 Hispanic.

^2^ This count omits 100 pedigree IDs dropped prior to processing, primarily due to uninformativeness for linkage or duplication across Studies 6, 7.

^3^ 15 families were dropped (and 3 subsumed by joining) prior to this stage, see Appendix S1 & Table S1 for details.

^4^ Families used in this paper to compare results across the four data configurations are those remaining after genotype processing with at least two schizophrenia cases according to either the HGI or CAPS clinical criteria, omitting 16 such families with bitsize larger than 24 for computational reasons.

^5^ Study 7 included trios in the published total.

Because studies differ in both genotyping and phenotyping procedures, we process the HGI data separately by study. We further subset by major population groups, based on study-reported group affiliation by family: African American, European American, Han Chinese, or Hispanic. Because many studies included only a single group by design, in all this left 9 distinct subsets for data processing purposes, as indicated in Table 1. [Note that the original studies either focused ascertainment on a single group, or published their results stratified by group. Following their subgrouping therefore maintains comparability with the original analyses. Note too that the issue of ancestry per se is not critical in linkage analysis, by contrast with association analysis. Here it comes into play primarily with respect to construction of genetic maps (see below).]

### (ii) Preliminary Data Handling

Prior to beginning data processing, we harmonized the multiple sources of pedigree information as well as the marker sets across studies. We coordinated closely with HGI staff, as well as with the original investigators in some cases, in order to resolve discrepancies between the HGI distribution file (which contains phenotypes) and the files containing genotypes, which the HGI maintains separately. For the purposes of combining results across studies, some preprocessing of marker names was required to generate a common reference map, e.g., consolidating alternate aliases and dropping custom suffixes. The reference map was obtained by locating hg19 (Build 37) physical positions for all possible markers using the UCSC genome browser, CIDR or NCBI databases. We discarded markers that could not be found in any of these databases based on hg19. Genetic positions were then obtained from the Rutgers Combined Linkage-Physical Map [1].

### (iii) Preparation of HGI-Geno

Studies 1–6 used microsatellite markers (978 unique markers across studies) with median inter-marker distance 9.0030 cM, median of 10 alleles per marker, and average heterozygosity 76%. Study 7 used a set of 5,713 SNPs spaced every 0.65 cM on average. (Study 4 also included 93 SNPs that we did not use.) The HGI data included genetic positions for all studies and marker allele frequencies for 4 studies; frequencies were not included for Studies 1, 3 and 4. MENDEL [2] was run to remove Mendel errors, by setting to missing the genotypes of all individuals in a family for a marker giving an error. We also used MENDEL to estimate allele frequencies using maximum likelihood for those studies lacking allele frequencies in the data download.

### (iv) Preparation of HGI-Pheno

The HGI clinical data (Schizophrenia Release 8.0) included potentially multiple DSM-IIIR and DSM-IV codes per individual for lifetime diagnoses, plus a single algorithmically assigned overall diagnosis: schizophrenia (designated in the HGI files as “SZ”), schizoaffective (“SA”), schizoaffective depressive type (“SADD”), narrow spectrum (“NSPECT”), broad spectrum (“BSPECT”), never mentally ill (“NMI”), or other (“OTHER”). Working with HGI staff, a new classification of “no psychiatric illness” (“NPI”) was implemented, replacing NMI and distinguishing varieties of OTHER, with uniform rules established to overcome what had been some inconsistencies in application of these categories across the collection. We assumed that most end-users of the HGI data would rely on the overall diagnoses, rather than DSM codes, and for processing purposes we utilized HGI's Model III: the broadest definition in which schizophrenia, schizoaffective, schizoaffective depressive type, narrow spectrum and broad spectrum are coded as “affected.” Individuals coded as NPI (no psychiatric illness) were classified as “unaffected,” and the remaining individuals were classified as “unknown.” Note that the depositing investigators' best estimate final diagnosis (BEFD) was not required by the HGI and was therefore generally not available.

### (v) Preparation of CAPS-Geno

A number of data processing steps were taken to regularize the HGI-Geno data separately for each subset, imposing reference map marker order throughout. PEDSTATS [3] was run to identify and drop markers with Hardy-Weinberg equilibrium (HWE) p-value <0.0001. In-house scripts were used to remove markers missing >10% and zero individuals missing >20% of genotypes. Study 7 was only partially genotyped on the X chromosome, and therefore chromosome X markers were retained in this sample with up to 14% missingness. RELCHECK [4] was then run to verify and adjust pedigree structures. Mendel errors were assessed, including total number of errors per family and per marker, using MENDEL and zeroed-out as described under HGI-Geno above. Checks for Mendel errors, missingness, and HWE were repeated (in this order) in case any new markers or individuals crossed our criteria for removal during the previous steps. At each step, histograms were visually inspected to check that cutoffs were appropriate given the observed distributions (e.g., that they successfully captured outliers). Inspection of the histograms led us to raise the missingness thresholds to 25% for individuals and 15% for markers during the final iteration. (A complete set of histograms can be found in Appendix S1.) Additional checks at this stage included looking for duplicated individuals across pedigrees using RELCHECK and verifying concordance between recorded sex and X-chromosome genotypes. When an individual's sex could not be resolved by genotyped mate and offspring (without RELCHECK problems), the X-Genotypes were zeroed and the phenotype of the individual was set to unknown to protect against undetectable sample swaps.

Finally, we constructed subset-specific maps based on the data as processed to this point, to allow for differing inter-marker genetic distances and marker allele frequencies across studies and, in particular, across population groups. For the microsatellite data, marker-to-marker genetic distances were estimated using KELVIN [5], and marker allele frequencies were estimated by maximum likelihood estimation using MENDEL for all data sets, including those for which frequencies had been included in the HGI files. For the SNP data, marker allele frequencies were similarly estimated, but physical locations were interpolated directly onto the reference map.

### (vi) Preparation of CAPS-Pheno

The primary aim of phenotypic data regularization was to ensure consistency in the handling of clinical complexities across studies, in preparation for multi-study linkage analysis. We therefore worked from the original investigator-supplied DSM codes, rather than the algorithmic HGI diagnoses. Note, however, that these codes represent lifetime diagnoses, without temporal data (although such information may have been available to the original investigators). Therefore, it was not possible for us to distinguish comorbid conditions from conditions that occurred over the course of illness or due to disease progression. Given the data available to us, or to any end-user of the HGI, we opted to take a conservative approach to diagnostic classification, in order to focus on a core phenotype that could be consistently assigned across studies. Additionally, we would expect our conservative approach to increase power to detect linkage. Linkage analysis is particularly sensitive to the miss-classification of unaffected individuals as affected, a type of error that can mimic both inter- and intra-familial heterogeneity, and as a result can severely reduce power. Of course classifying affected individuals as unaffected or unknown can also reduce power through loss of information, but the two types of errors are not equivalent in their impact, depending on the type of analysis employed.

We first applied exclusionary criteria, recoding diagnosed individuals as phenotype “unknown” in the presence of: all dementias, amnestic and cognitive disorders; unknown/unspecified or deferred diagnoses on Axis I; and substance-related disorders that have been linked to schizophrenia or that cause ancillary psychiatric symptoms. Also set to “unknown” were individuals with Major Depressive or Bipolar Disorder coded as either “severe, with psychotic features” or “severity unknown.” (A complete list of corresponding DSM-IIIR and DSM-IV codes is found in Appendix S2, along with our final diagnostic algorithm.) Remaining schizophrenia cases, coded as “affected,” were divided into two levels: (i) Schizophrenia (Disorganized, Catatonic, Paranoid, and Residual types of Schizophrenia Disorder); and (ii) Schizophrenia/Affective (Schizoaffective Disorder or any Schizophrenia Disorder and a significant Affective Disorder). Schizophrenia individuals with comorbid diagnoses of Recurrent Major Depressive or Bipolar Disorder were recoded as Schizophrenia/Affective in order to account for the affective presentation while taking into consideration diagnostic uncertainty due to lack of temporal data. We also classified as Broad Spectrum anyone meeting criteria for a Delusional Disorder, Brief Psychotic Disorder, Psychotic Disorder NOS, Schizophreniform Disorder, or Cluster A Personality Disorder. Because we would expect more variability in the diagnosis of individuals with broad spectrum rather than clear-cut core schizophrenia, Broad Spectrum subjects were recoded to “unknown;” only 187 individuals (<7% of affected individuals) fell in this category. Individuals assigned a diagnosis of Bipolar I or II Disorder NOS, Mood Disorder NOS, Depressive Disorder NOS, Personality Disorder NOS, or Diagnosis Deferred on Axis II, were coded as “unknown.” Finally, individuals with a clinical assessment who did not meet criteria for Schizophrenia, Schizophrenia/Affective, or Broad Spectrum and who also did not have any of the exclusionary diagnoses were coded as “unaffected.”

### (vii) Statistical Methods

In order to assess the impact of the CAPS data processing protocols on linkage analysis results, we computed multipoint nonparametric linkage (NPL) scores based on S-pairs [6] using MERLIN [7]. All but one of the original publications used either the NPL or the closely related z-score of Whittemore and Halpern [8]; the one exception was Study 4 [9], which utilized MCMC and Variance Components. Analyses were conducted separately for each data subset, and in order to distinguish the impact of changes in genotypic or phenotypic data separately, we compared results over the following four configurations: HGI-Pheno+HGI-Geno, HGI-Pheno+CAPS-Geno, CAPS-Pheno+HGI-Geno, and CAPS-Pheno+CAPS-Geno.

To assess the aggregate impact of data processing across the 9 data subsets, we conducted Genome Scan Meta-analysis (GSMA) [10] based on the NPL results. Following recommendations in [10] and [11], empirical p-values (one million replicates) were based on the observed square root weighted summed ranks of bin-maximum NPLs, using bin sizes of 25 cM. In order to align genetic positions across studies, we first projected the subset-specific NPL results onto the common reference map described above.

In order to conduct “apples to apples” comparisons, we included families that were retained through all processing steps in CAPS-Geno, but dropped 16 families too large for MERLIN to handle without trimming either before or after processing. We also included only families with at least two affected cases under both HGI-Pheno and CAPS-Pheno. This ensured identical sample sizes across all four data configurations, allowing us to assess data processing effects independently of confounding by changes in which families were included at various processing stages. (The one exception occurred in Study 3: 6 families listed with separate family IDs in HGI-Geno were joined pairwise in CAPS-Geno, for a net reduction of 3 families). Thus any impact of data regularization on results, as reported below, is due solely to changes made in this set of pedigrees, and not due to differences in sample sizes that would result if we were to base inclusion criteria on CAPS-Pheno rather than HGI-Pheno. Note that as a result, of the 1,534 original families (Table 1), only 1,020 are actually used for purposes of these comparisons. Table S1 (available online) gives HGI ID's for families used at each stage and annotates reasons for dropping families where applicable.

## Results

In what follows, we (i) detail the effects of data processing on the data themselves; (ii) illustrate the separate and combined effects of phenotype and genotype processing on linkage results in individual data subsets; and (iii) examine the overall effect of processing on meta-analyses of the entire HGI data set.

### (i) Results of CAPS Data Regularization

During preliminary data handling, we resolved 1,052 discrepancies in family membership between the distribution files and the genotype files (individuals found only in one file or the other), 121 discrepancies in assigned parentage across the two sets of files, and 8 discrepancies in recorded sex. Duplicative marker aliases were resolved for 69 markers. We dropped 5 markers not found in any map database as well as a single custom marker that was redundant with a standard one. There were 14 changes in marker order going from HGI-Geno to CAPS-Geno.

Processing of the genotype data for the initial 1,552 pedigrees resulted in the following actions. Seven markers were dropped due to HWE violations; 241 microsatellite markers were removed in all (with a range of 3 to 81 markers per data set; this left a range of 373 to 395 markers per data set), and genotypes for 46 individuals were zeroed out due to excess missingness. As a result of relationship checking, 5 families were removed and 36 corrections were made to the pedigree structures, including removal of 14 monozygotic twins and 13 other family members. In addition, 10 families were dropped due to excess Mendel errors, and 3 extended pedigrees were reconstituted from 6 separate family IDs (as noted above). There were 52 discrepancies between recorded sex and X-chromosome genotypes, of which only 4 cases were verifiable by genotyped mate and offspring. Estimation of the marker positions and allele frequencies resulted in numerous adjustments from HGI-Geno. Following these steps, there were a total of 1,534 pedigrees.

**Figure 1:**
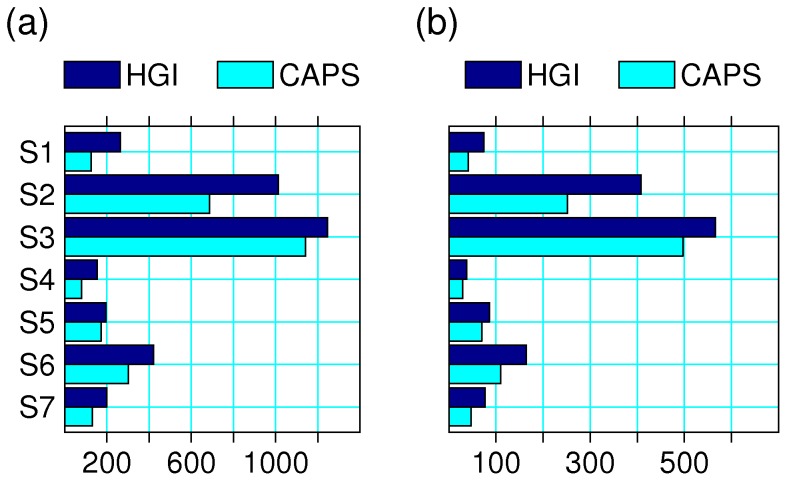
Effects of data processing on (a) number of affected individuals^1^ and (b) number of multiplex families^2^. S1 through S7 indicate study numbers. The bars represent Human Genetics Initiative (HGI) and Combined Analysis of Psychiatric Studies (CAPS) data. ^1^HGI diagnosis includes SZ, SA, SADD, NSPECT and BSPECT; CAPS diagnosis includes Schizophrenia and Schizophrenia/Affective as defined in the text. ^2^HGI includes all 1,413 families with at least two affected individuals by HGI criteria; CAPS includes all 1,046 families with at least two affected individuals by CAPS criteria. Note that analyses presented in the main text utilized the subset of pedigrees satisfying both criteria.

Processing of the clinical data resulted in a considerable number of additional changes. While only 3 out of 5,596 (<0.1%) individuals became affected, 858 out of 3,632 (24%) cases as classified by the HGI became unknown in CAPS-Pheno. Our protocol improved the resolution of the HGI diagnosis OTHER, converting 376 out of 1,036 (36%) to a known phenotype. 1,413 families qualified as multiplex according to a minimal requirement of two broad HGI-Pheno cases, while 1,046 qualified as multiplex in CAPS-Pheno. The impact of data processing on numbers of affected individuals and multiplex families can be seen in Figure 1. Within the restricted set of pedigrees used to assess the impact of data processing on results (below), 224 out of 2,515 (9%) individuals coded as HGI-affected were recoded as CAPS-unknown; 135 out of 1,548 (9%) coded as HGI-unknown were recoded as CAPS-unaffected; and 1 out of 1,565 (<0.1%) coded as HGI-unaffected was coded as CAPS affected.

### (ii) Linkage Results by Data Configuration within Subsets

**Figure 2:**
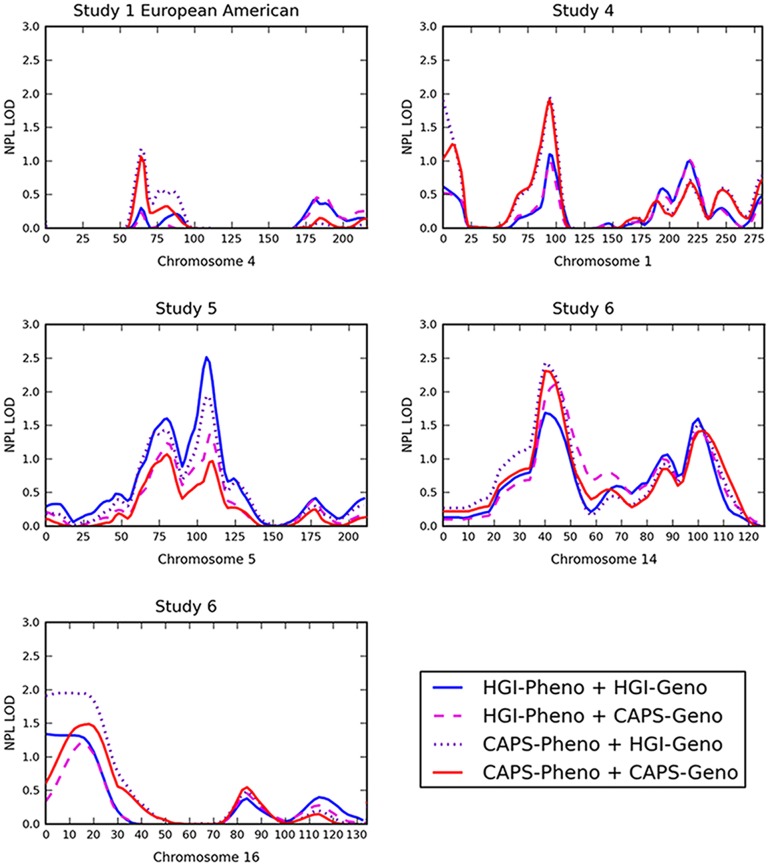
Examples of the effects of data processing on linkage results within individual data subsets. The labels for each line indicate state of phenotype (Pheno) and genotype (Geno) data, which can be Human Genetics Initiative (HGI) or Combined Analysis of Psychiatric Studies (CAPS).

Various effect types can be found across the 9 data subsets and across the genome, including linkage signals that diminish, increase or shift in position following data processing. In general, these changes were small when viewed one data set at a time (but see below). Illustrative examples are shown in Figure 2; complete results are given in Appendix S3. In most cases, phenotype processing had the greater effect. Note, however, that in the majority of cases, the result was an increase in the linkage peak going from HGI-Pheno to CAPS-Pheno, consistent with the CAPS diagnostic criteria reducing heterogeneity within pedigrees. There are also locations at which genotype processing alone affects results, and others where genotype and phenotype processing interact to produce distinct profiles in all four configurations.

### (iii) Effect of Data Processing on Linkage Results in Aggregate

**Figure 3:**
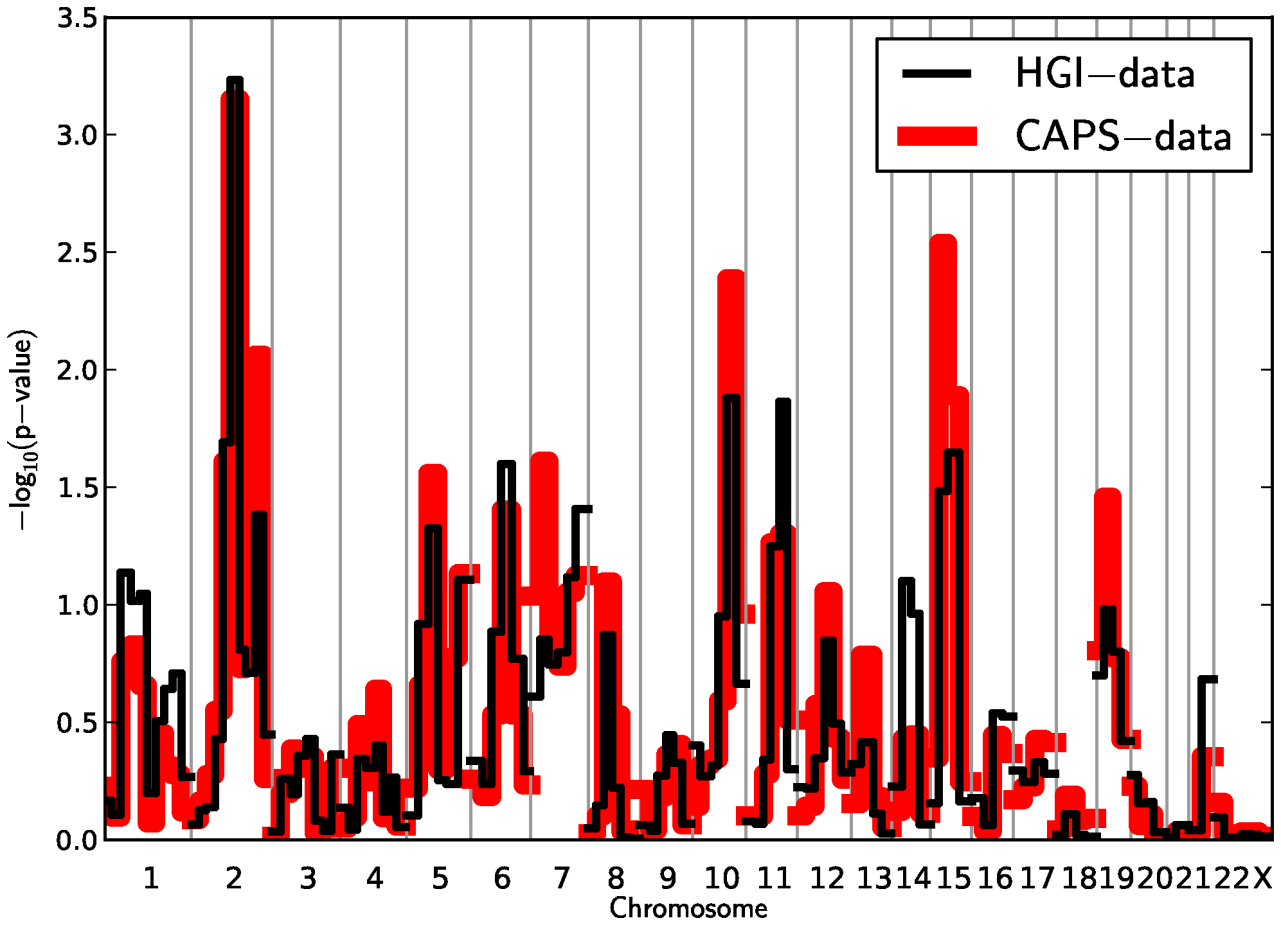
Effects of data processing on linkage results based on meta-analysis. The lines refer to Human Genetics Initiative (HGI) or Combined Analysis of Psychiatric Studies (CAPS) data.

Figure 3 shows GSMA results for all 9 subsets in aggregate, comparing HGI-Geno+HGI-Pheno (HGI-data) with CAPS-Geno+CAPS-Pheno (CAPS-data). As can be seen, there are substantial differences in the overall landscape between the two versions of the data. Of the 5 peaks >1.5 based on HGI-data, two remain close in magnitude (on chromosomes 2, 6), one is substantially reduced (chromosome 11), while two are substantially increased in the CAPS-data (chromosomes 10, 15). Additionally, the CAPS-data yield three new peaks >1.5 (chromosomes 2, 5, 7). It is noteworthy that the accumulation of what appear to be quite small changes in individual data sets results in more substantial aggregate effects on the meta-analytic results, both in terms of the p-values themselves and also the rank ordering of peaks by p-value across the genome.

## Discussion

We have undertaken a thorough and rigorous review of a substantial quantity of data accessible to the research community through the HGI. While the potential utility of such large-scale repositories is obvious, our results clearly demonstrate issues that should be addressed when handling the data. There are numerous opportunities for errors to occur throughout the process of data accumulation and transfer to a repository, and few mechanisms in place for systematic data correction over time, as individual investigators and their collaborators continue to work with the data. Additionally, some clinical information available to the original investigators may never be submitted. Indeed, it is seldom possible to align the publicly available data with published results from the contributing investigators with any specificity. This typically reflects historical and logistical issues with data deposition into repositories, rather than any particular fault on the part of researchers who undertake to make their data available to others. Individual studies may lack funding to implement sophisticated databases, and final data correction steps frequently happen after the end of a grant when personnel may no longer be available to coordinate changes subsequent to initial submission. Despite the invaluable and diligent efforts of original investigators, data from public repositories need to be handled with care.

In this paper we have not attempted to draw any genetic conclusions from the analyses. Rather, our purpose here was to highlight issues of importance when working with repository data, to present the protocols we have used to address these issues, and to illustrate potential impact of data regularization on linkage results. It is noteworthy, however, that an investigator accessing and analyzing the (corresponding subset of) HGI pedigrees for a meta-analysis of schizophrenia linkage results would draw different conclusions than those found in the same families after CAPS processing, with four “suggestive” peaks (−log_10_[p-value] >2) based on CAPS-data compared to only one in HGI-data. Insofar as we have succeeded in reducing data errors and increasing clinical homogeneity within pedigrees, this illustrates the possibility of increasing power even while reducing the number of affected individuals.

A substantial portion of the effect of our data regularization protocols on linkage results does indeed relate to the strict clinical criteria we chose, based on conservative definitions of Schizophrenia and Schizophrenia/Affective, to maximize uniformity across studies. This does not imply that all of the remaining families that are multiplex according to HGI-Pheno but not CAPS-Pheno (not included in these comparisons) are necessarily problematic or inappropriate for analysis. The full set of post-processing files for all CAPS-Geno pedigrees (including these CAPS non-multiplex families) are available through https://www.nimhgenetics.org/projects/CAPS, so that investigators can use their own clinical judgment in constituting data sets for future analyses. (Note too that many of the corrections made in the course of our work have been incorporated into subsequent releases of the standard HGI Schizophrenia data for Releases 8.01 and beyond.) However, insofar as clinical heterogeneity is in itself a problem for many complex disorders, this is an issue with far reaching implications, because the larger the desired sample size, the more prohibitive the cost of careful phenotyping becomes. It seems likely, therefore, that any deleterious effects of clinical heterogeneity also rise with sample size.

Of course, even the largest of the GSMA signals is still moderate in magnitude, and different patterns of comparative (HGI-data vs. CAPS-data) results might emerge for data showing much stronger linkage signals. Additionally, other data analytic approaches might yield different comparative results. We chose to rely here on GSMA for three primary reasons: (i) it allowed us to utilize separately estimated genetic maps for different population groups for CAPS-data; (ii) it is a commonly used method for analyzing multi-site genetic data; and (iii), our primary interest was in effects of data processing on meta-analysis per se. Another approach to analyzing multisite data is so-called “mega-analysis,” in which all data are simply pooled into a single data file for analysis, with all markers other than those used for a given study coded as missing all genotypes for families in that study. (This requires a common genetic map across studies.) For completeness, we include mega-analysis in Appendix S4. Results from mega-analysis are probably confounded by the particular configuration of missing marker data across studies, especially given the sparse marker maps used in the studies considered here. However, they corroborate the impact of data regularization both on peak size and rank ordering of loci.

It is of course not possible to extrapolate from the specific impacts of data regularization reported here to effects that might be seen in different data sets, perhaps analyzed using different statistical methods. Despite this caveat, we believe that the salient lessons learned from this exercise apply to other types of data that are processed through centralized repositories, including genome wide association and whole genome sequencing data. Errors and irregularities do accumulate in repositories, and differences across studies in clinical classification methods matter. It remains an open question whether in any particular application we can safely rely on increased sample size alone to overcome the impact on overall results of data irregularities and between-site differences, particularly with respect to phenotype definition, which are inevitable in the context of very large data collection and deposition projects.

## Supporting Information

Appendix S1 **Complete set of genotypic data processing histograms.** The histograms included for each study are Hardy-Weinberg p-values, initial and repeat missingness proportions for markers and individuals, and Mendel error counts by family and by marker. The number of observations, the range in values, and the number of observations over threshold are annotated within each histogram. Note that the range of the x-axis is data dependent and that the vertical dashed line indicates the threshold (shown when data range exceeds it).
(PDF)

Appendix S2 **Phenotypic data processing alogorithm including DSM codes.** The diagnostic algorithm converts sets of DSM codes into three working variables (SZ, SA, BS), which are in turn translated into affectedness status in the table provided. For both versions of the codes, DSM-IIIR and DSM-IV, the code sets used in the algorithm are then itemized; they are: SZ_CODES, SA_CODES, BS_CODES, GLOBAL_EXCLUDE, UNAFF_EXCLUDE, SZ_DEMOTE1, SZ_DEMOTE2, SA_EXCLUDE, SA_DEMOTE, BS_EXCLUDE.
(PDF)

Appendix S3 **Complete linkage results by subset over all processing states.** The labels for each line indicate state of phenotype (Pheno) and genotype (Geno) data, which can be Human Genetics Initiative (HGI) or Combined Analysis of Psychiatric Studies (CAPS).
(PDF)

Appendix S4 **Meta-analysis versus pooled mega-analysis.**
(PDF)

Table S1 **Complete list of family IDs and processing status indicators.** Column header descriptions are as follows: dataset ID and family ID refer to NIMH Human Genetics Initiative (HGI) identifiers; merged indicates whether pedigree was joined during genotype processing by Combined Analysis of Psychiatric Studies (CAPS); CAPS-Geno indicates whether pedigree passed genotype regularization, and the excess Mendel or relationship error columns show reasons for failure; multiplex refers to families meeting both CAPS and HGI multiplex criteria; high bitsize indicates 16 pedigrees with bitsize of 25 or more; analysis indicates the 1,020 pedigrees used in this study, i.e., those passing CAPS-Geno, multiplex and not high bitsize.
(PDF)
