# Supplementary material for: Meta-Analysis of Repository Data: Impact of Data Regularization on NIMH Schizophrenia Linkage Results
Source: PLoS One. 2014 Jan 14;9(1):e84696. doi: 10.1371/journal.pone.0084696 (PMC3891773; doi:10.1371/journal.pone.0084696)

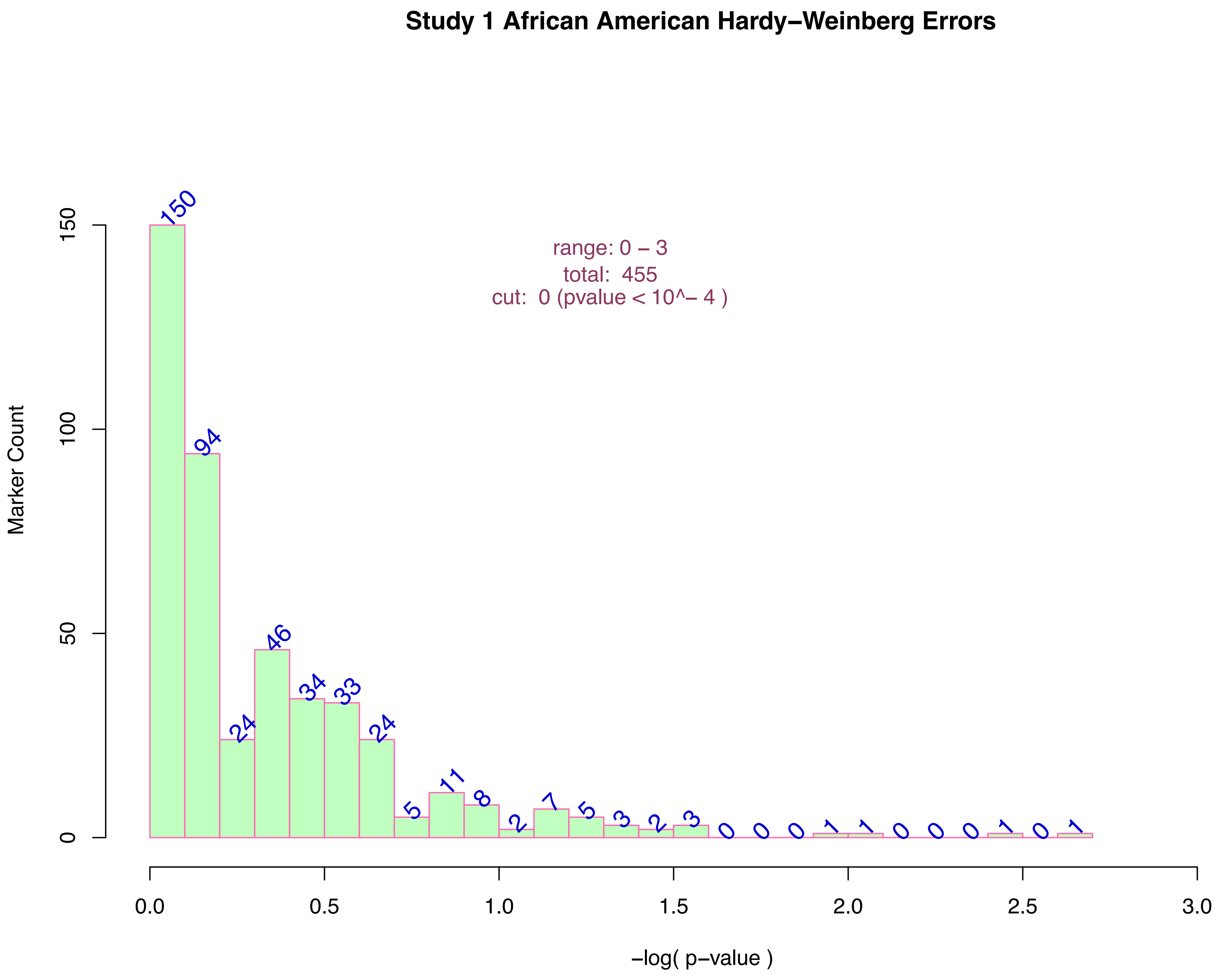

Study 1 African American Hardy−Weinberg Errors
range: 0 − 3
total: 455
cut: 0 (pvalue < 10^− 4)
Marker Count
−log(p−value)
0
50
100
150
0.0
0.5
1.0
1.5
2.0
2.5
3.0
150
94
24
46
34
33
24
5
11
8
2
7
5
3
2
3
0
0
0
1
1
0
0
0
1
0
1

Study 1 African American Initial Mendel Errors

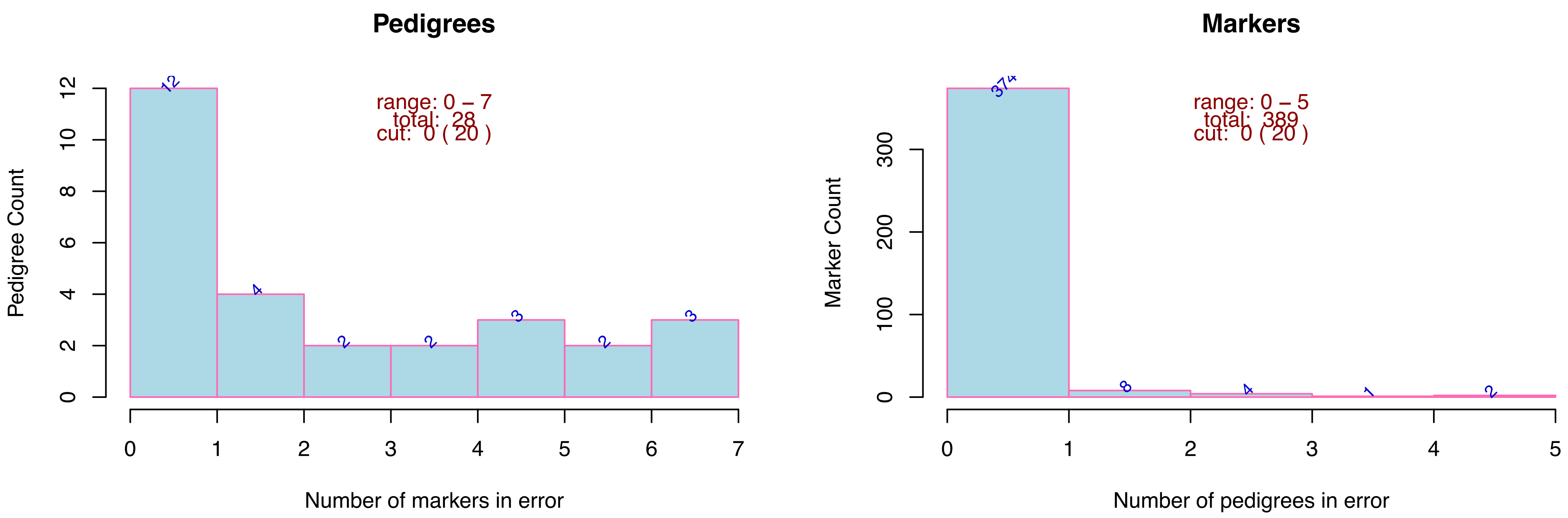

Study 1 African American Initial Missingness

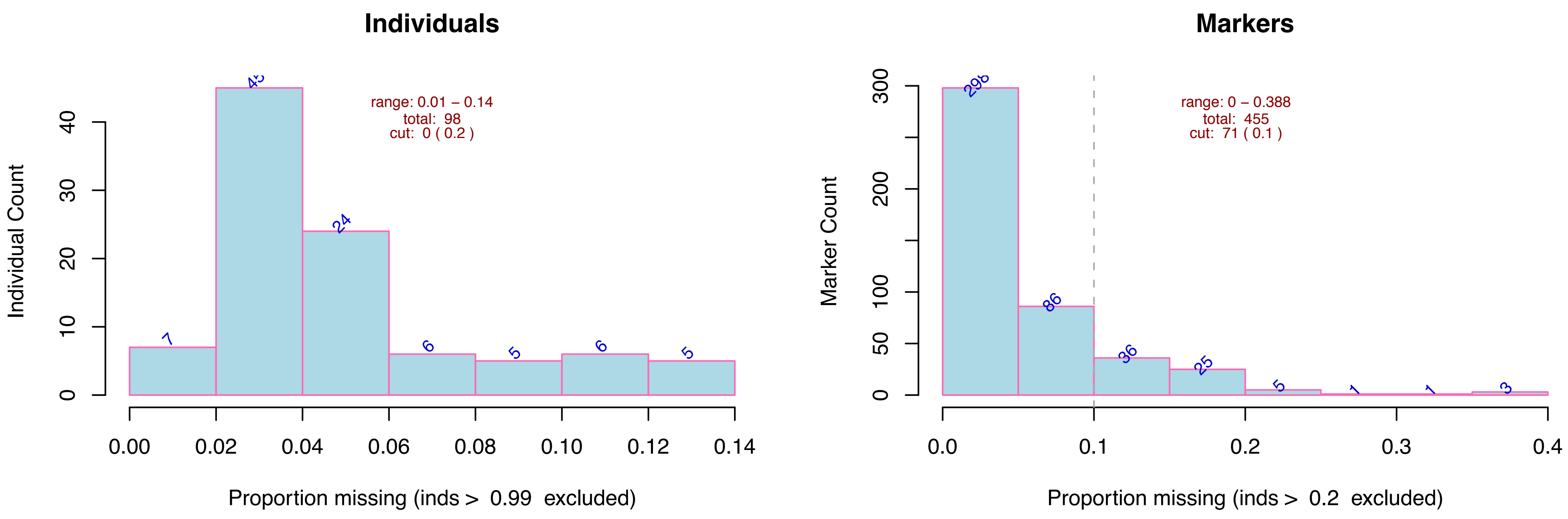

Study 1 African American Repeat Missingness

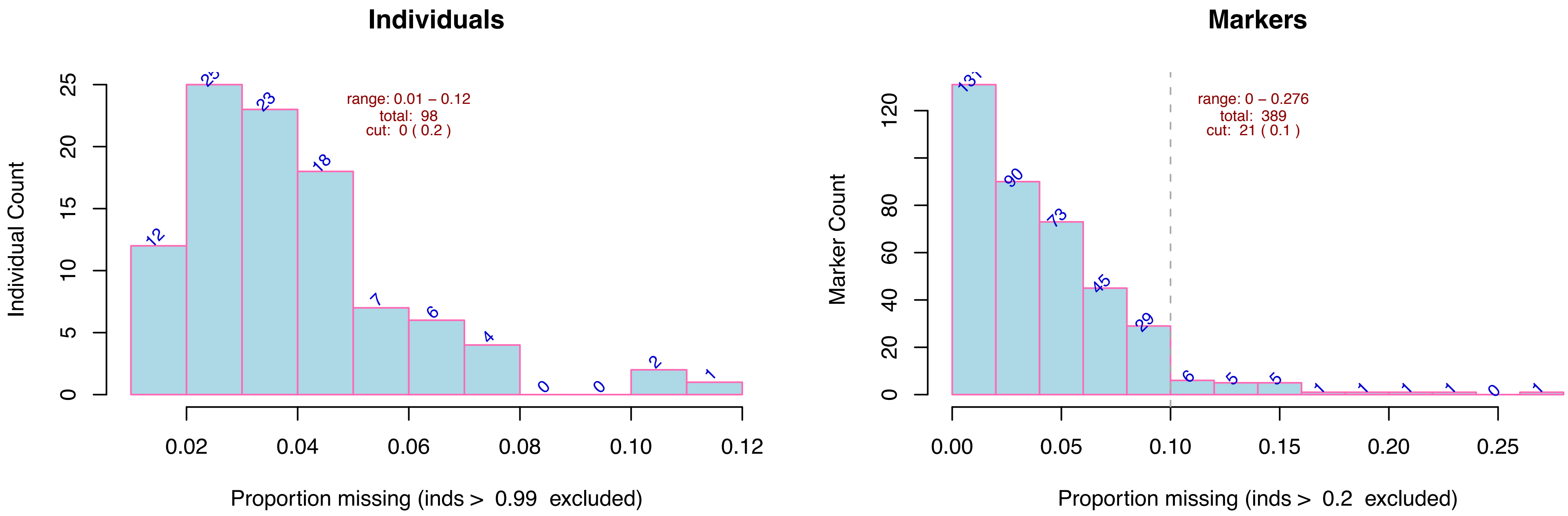

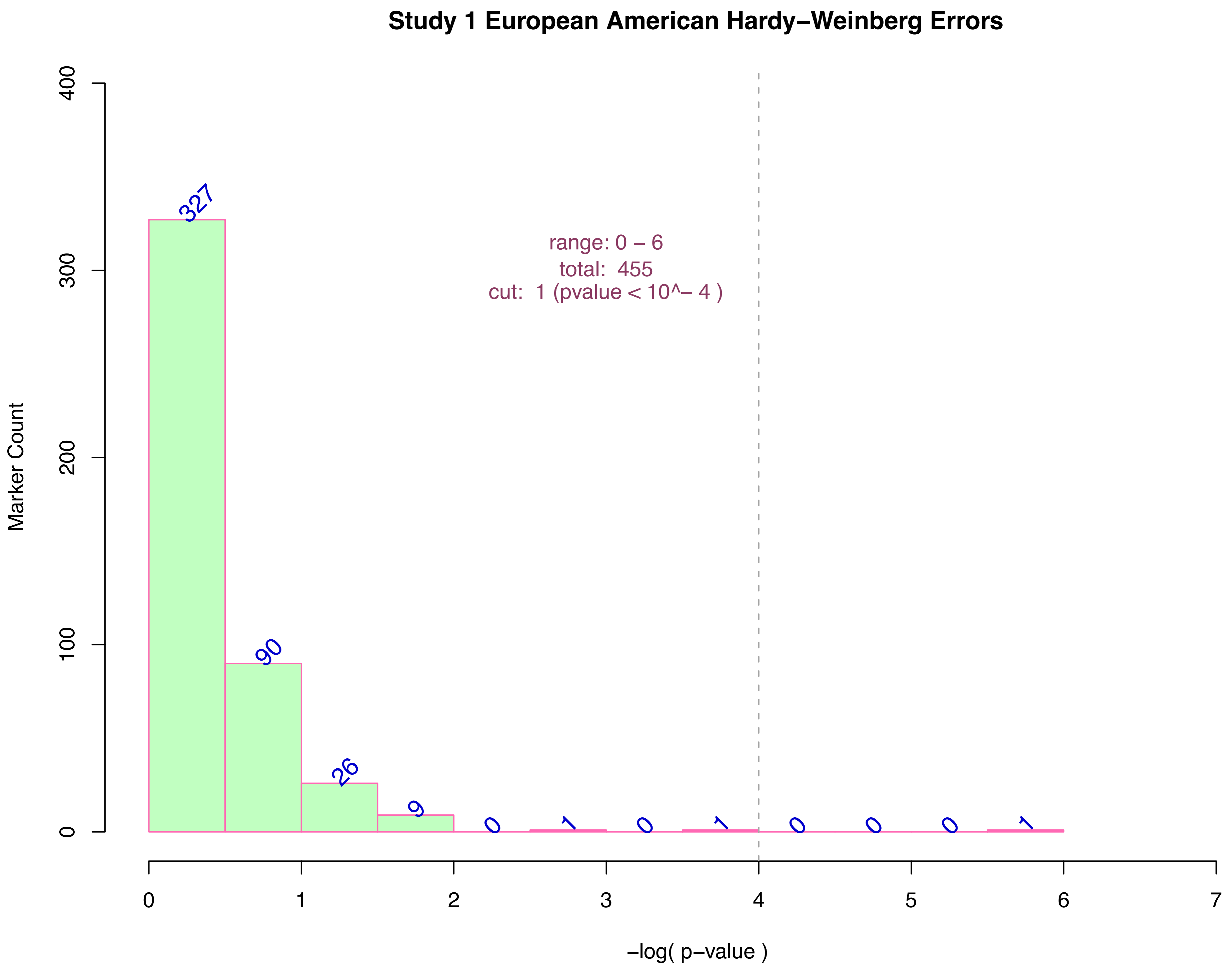

Study 1 European American Hardy−Weinberg Errors
Marker Count
−log(p−value)
range: 0 − 6
total: 455
cut: 1 (pvalue < 10^− 4)
327
90
26
9
0
1
0
1
0
0
0
1
0
100
200
300
400
0
1
2
3
4
5
6
7

Study 1 European American Initial Mendel Errors

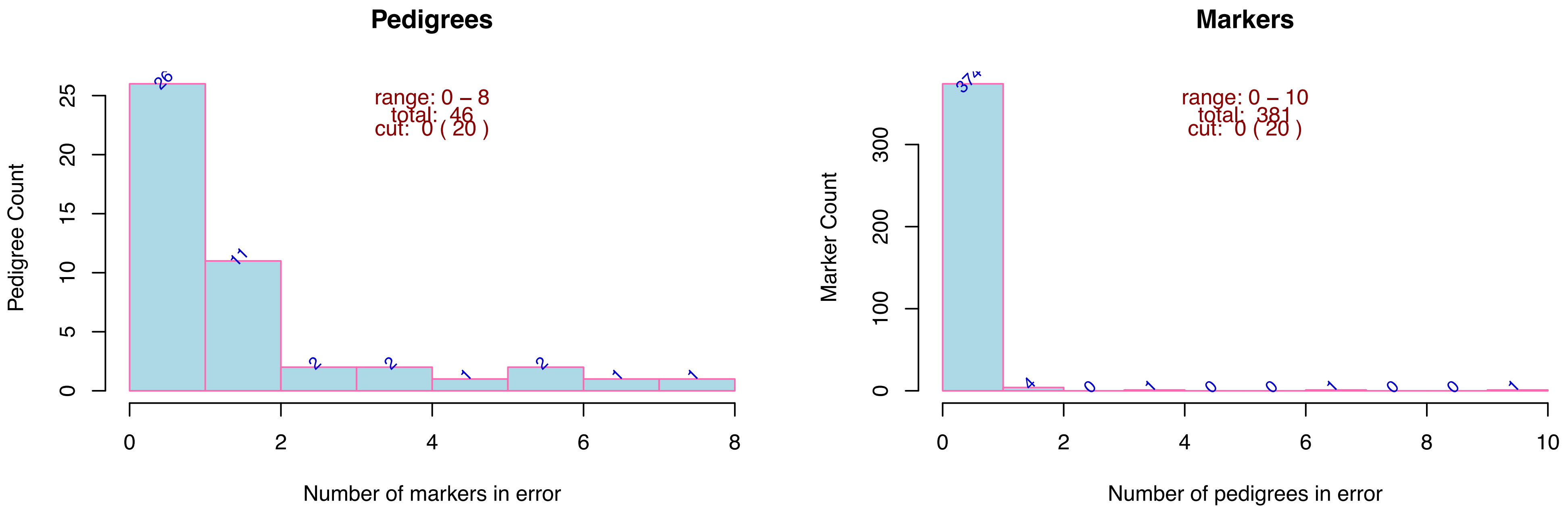

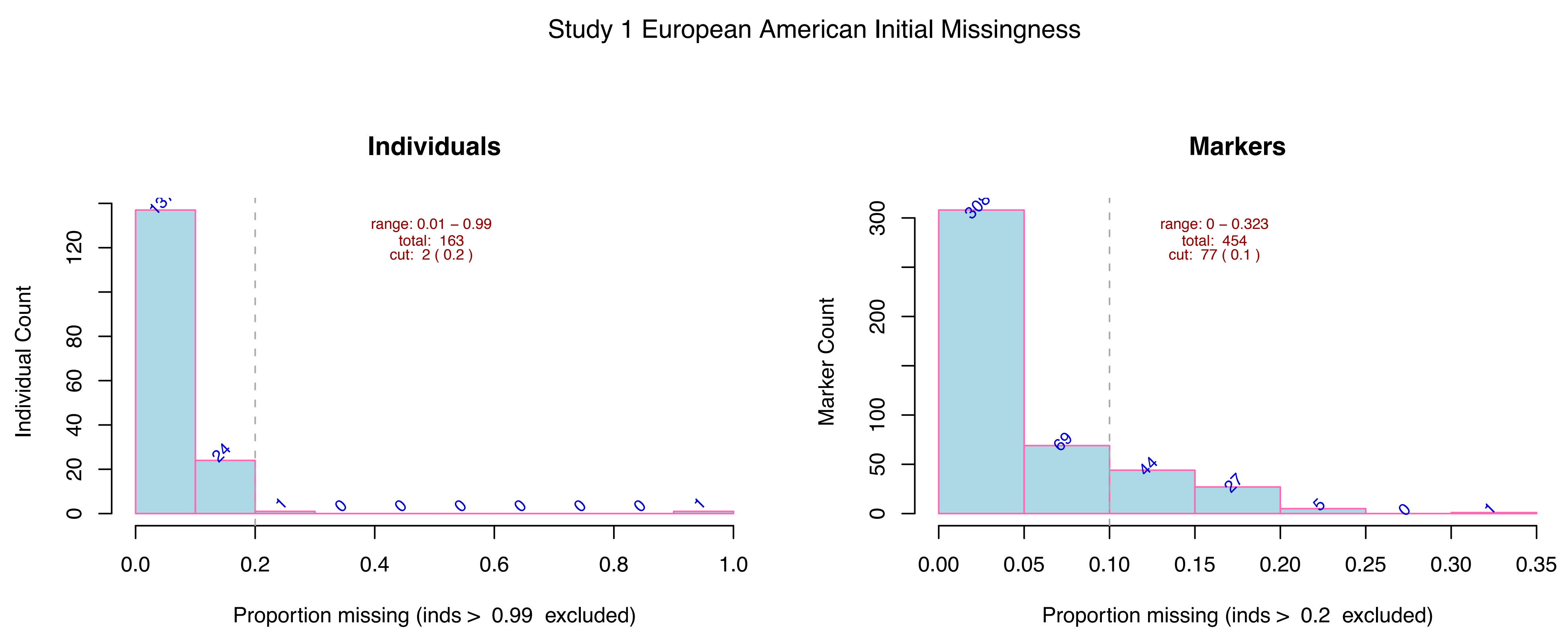
Study 1 European American Initial Missingness
Individuals
range: 0.01 – 0.99
total: 163
cut: 2 (0.2)
Individual Count
Proportion missing (inds > 0.99 excluded)
0
20
40
60
80
120
0.0
0.2
0.4
0.6
0.8
1.0
24
1
0
0
0
0
0
0
1
Markers
range: 0 – 0.323
total: 454
cut: 77 (0.1)
Marker Count
Proportion missing (inds > 0.2 excluded)
0
50
100
200
300
0.00
0.05
0.10
0.15
0.20
0.25
0.30
0.35
69
44
27
5
0
1

Study 1 European American Repeat Missingness

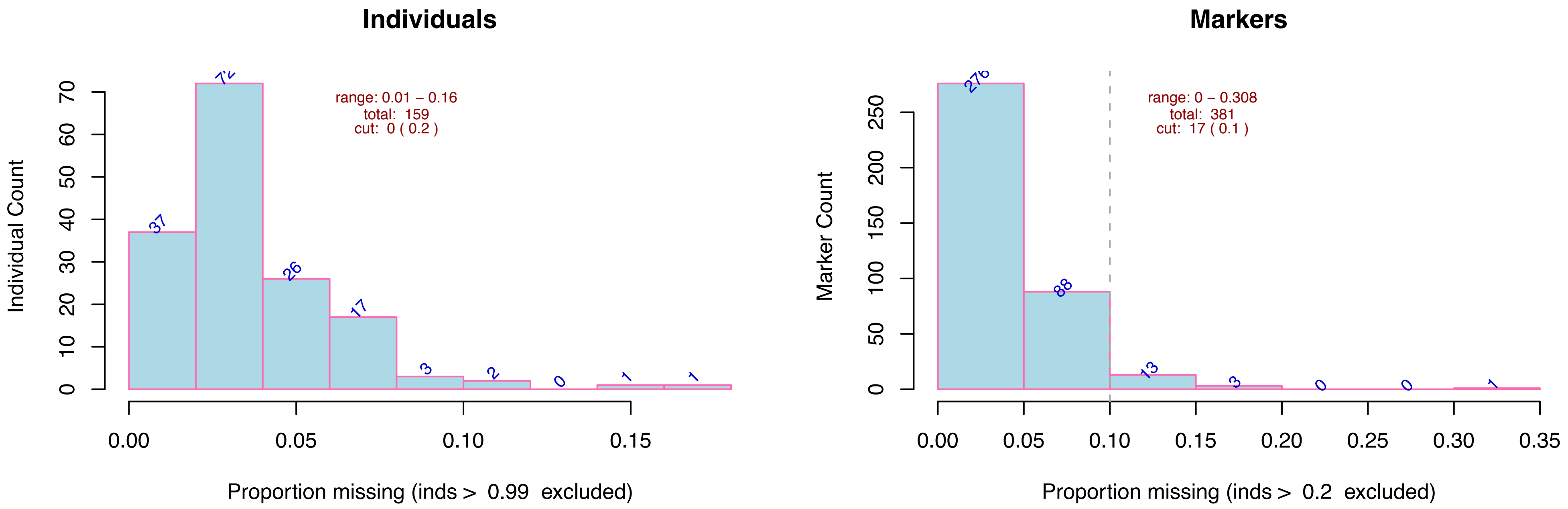

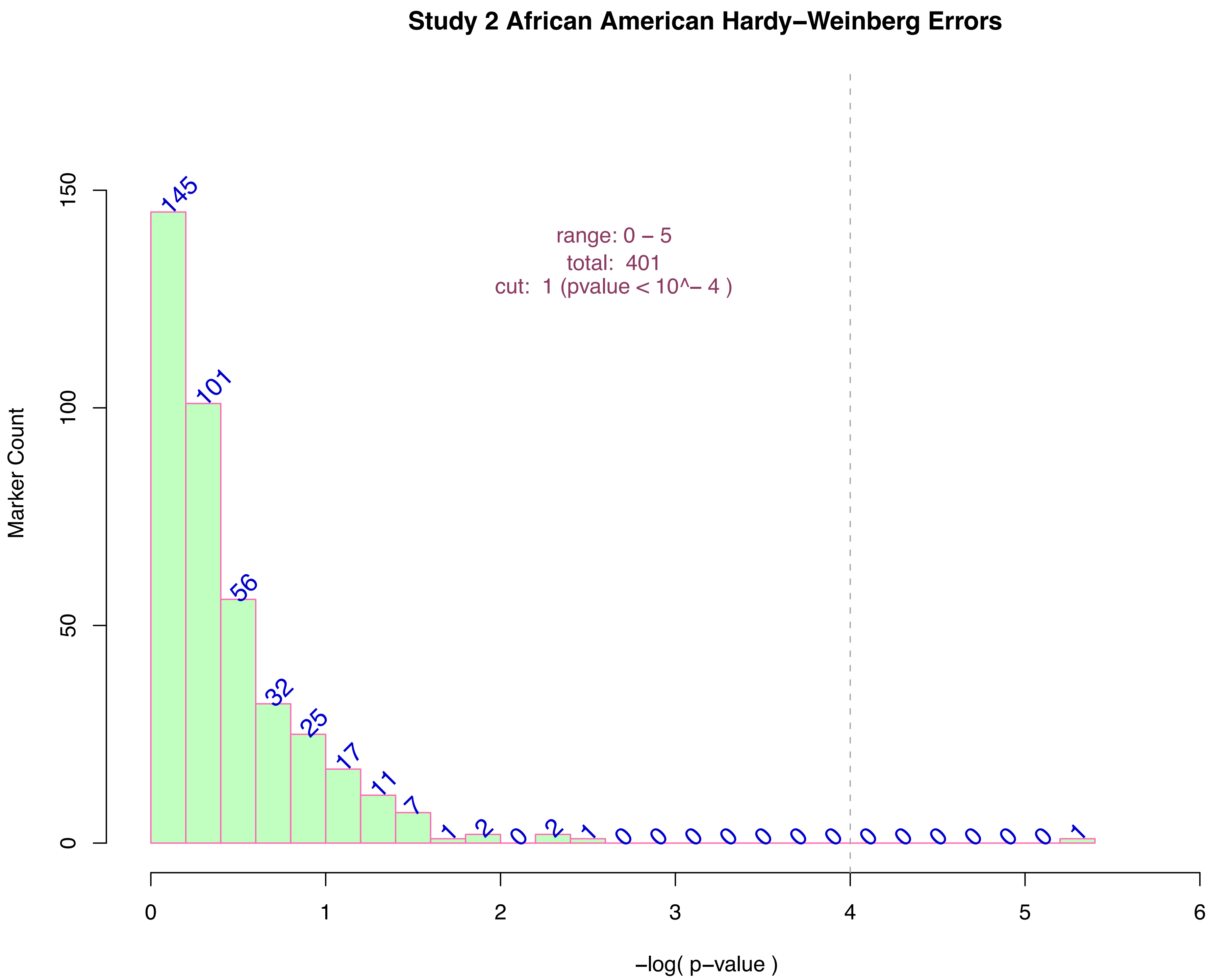
Study 2 African American Hardy−Weinberg Errors
range: 0 – 5
total: 401
cut: 1 (pvalue < 10^– 4)
Marker Count
−log(p−value)
0
50
100
150
0
1
2
3
4
5
6
145
101
56
32
25
17
11
7
1
2
0
2
1
0
0
0
0
0
0
0
0
0
0
0
0
0
0
1

Study 2 African American Initial Mendel Errors

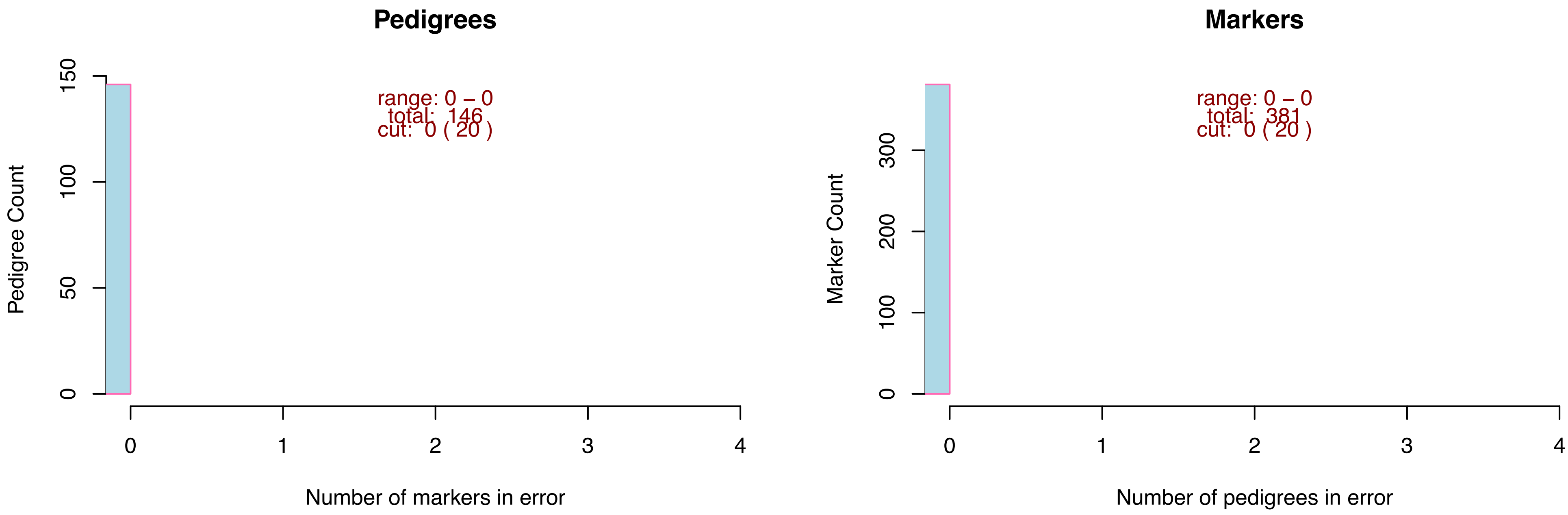

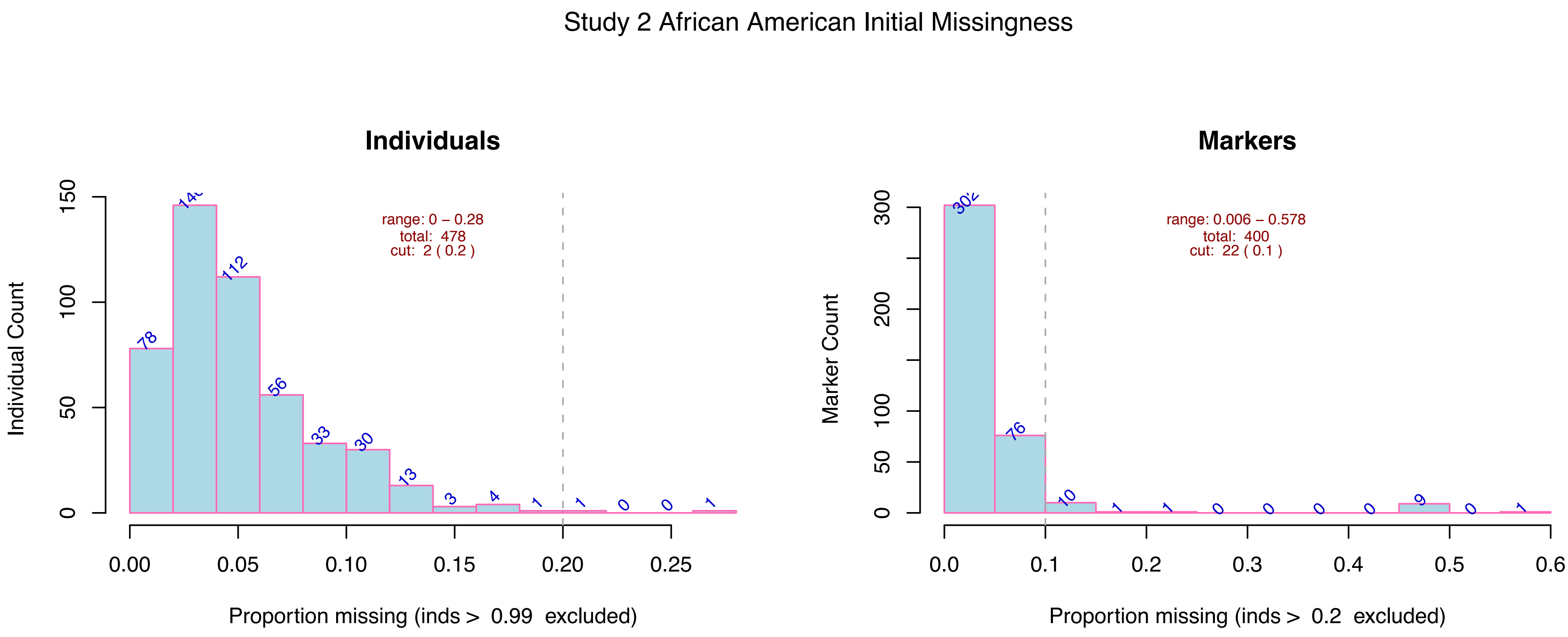
Study 2 African American Initial Missingness
Individuals
range: 0 – 0.28
total: 478
cut: 2 (0.2)
Individual Count
Proportion missing (inds > 0.99 excluded)
78
112
56
33
30
13
3
4
1
1
0
0
1
0.00
0.05
0.10
0.15
0.20
0.25
0
50
100
150
Markers
range: 0.006 – 0.578
total: 400
cut: 22 (0.1)
Marker Count
Proportion missing (inds > 0.2 excluded)
76
10
1
1
0
0
0
0
9
0
1
0.0
0.1
0.2
0.3
0.4
0.5
0.6
0
50
100
200
300

Study 2 African American Repeat Missingness

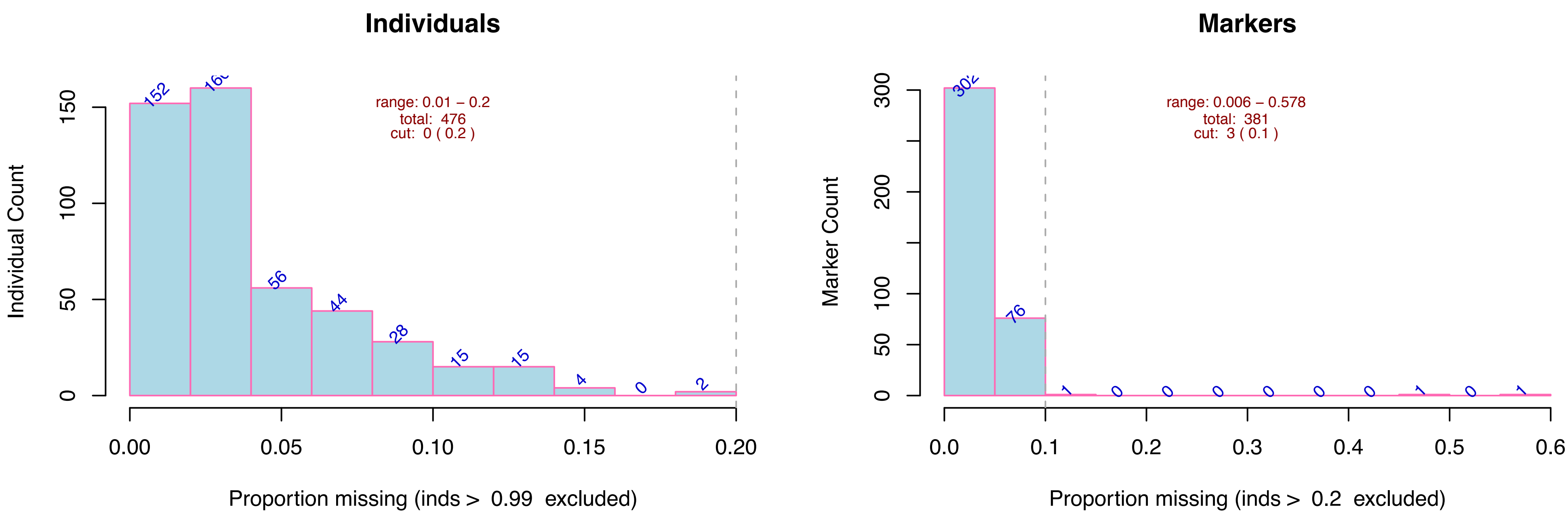

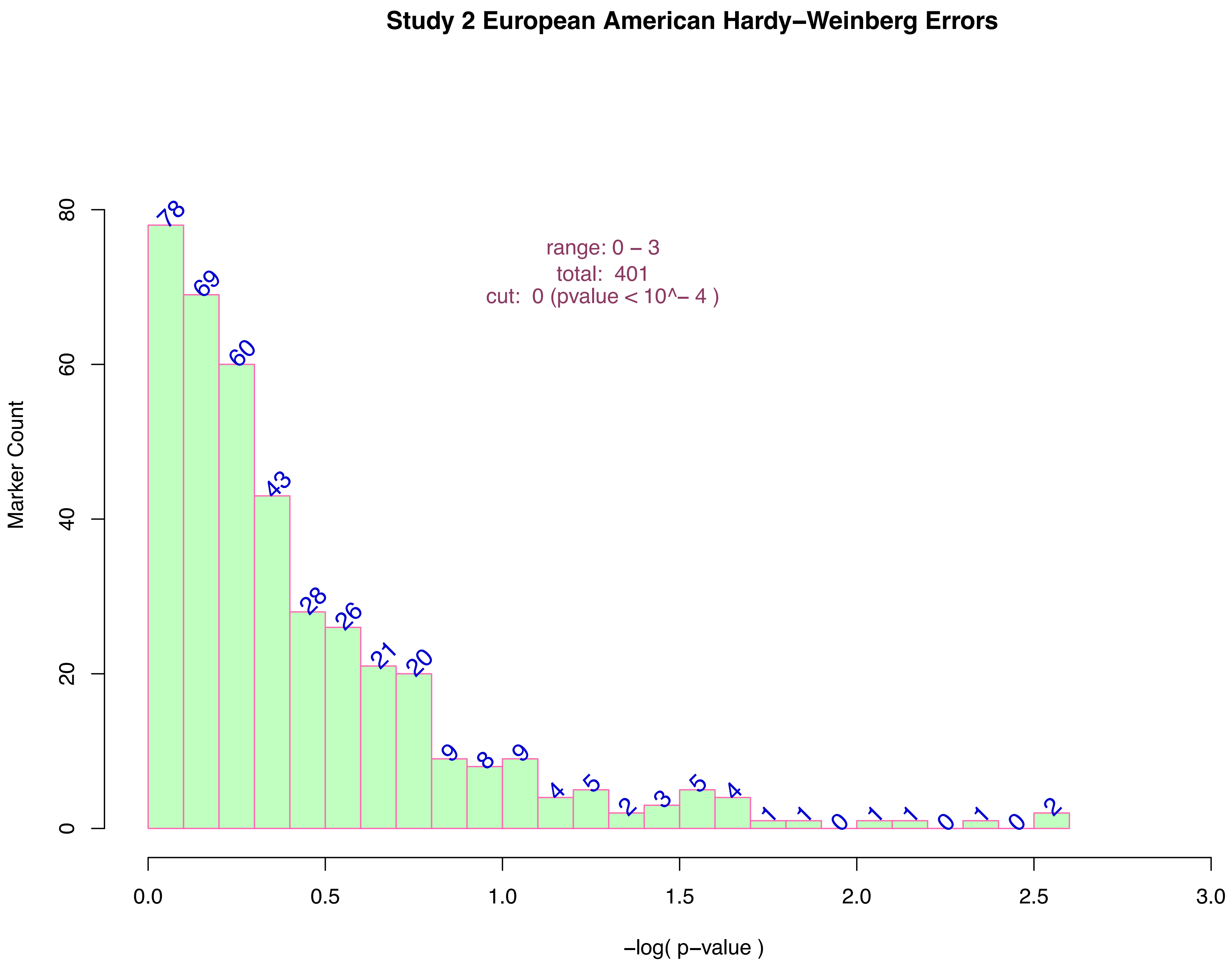

Study 2 European American Hardy−Weinberg Errors
range: 0 − 3
total: 401
cut: 0 (pvalue < 10^− 4)
Marker Count
0
20
40
60
80
78
69
60
43
28
26
21
20
9
8
9
4
5
2
3
5
4
1
1
0
1
1
0
1
0
2
0.0
0.5
1.0
1.5
2.0
2.5
3.0
−log(p−value)

Study 2 European American Initial Mendel Errors

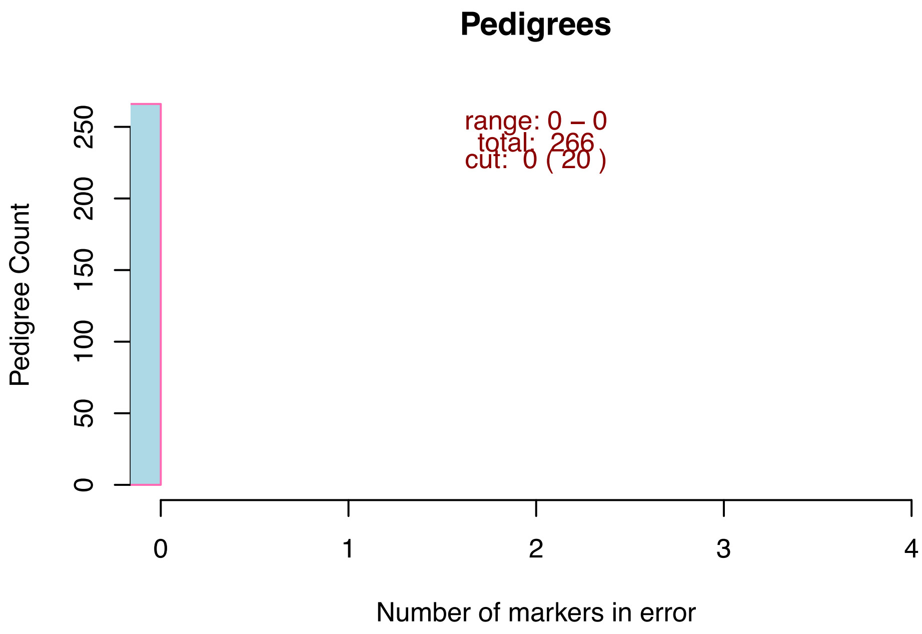

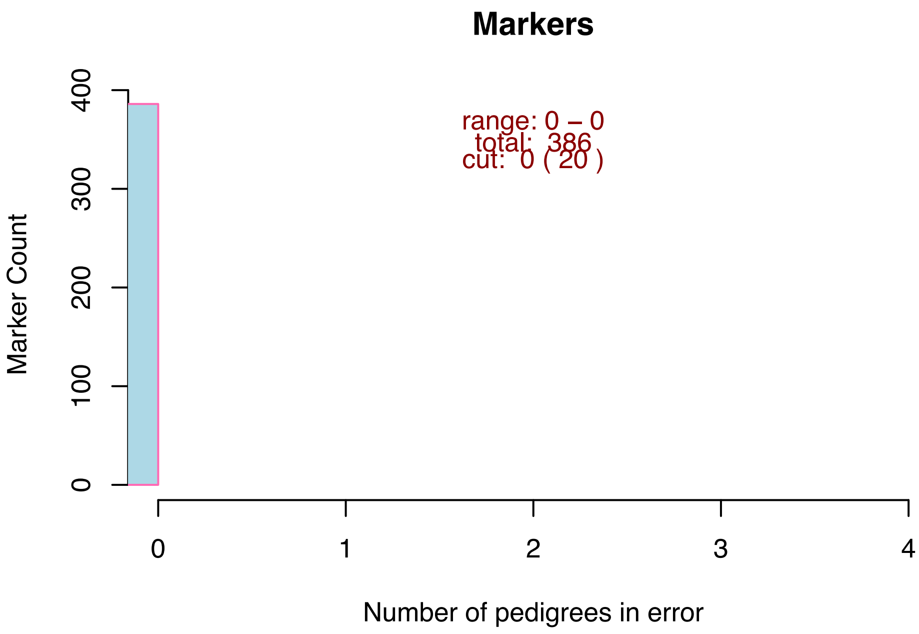

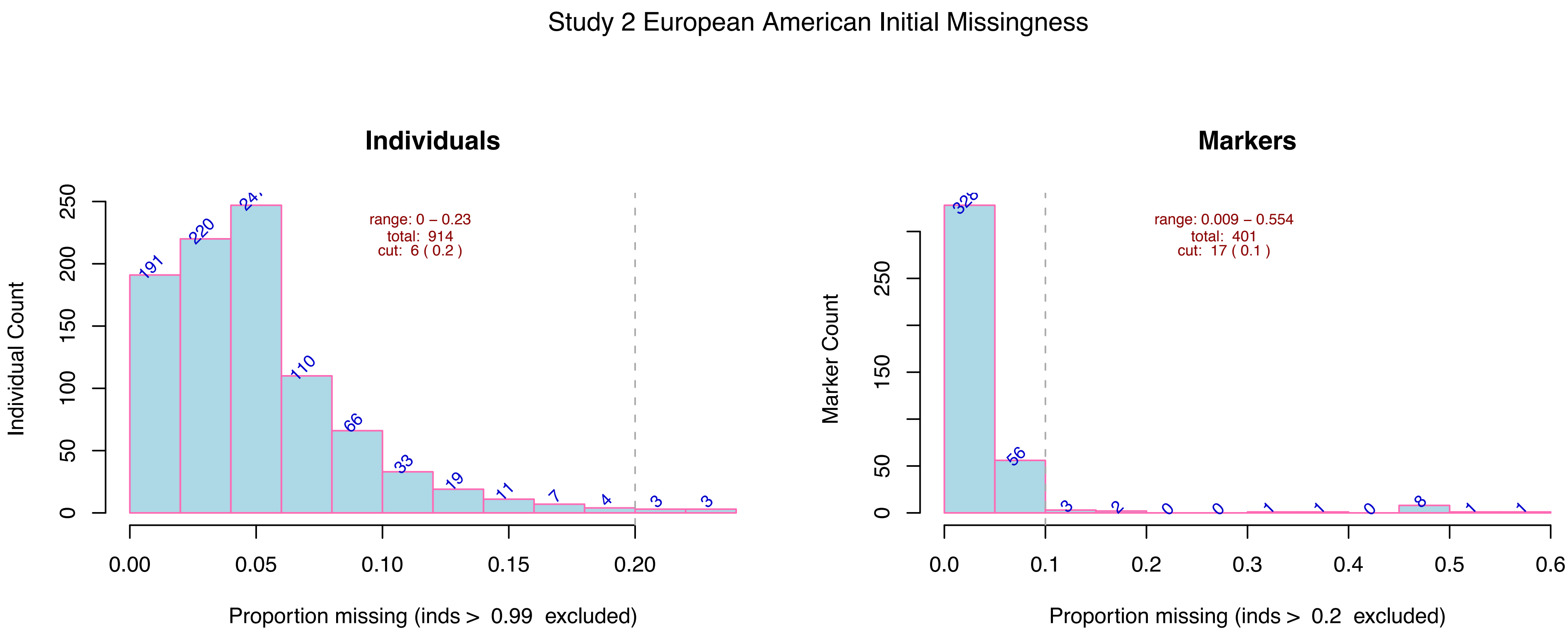

Study 2 European American Initial Missingness
Individuals
Individual Count
Proportion missing (inds > 0.99 excluded)
range: 0 – 0.23
total: 914
cut: 6 (0.2)
Markers
Marker Count
Proportion missing (inds > 0.2 excluded)
range: 0.009 – 0.554
total: 401
cut: 17 (0.1)

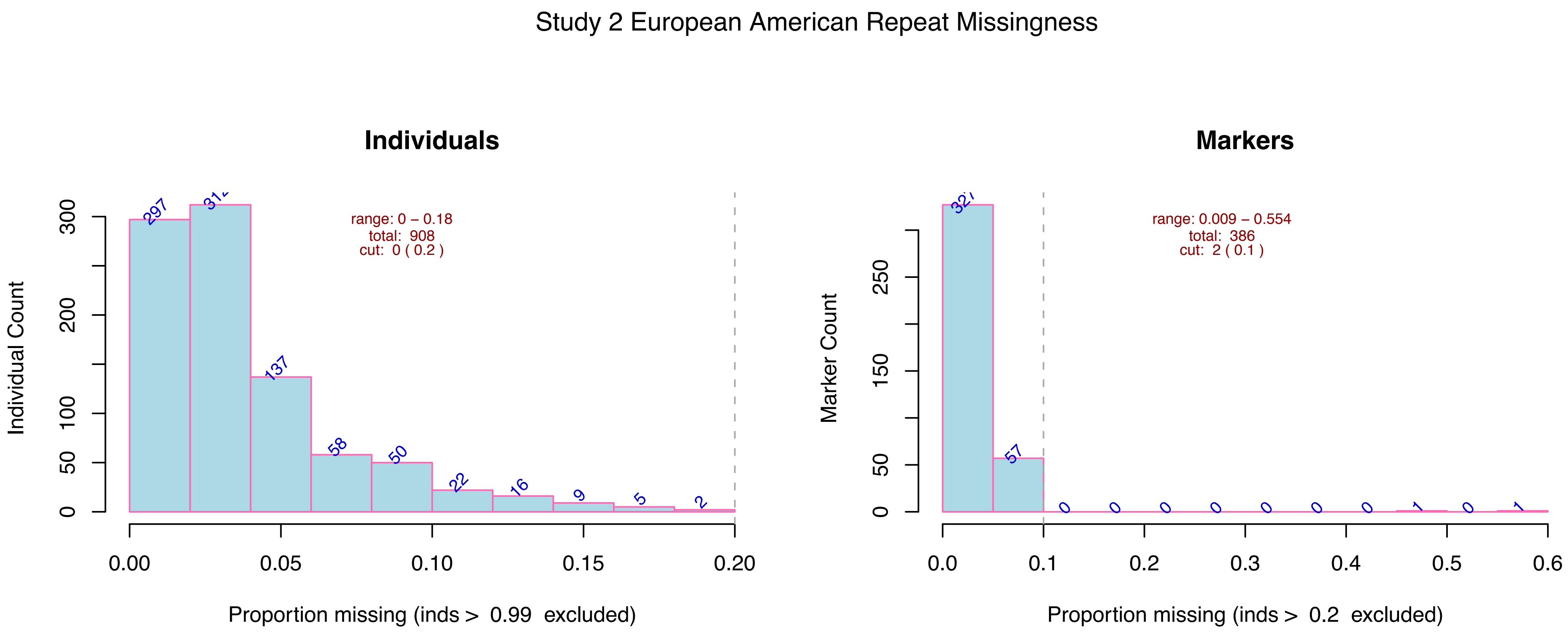
Study 2 European American Repeat Missingness
Individuals
Individual Count
Proportion missing (inds > 0.99 excluded)
range: 0 – 0.18
total: 908
cut: 0 (0.2)
297
137
58
50
22
16
9
5
2
Markers
Marker Count
Proportion missing (inds > 0.2 excluded)
range: 0.009 – 0.554
total: 386
cut: 2 (0.1)
57
0.00
0.05
0.10
0.15
0.20
0.0
0.1
0.2
0.3
0.4
0.5
0.6

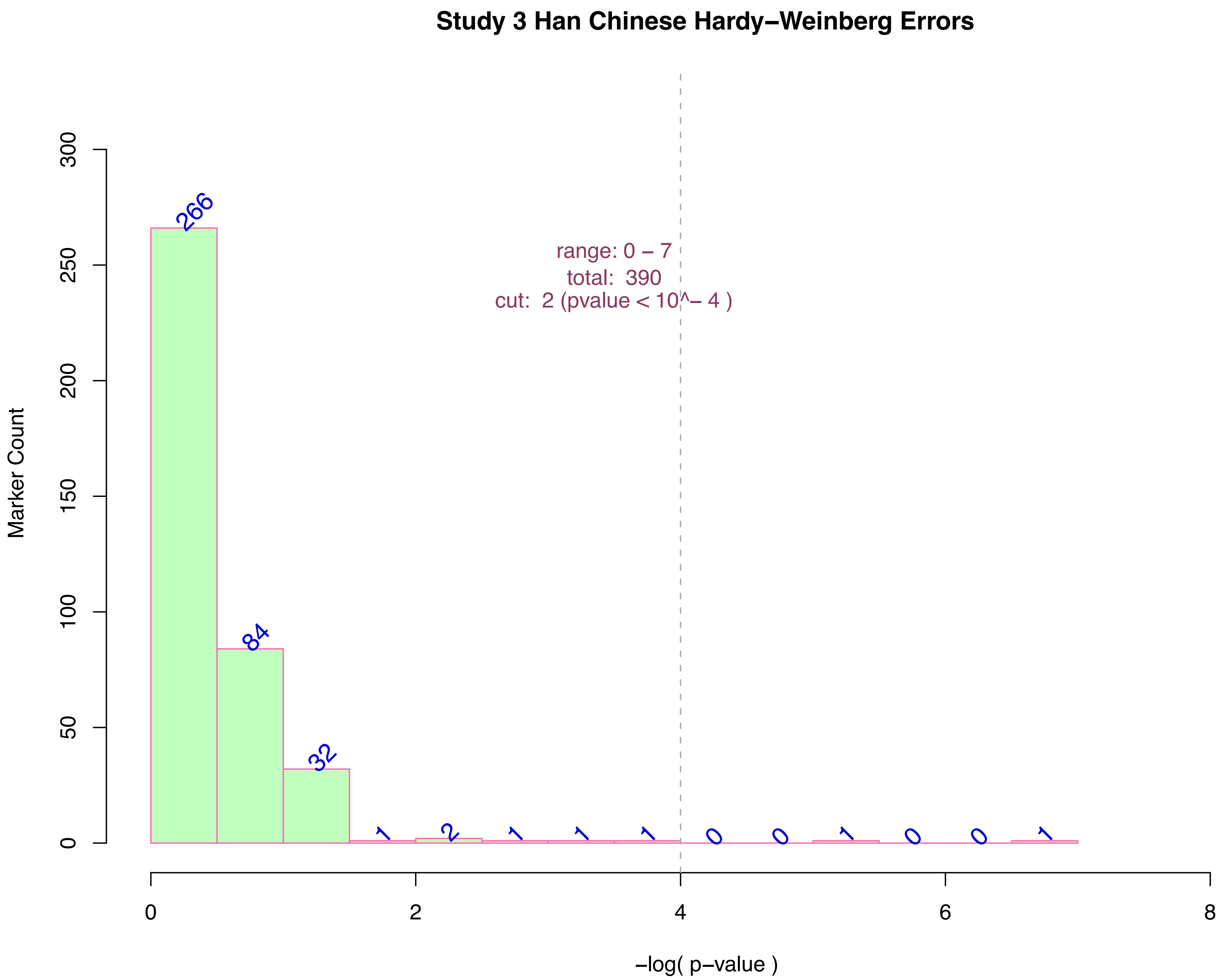
Study 3 Han Chinese Hardy−Weinberg Errors
range: 0 – 7
total: 390
cut: 2 (pvalue < 10^− 4)
Marker Count
−log(p−value)
0
50
100
150
200
250
300
0
2
4
6
8
266
84
32
1
2
1
1
1
0
0
1
0
0
1

Study 3 Han Chinese Initial Mendel Errors

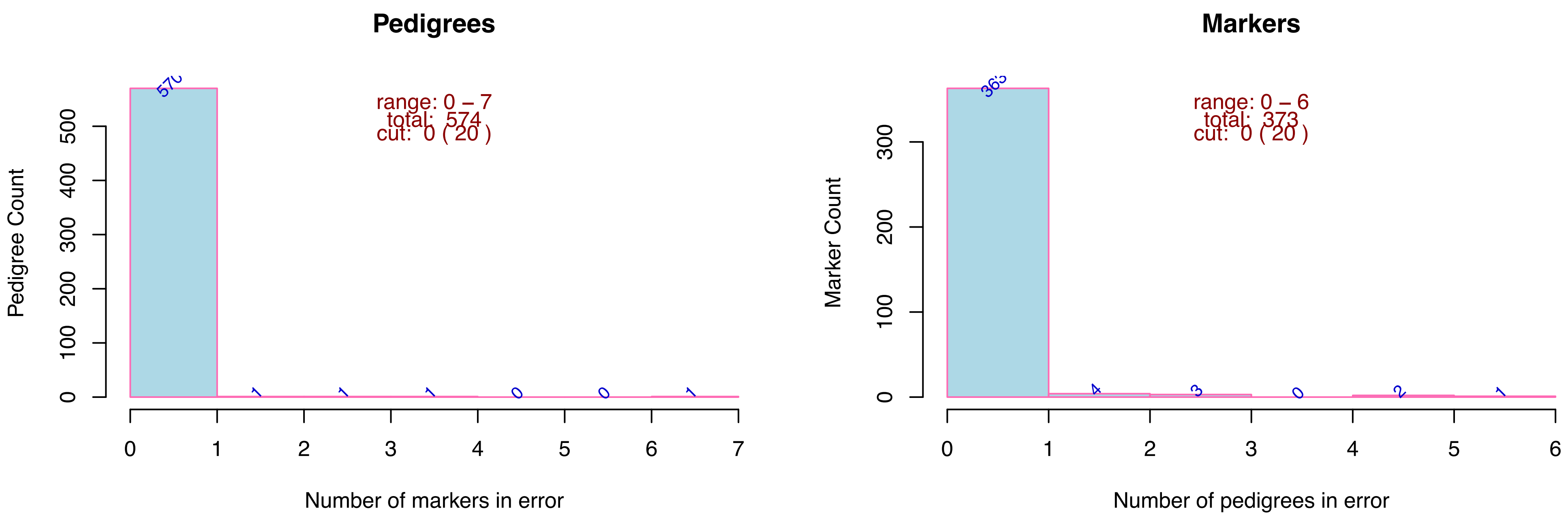

Study 3 Han Chinese Initial Missingness

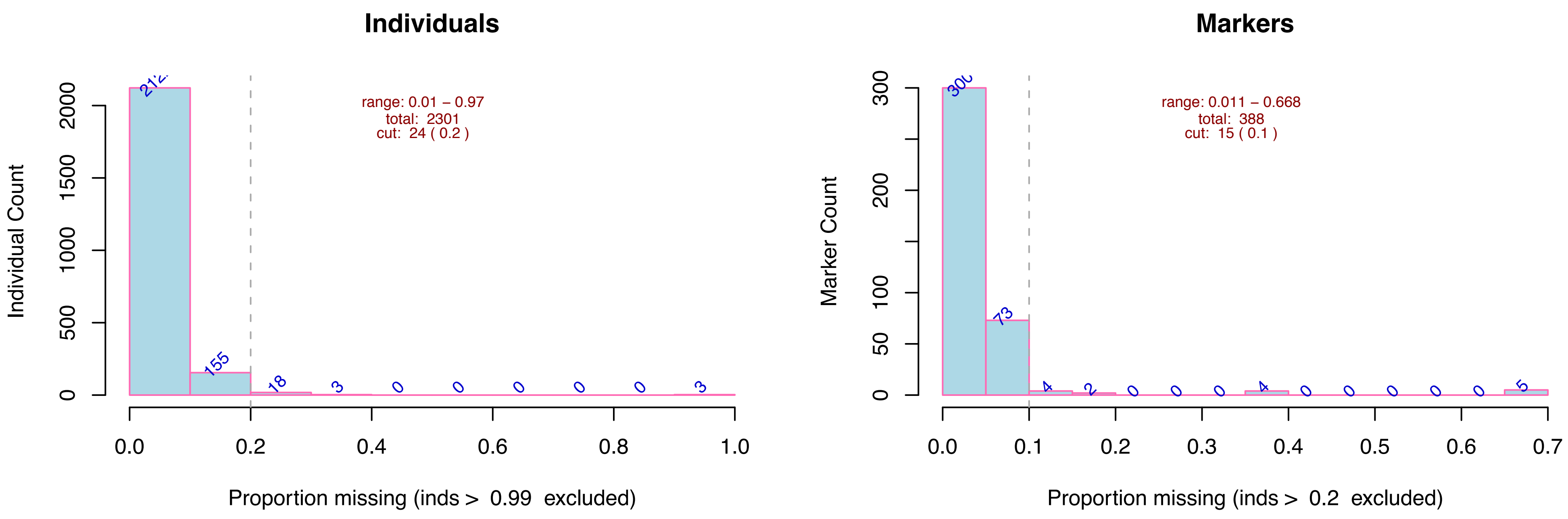

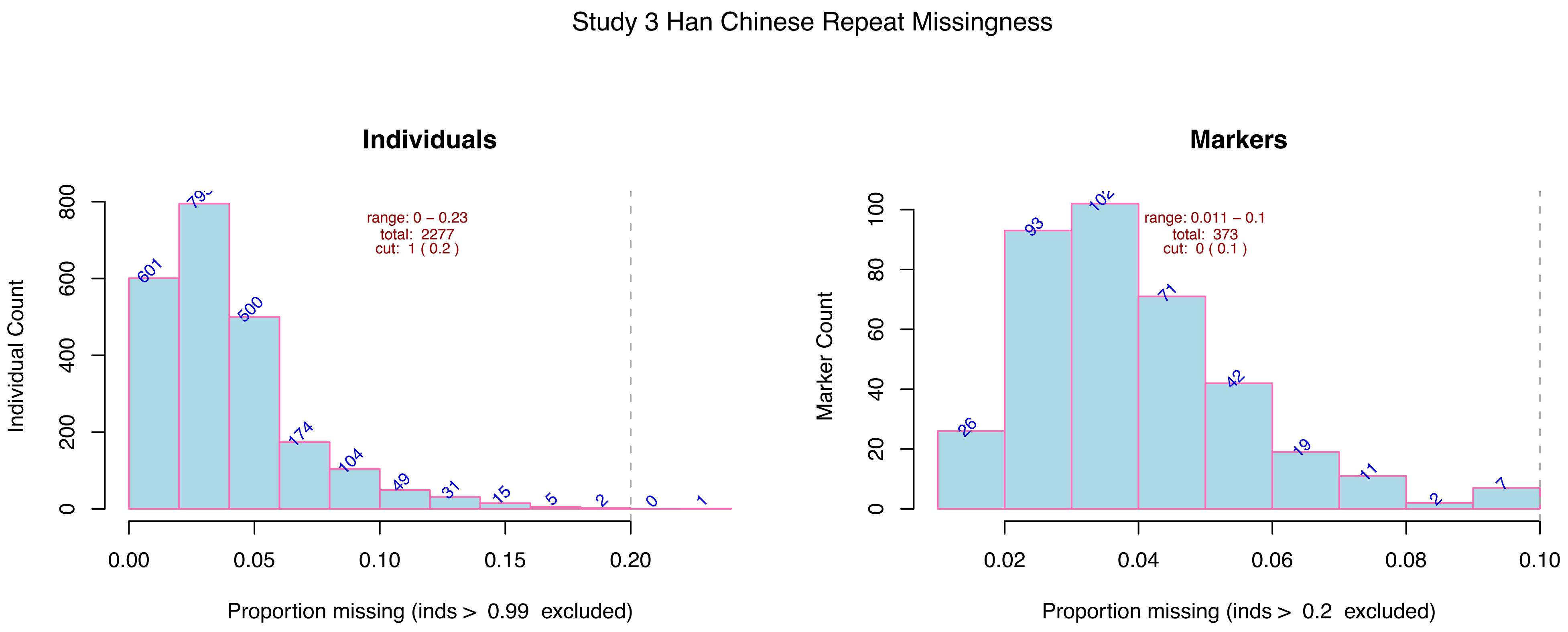

Study 3 Han Chinese Repeat Missingness
Individuals
range: 0 – 0.23
total: 2277
cut: 1 (0.2)
Individual Count
0
200
400
600
800
601
500
174
104
49
31
15
5
2
0
1
0.00
0.05
0.10
0.15
0.20
Proportion missing (inds > 0.99 excluded)
Markers
range: 0.011 – 0.1
total: 373
cut: 0 (0.1)
Marker Count
0
20
40
60
80
100
26
93
71
42
19
11
2
7
0.02
0.04
0.06
0.08
0.10
Proportion missing (inds > 0.2 excluded)

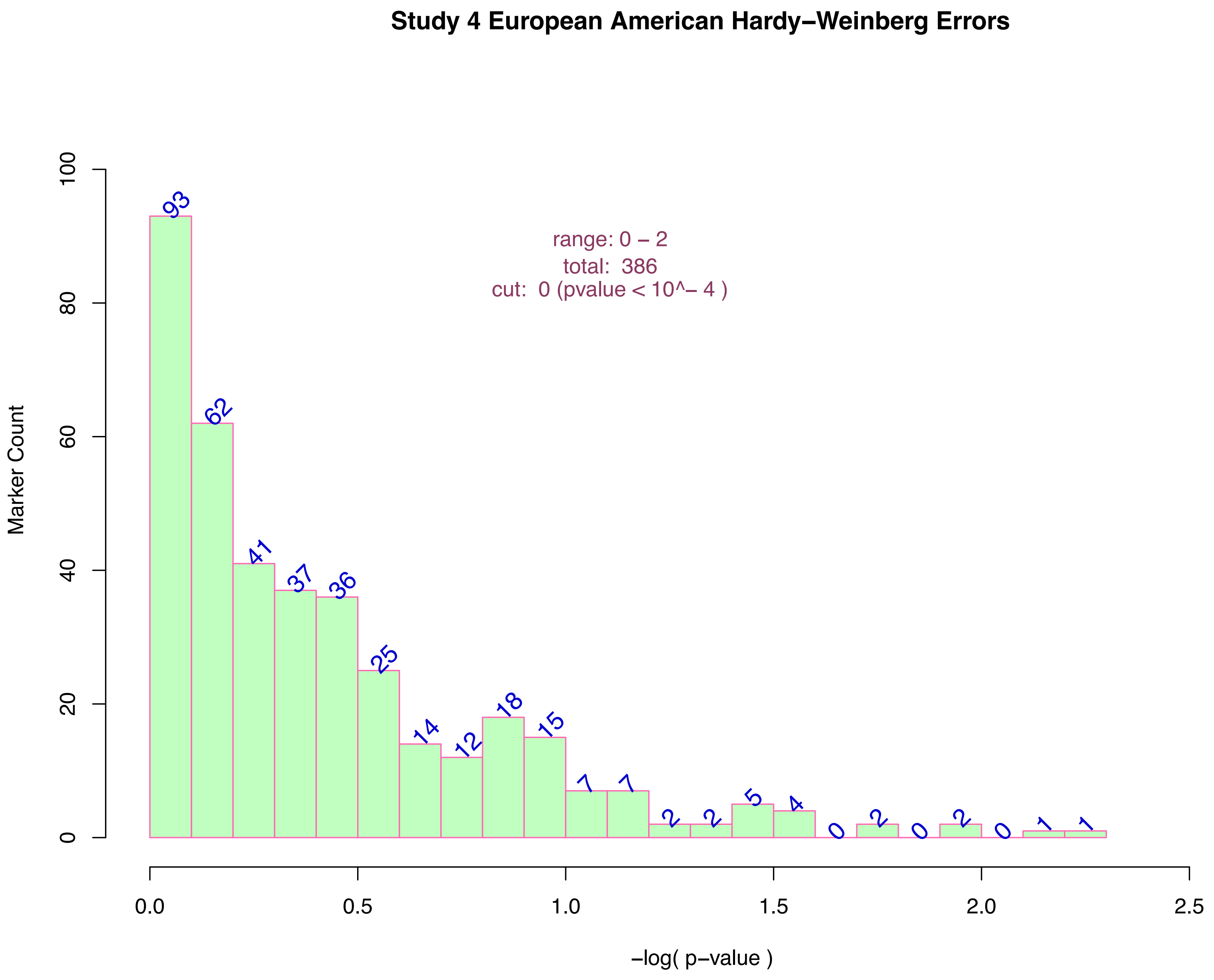
Study 4 European American Hardy−Weinberg Errors
range: 0 – 2
total: 386
cut: 0 (pvalue < 10^– 4)
Marker Count
−log(p−value)
0
20
40
60
80
100
0.0
0.5
1.0
1.5
2.0
2.5
93
62
41
37
36
25
14
12
18
15
7
7
2
2
5
4
0
2
0
2
0
1
1

Study 4 European American Initial Mendel Errors

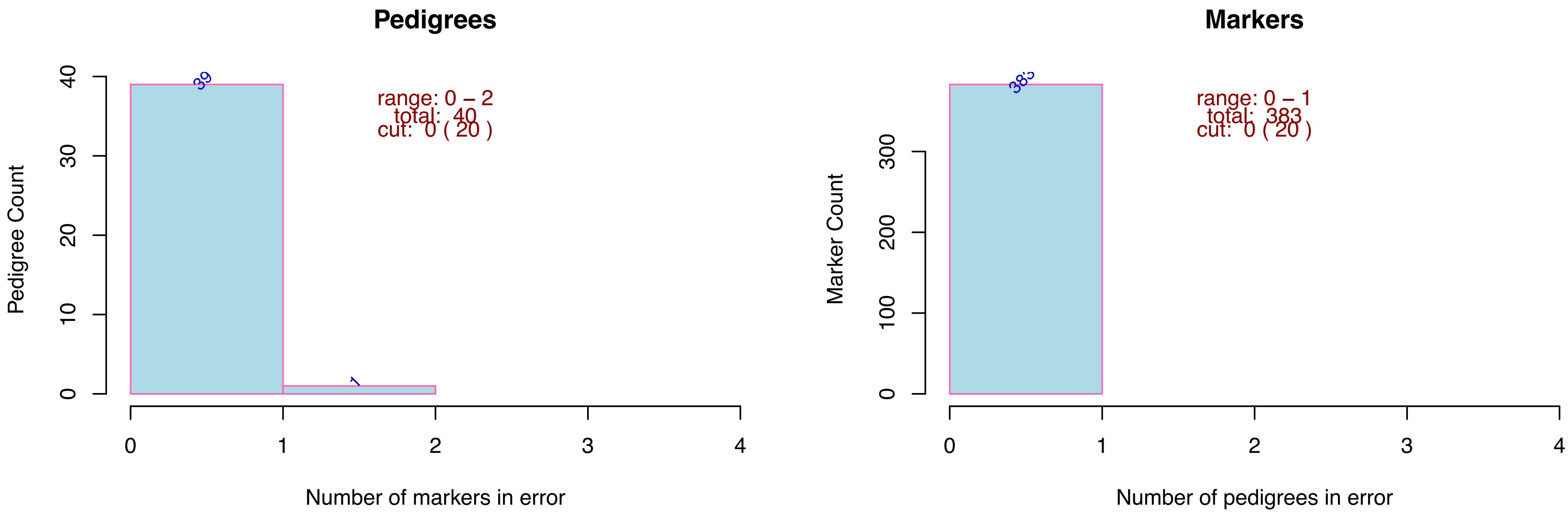

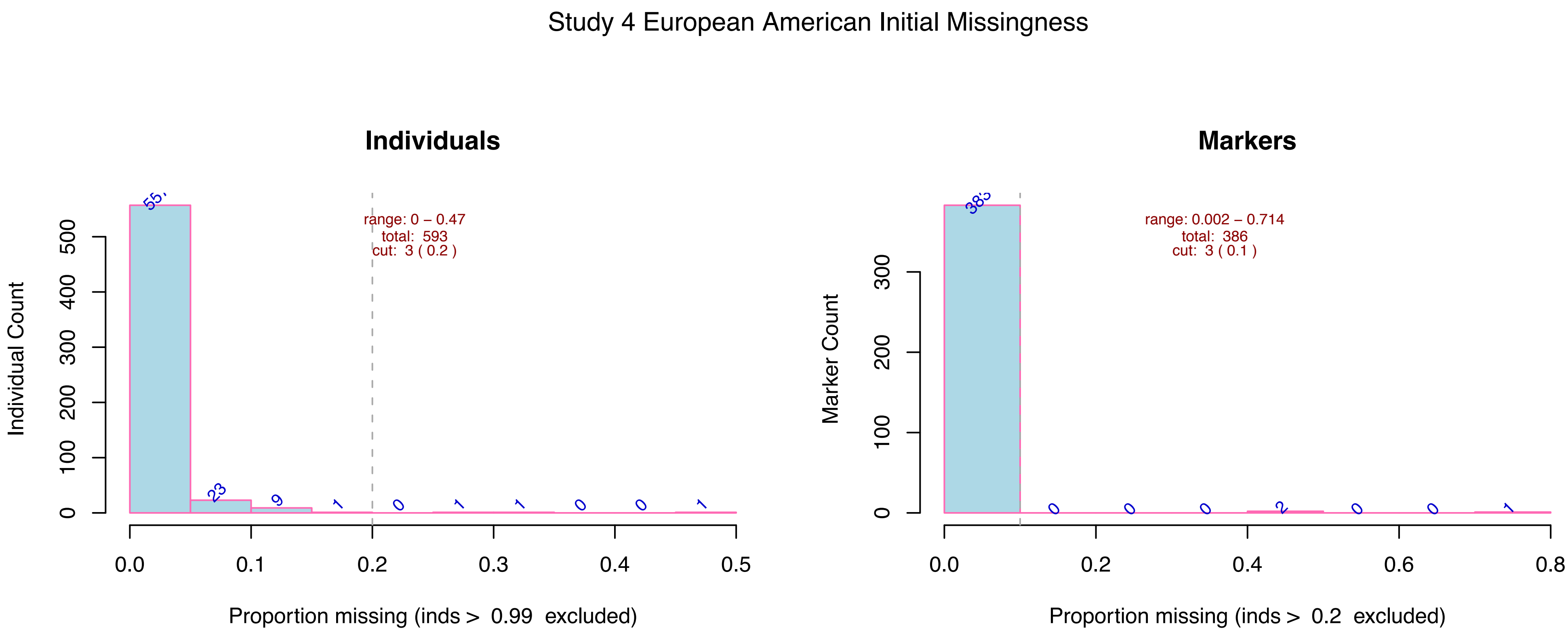
Study 4 European American Initial Missingness
Individuals
range: 0 – 0.47
total: 593
cut: 3 (0.2)
Individual Count
Proportion missing (inds > 0.99 excluded)
0 100 200 300 400 500
0.0 0.1 0.2 0.3 0.4 0.5
23 9 1 0 1 1 0 0 1
Markers
range: 0.002 – 0.714
total: 386
cut: 3 (0.1)
Marker Count
Proportion missing (inds > 0.2 excluded)
0 100 200 300
0.0 0.2 0.4 0.6 0.8
0 0 0 2 0 0 1

Study 4 European American Repeat Missingness

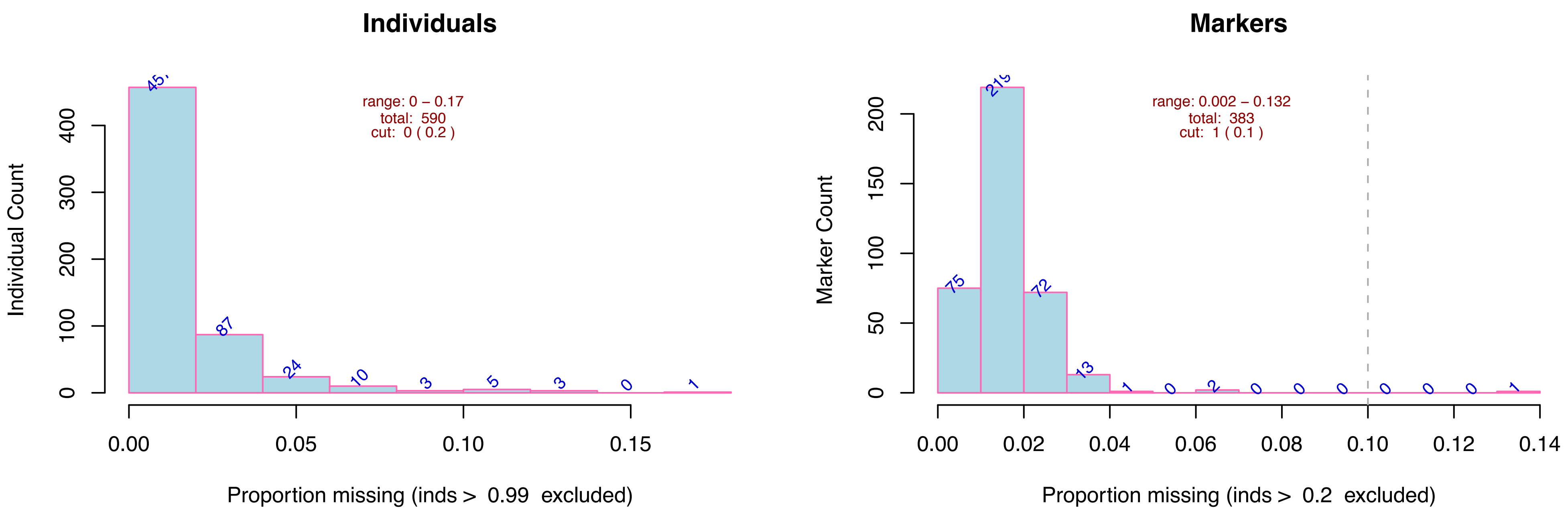

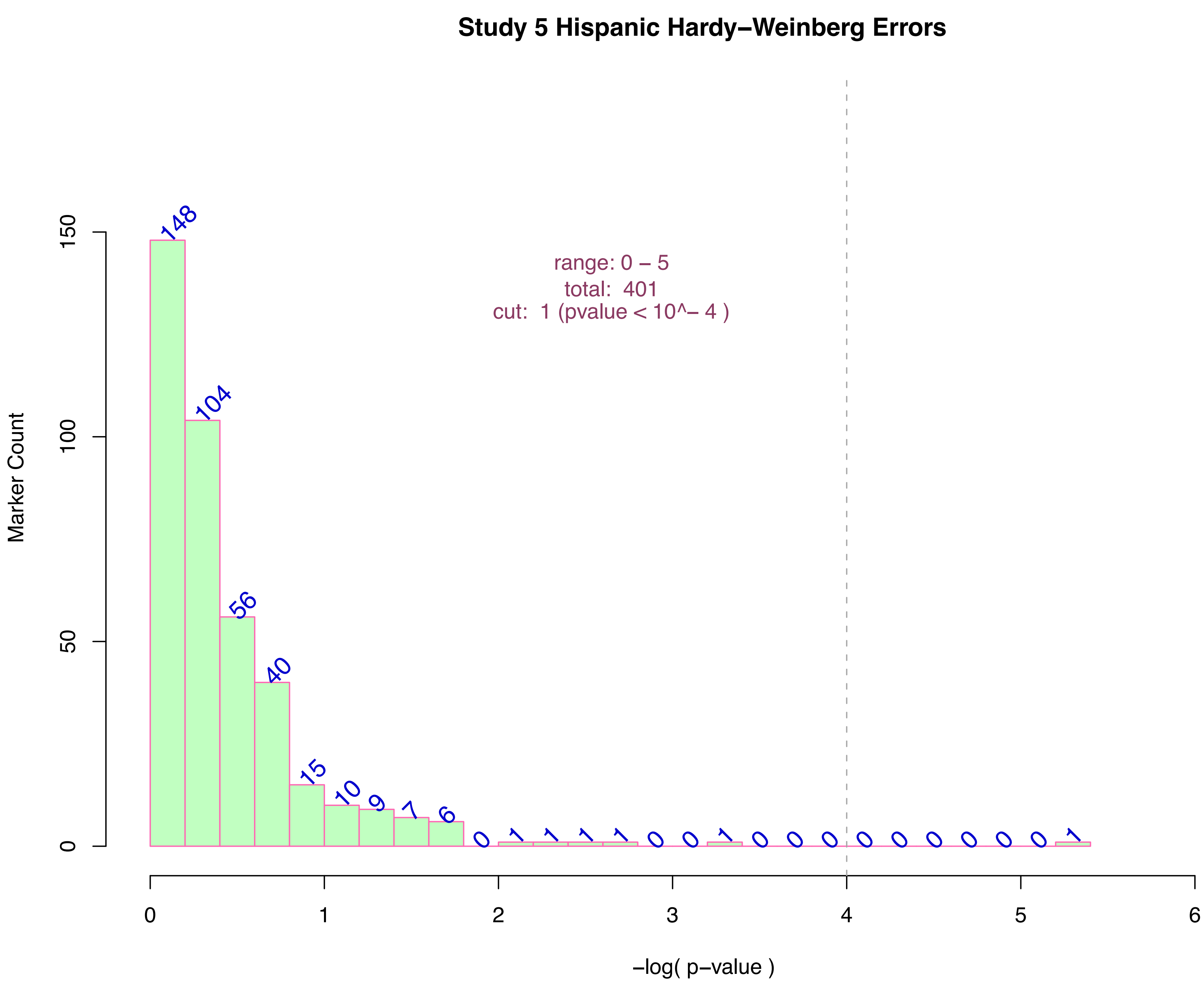
Study 5 Hispanic Hardy−Weinberg Errors
range: 0 − 5
total: 401
cut: 1 (pvalue < 10^− 4)
Marker Count
−log(p−value)
0
50
100
150
0
1
2
3
4
5
6
148
104
56
40
15
10
9
7
6
0
1
1
1
1
0
0
1
0
0
0
0
0
0
0
0
0
1

Study 5 Hispanic Initial Mendel Errors

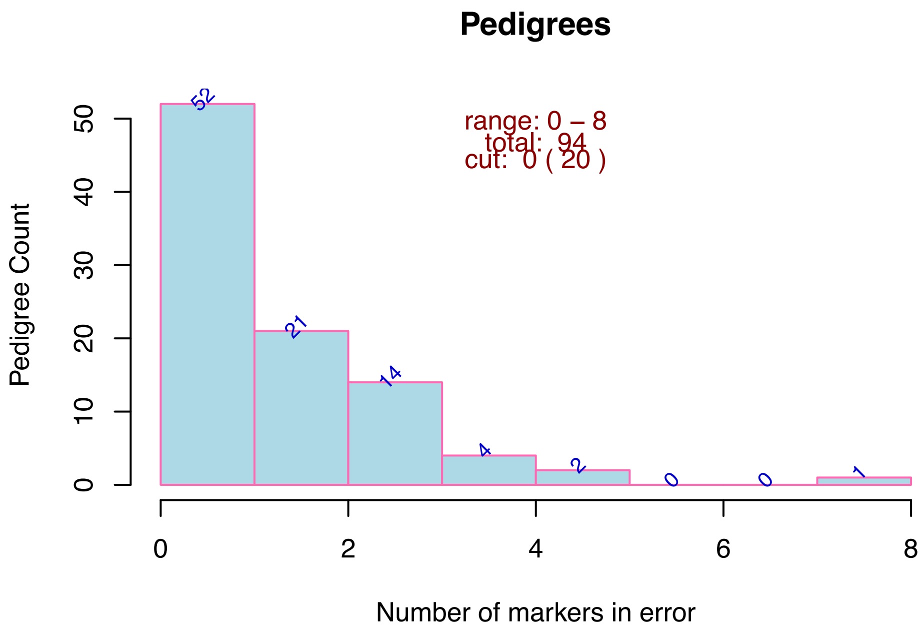

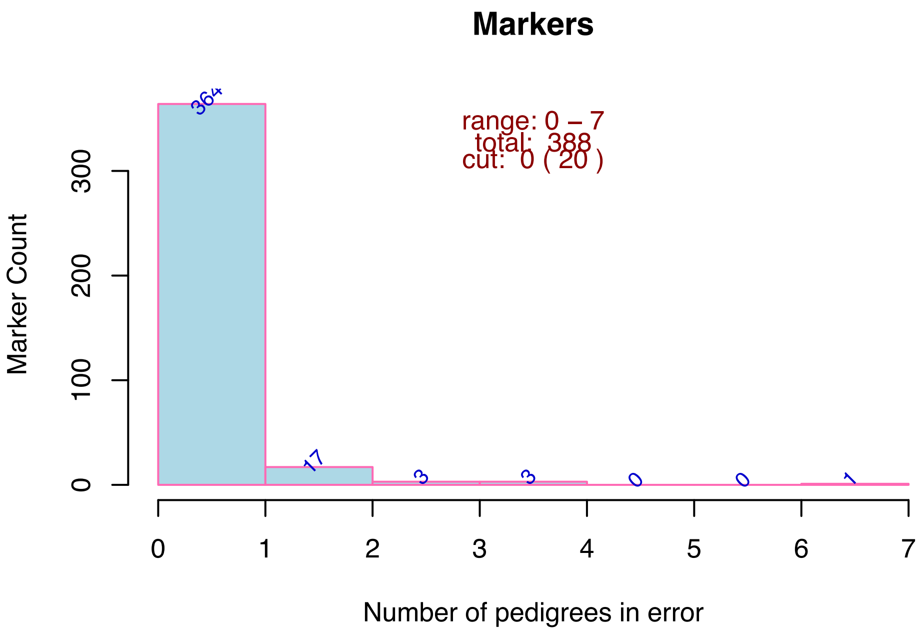

Study 5 Hispanic Initial Missingness

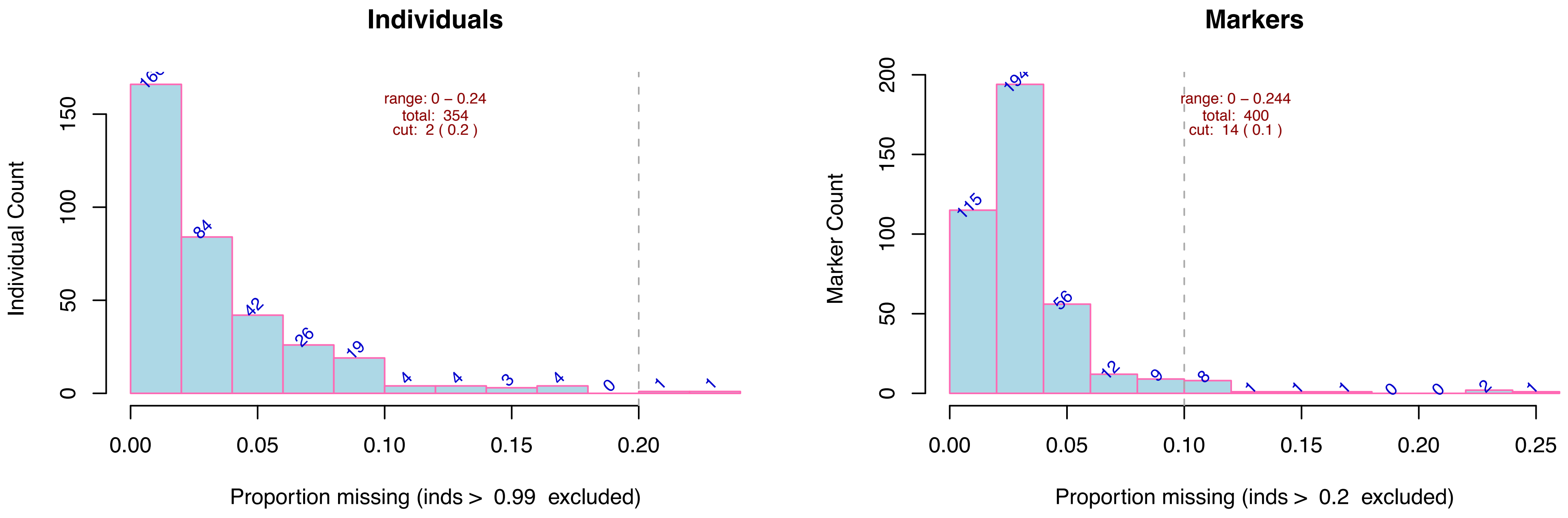

Study 5 Hispanic Repeat Missingness

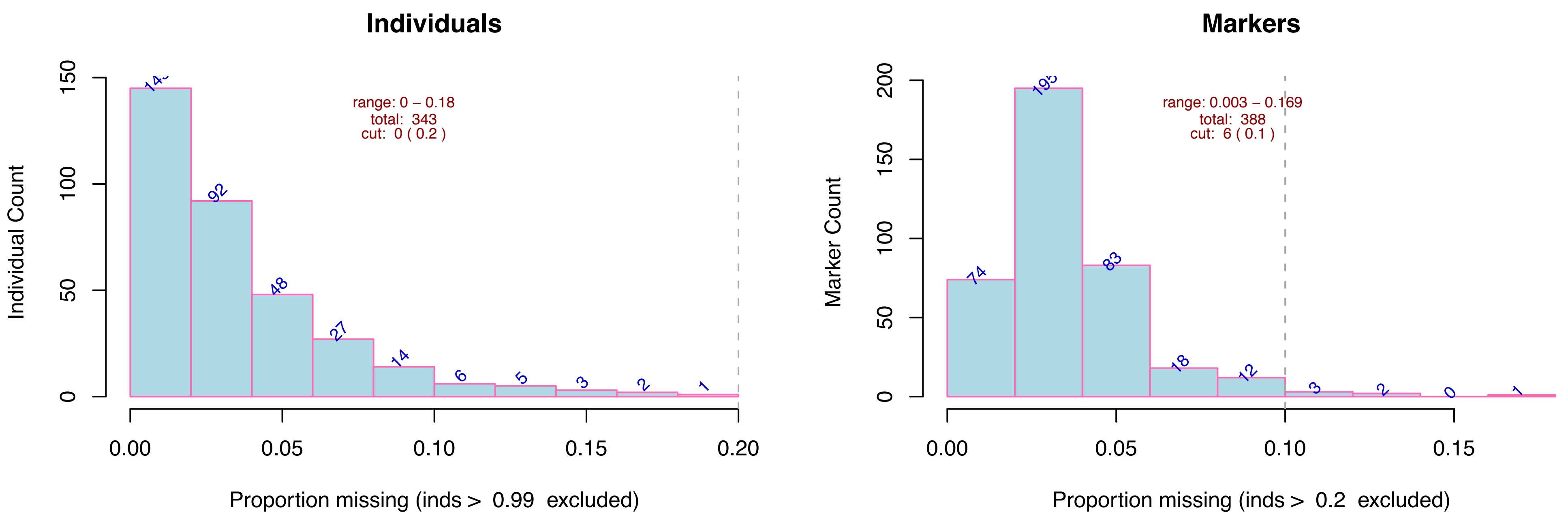

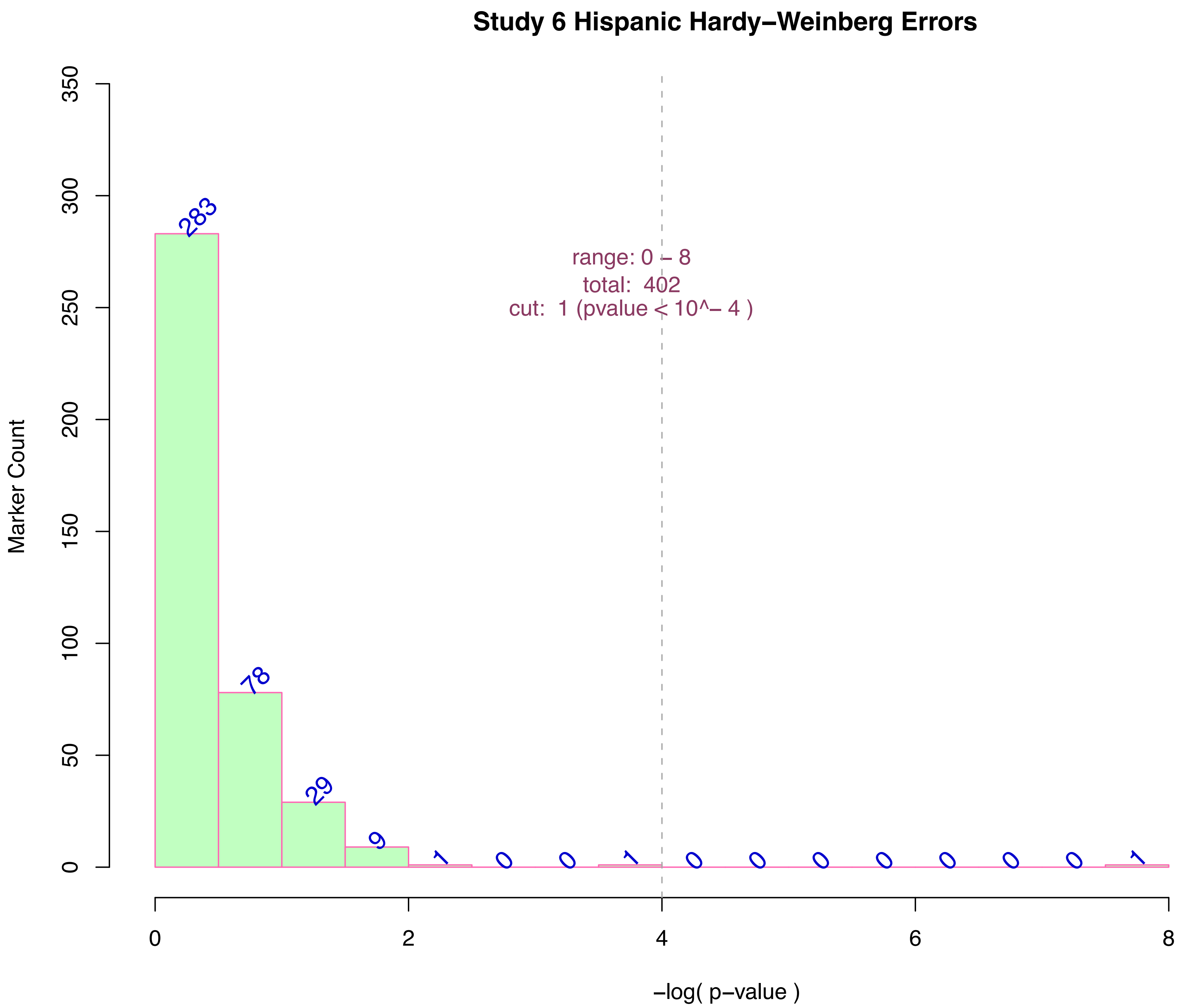
Study 6 Hispanic Hardy−Weinberg Errors
Marker Count
0
50
100
150
200
250
300
350
283
78
29
9
1
0
0
1
0
0
0
0
0
0
0
1
range: 0 − 8
total: 402
cut: 1 (pvalue < 10^− 4)
0
2
4
6
8
−log(p−value)

Study 6 Hispanic Initial Mendel Errors

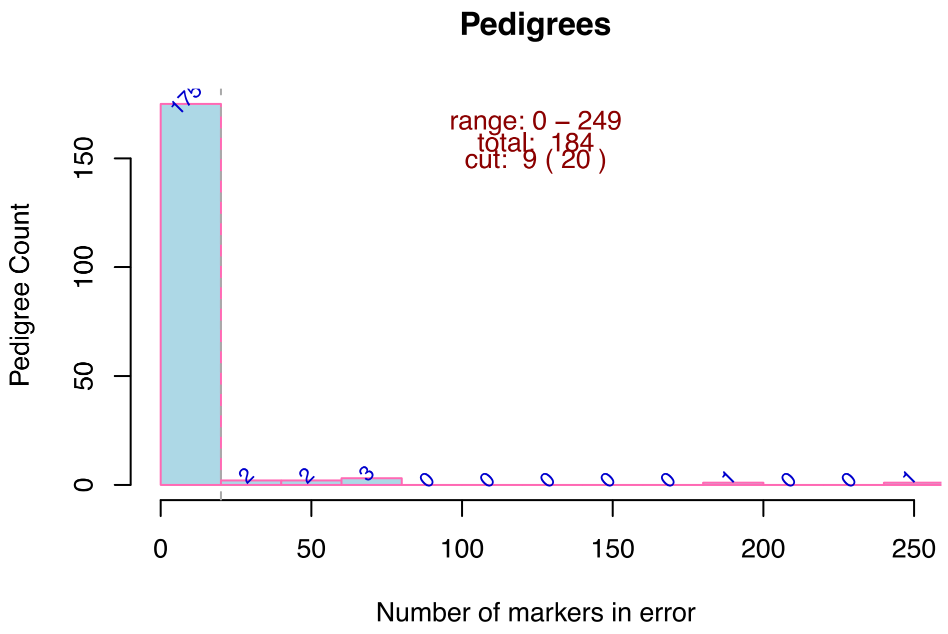

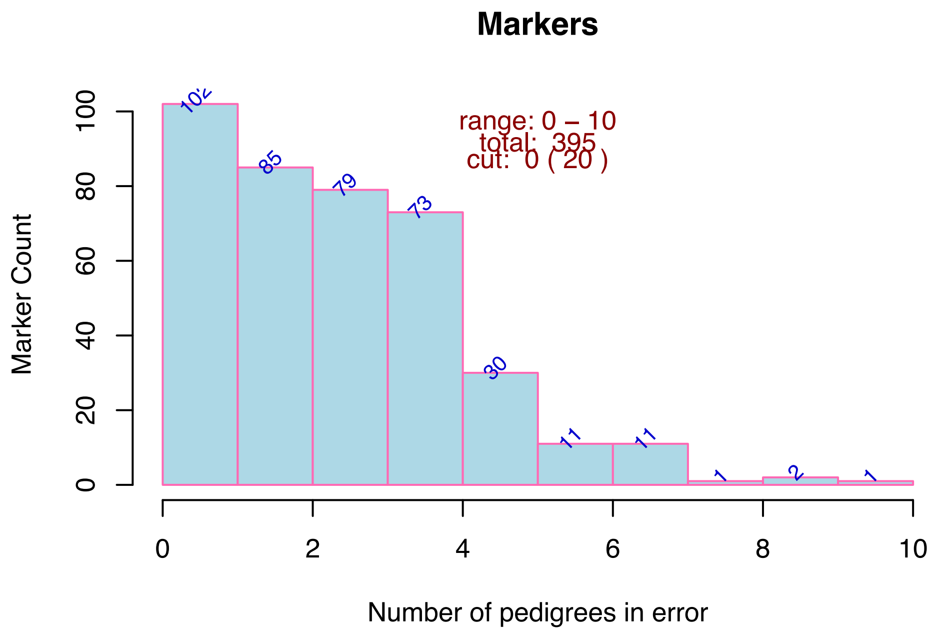

Study 6 Hispanic Initial Missingness

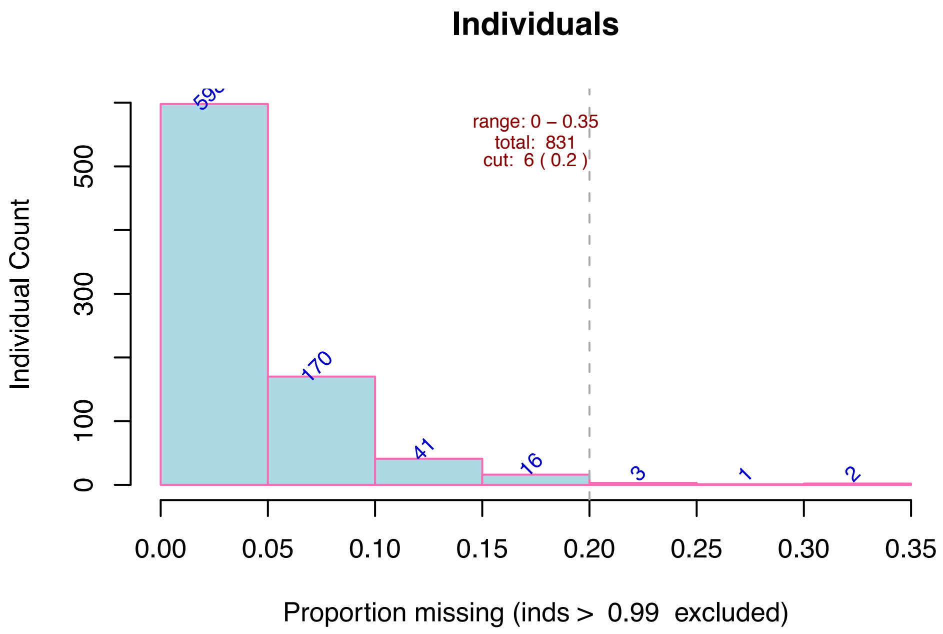

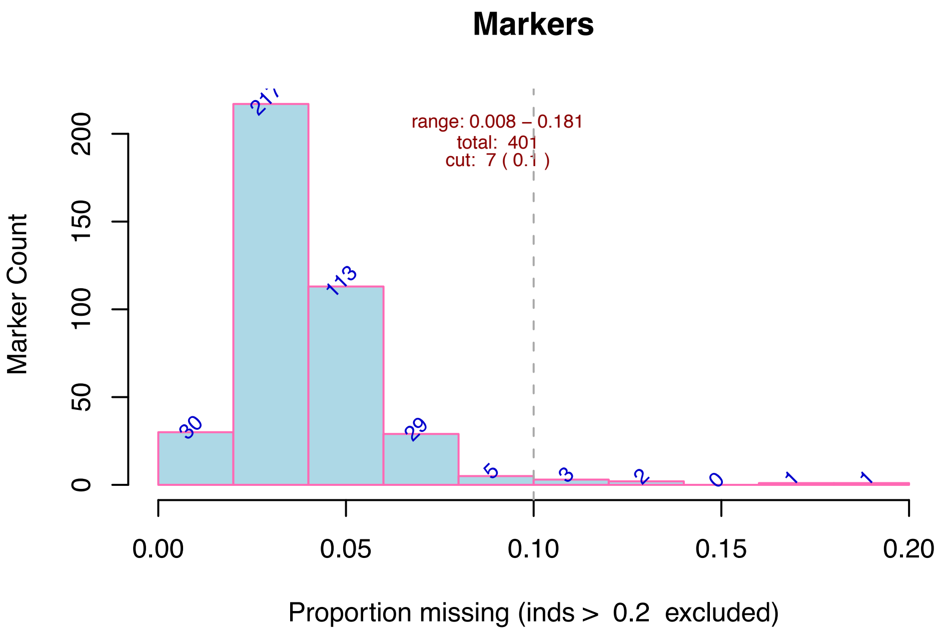

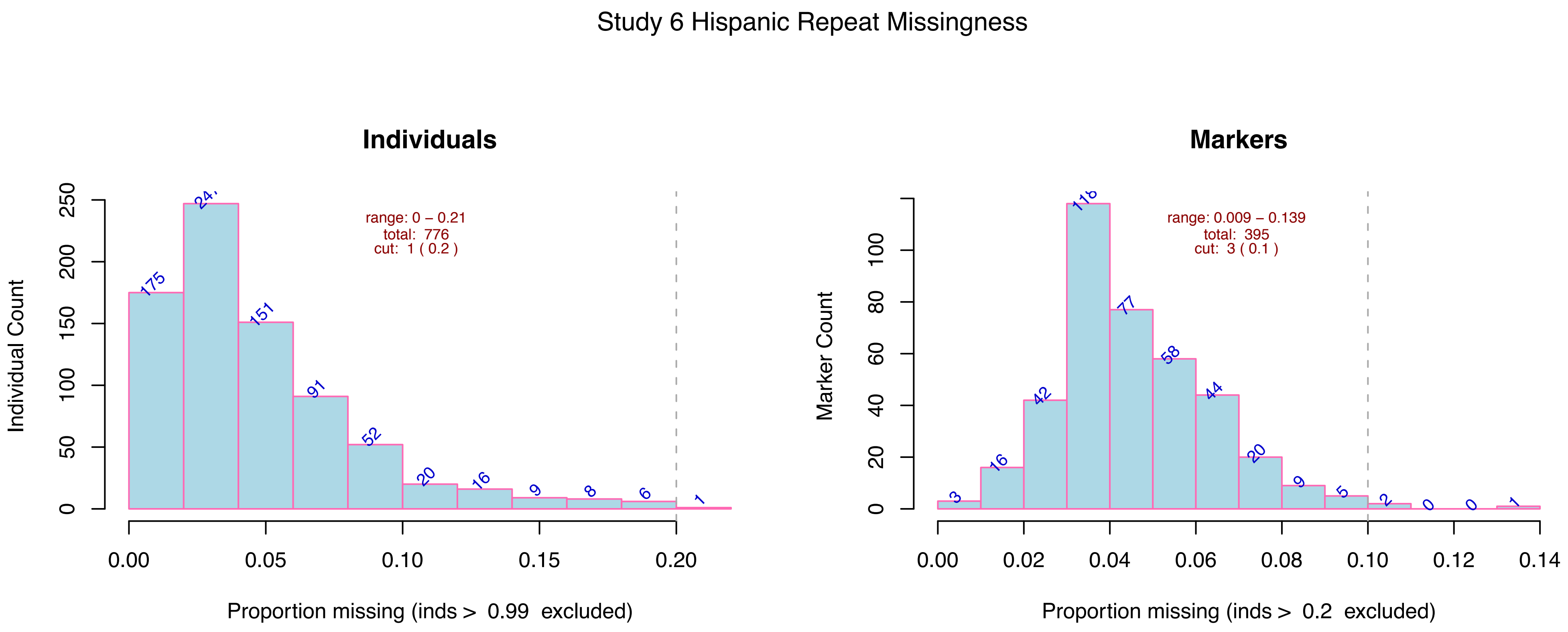

Study 6 Hispanic Repeat Missingness
Individuals
range: 0 – 0.21
total: 776
cut: 1 (0.2)
Individual Count
Proportion missing (inds > 0.99 excluded)
0
50
100
150
200
250
0.00
0.05
0.10
0.15
0.20
175
151
91
52
20
16
9
8
6
1
Markers
range: 0.009 – 0.139
total: 395
cut: 3 (0.1)
Marker Count
Proportion missing (inds > 0.2 excluded)
0
20
40
60
80
100
0.00
0.02
0.04
0.06
0.08
0.10
0.12
0.14
3
16
42
77
58
44
20
9
5
2
0
0
1

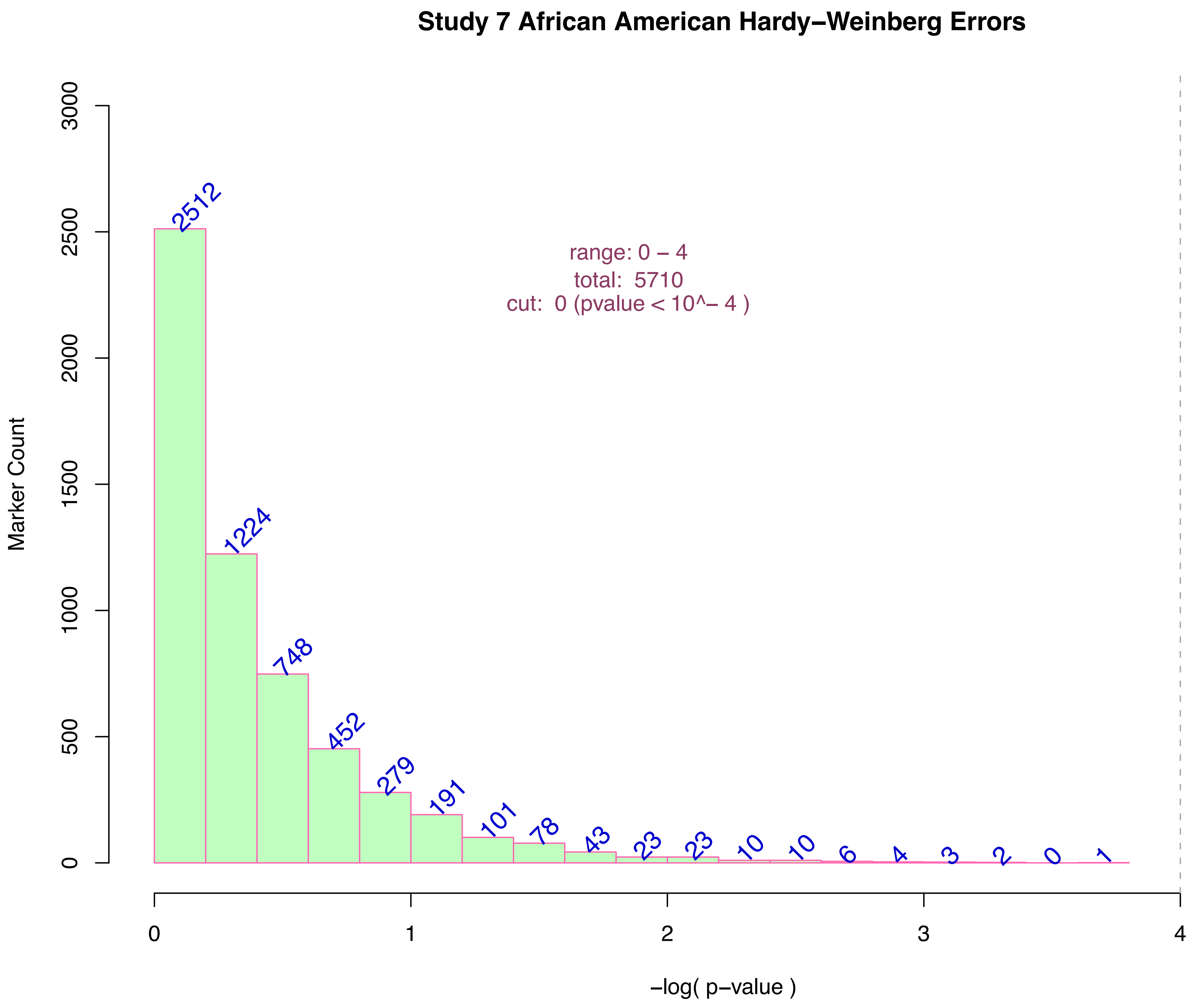
Study 7 African American Hardy−Weinberg Errors
range: 0 − 4
total: 5710
cut: 0 (pvalue < 10^− 4)
Marker Count
−log(p−value)
0
500
1000
1500
2000
2500
3000
0
1
2
3
4
2512
1224
748
452
279
191
101
78
43
23
23
10
10
6
4
3
2
0
1

Study 7 African American Initial Mendel Errors

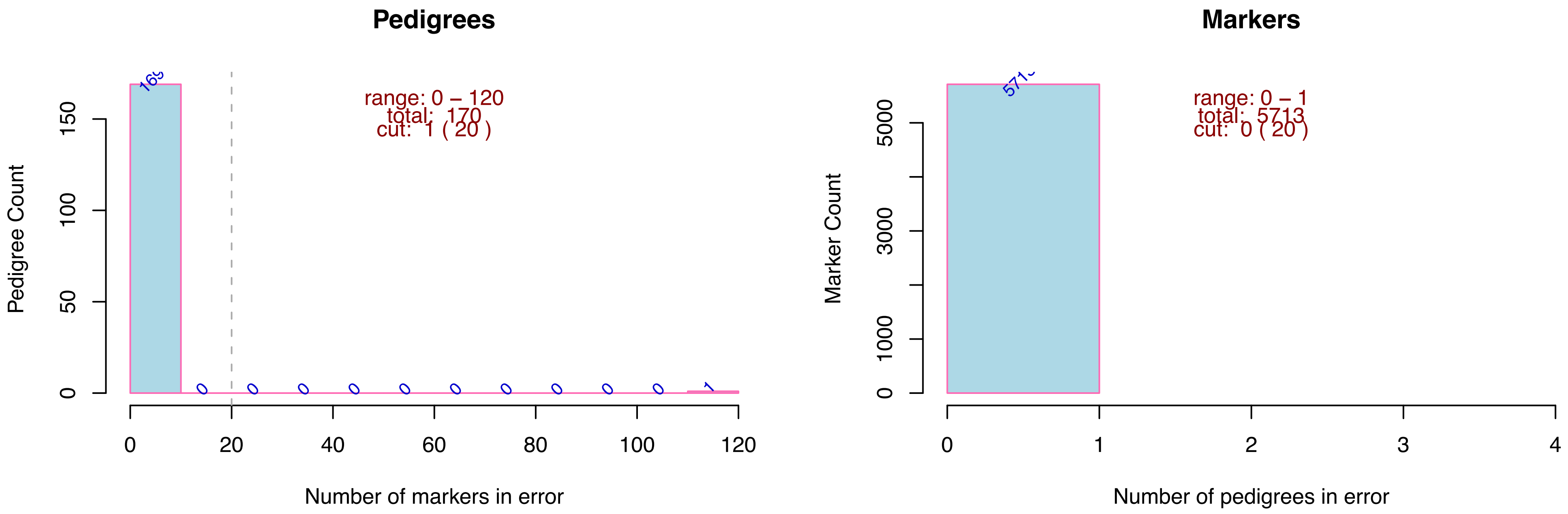

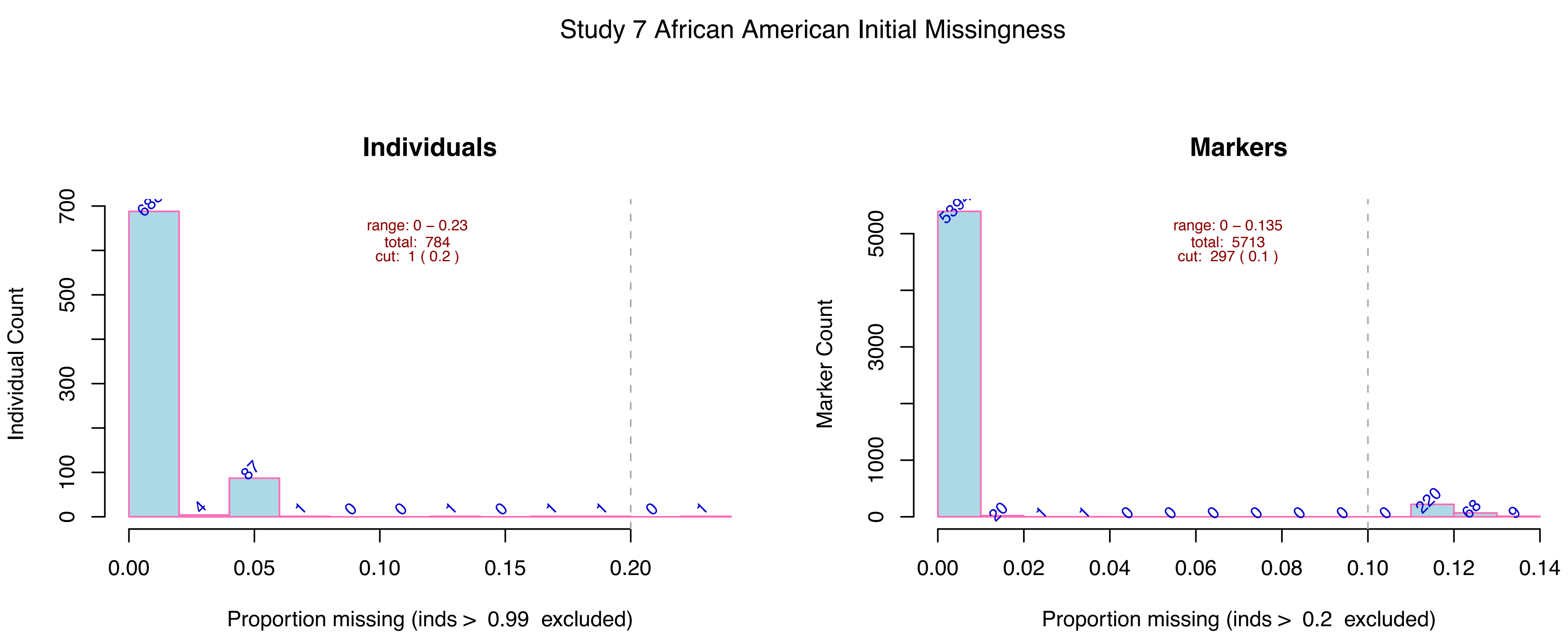
Study 7 African American Initial Missingness
Individuals
Individual Count
Proportion missing (inds > 0.99 excluded)
range: 0 – 0.23
total: 784
cut: 1 (0.2)
0
100
300
500
700
0.00
0.05
0.10
0.15
0.20
4
87
1
0
0
1
0
1
1
0
1
Markers
Marker Count
Proportion missing (inds > 0.2 excluded)
range: 0 – 0.135
total: 5713
cut: 297 (0.1)
0
1000
3000
5000
0.00
0.02
0.04
0.06
0.08
0.10
0.12
0.14
20
1
1
0
0
0
0
0
0
0
220
68
9

Study 7 African American Repeat Missingness

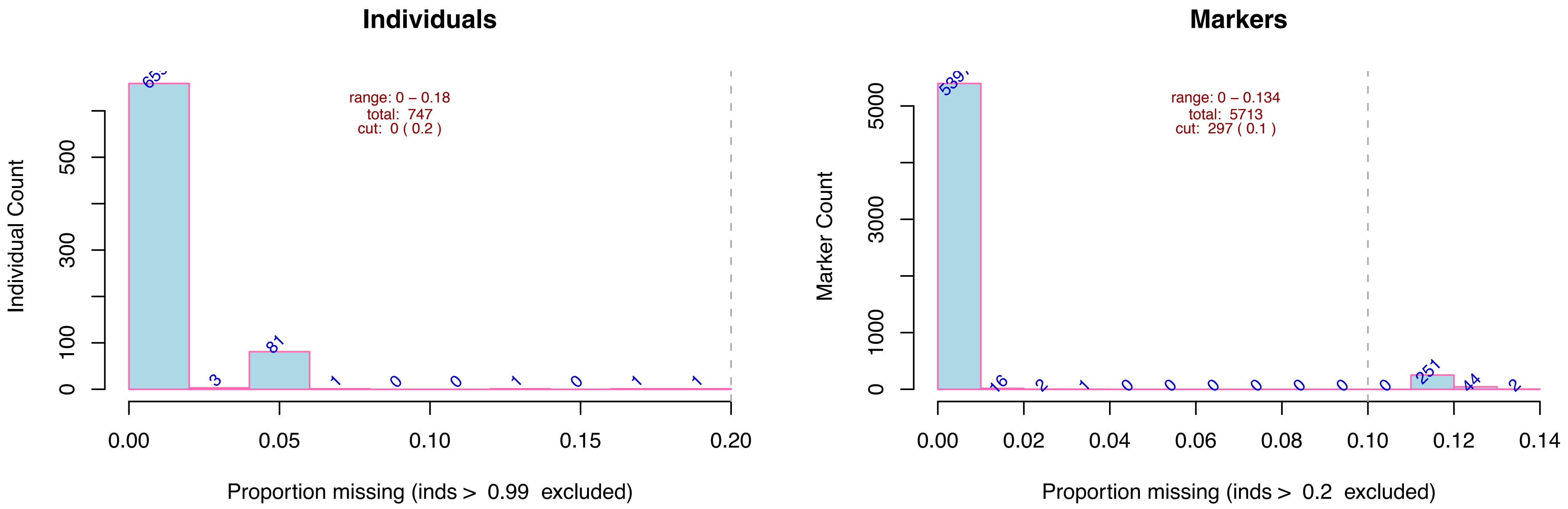

Supplement: Appendix S1 — Complete set of genotypic data processing histograms. The histograms included for each study are Hardy-Weinberg p-values, initial and repeat missingness proportions for markers and individuals, and Mendel error counts by family and by marker. The number of observations, the range in values, and the number of observations over threshold are annotated within each histogram. Note that the range of the x-axis is data dependent and that the vertical dashed line indicates the threshold (shown when data range exceeds it). (PDF) [file pone.0084696.s001.pdf]
