# Supplementary material for: Meta-Analysis of Repository Data: Impact of Data Regularization on NIMH Schizophrenia Linkage Results
Source: PLoS One. 2014 Jan 14;9(1):e84696. doi: 10.1371/journal.pone.0084696 (PMC3891773; doi:10.1371/journal.pone.0084696)
Supplement: Appendix S2 — Phenotypic data processing alogorithm including DSM codes. The diagnostic algorithm converts sets of DSM codes into three working variables (SZ, SA, BS), which are in turn translated into affectedness status in the table provided. For both versions of the codes, DSM-IIIR and DSM-IV, the code sets used in the algorithm are then itemized; they are: SZ_CODES, SA_CODES, BS_CODES, GLOBAL_EXCLUDE, UNAFF_EXCLUDE, SZ_DEMOTE1, SZ_DEMOTE2, SA_EXCLUDE, SA_DEMOTE, BS_EXCLUDE. (PDF) [file pone.0084696.s002.pdf]

Phenotype Processing Algorithm

1. Each individual has three associated variables which will be used for diagnostic classification. The variables are SZ, SA, and BS, which correspond to affection status for Schizophrenia, Schizoaffective Disorder, and Broad Spectrum. Each variable can have the value 0 (phenotype unknown for that diagnosis), 1 (not affected with that diagnosis), or 2 (affected with that diagnosis).
2. Initialize all three variables = 1
3. If person has any code in the SZ_CODES set, set SZ = 2
4. If person has any code in the SA_CODES set, set SA = 2
5. If person has any code in the BS_CODES set, set BS = 2
6. If person has no codes, set SZ=SA=BS = 0; done with this person
7. If person has any code in the GLOBAL_EXCLUDE set, set SZ=SA=BS=0; done with this person
8. If SZ=SA=BS=1:
    a. If person has any code in the UNAFF_EXCLUDE set, set SZ=SA=BS=0
    b. Done with this person
9. If SZ=2:
    a. If person has any code in the SZ_EXCLUDE set, set SZ=0
    b. If person has any code in the SZ_DEMOTE1 set, set SZ=0 and SA=2
    c. If person has any code in the SZ_DEMOTE2 set, set SZ=0, SA=0, and BS=2
10. If SA=2:
    a. If person has any code in the SA_EXCLUDE set, set SA=0
    b. If person has any code in the SA_DEMOTE set, set SA=0 and BS=2
11. If BS=2:
    a. If person has any code in the BS_EXCLUDE set, set BS=0
12. If SZ=2 AND SA=2, set SZ=0

Possible end states:

SZ	SA	BS	Classification	Diagnosis	Affection
2	[0,1]	[0,1,2]	Schizophrenia	4	Affected
[0,1]	2	[0,1,2]	Schizoaffective	3	Affected
[0,1]	[0,1]	2	Broad Spectrum	2	Unknown
1	1	1	Unaffected	1	Unaffected
0	0	0	Unknown	0	Unknown

DSMIIIR

[SZ_CODES]
295.10 Schizophrenia, disorganized type, unspecified
295.12 Schizophrenia, disorganized type, chronic
295.22 Schizophrenia, catatonic type, chronic
295.30 Schizophrenia, paranoid type, unspecified
295.32 Schizophrenia, paranoid type, chronic
295.60 Schizophrenia, residual type, unspecified
295.62 Schizophrenia, residual type, chronic
295.90 Schizophrenia, undifferentiated type, unspecified
295.92 Schizophrenia, undifferentiated type, chronic
295.34 Schizophrenia, paranoid type, chronic with acute exacerbation
295.35 Schizophrenia, paranoid type, in remission

[SA_CODES]
295.70 Schizoaffective disorder
295.70D Schizoaffective disorder, depression type
295.70M Schizoaffective disorder, manic type

[BS_CODES]
295.40 Schizophreniform disorder
297.10 Delusional disorder
298.90 Psychotic disorder NOS
301.00 Paranoid personality disorder
301.20 Schizoid personality disorder
301.22 Schizotypal personality disorder

[GLOBAL_EXCLUDE]
60   Dx Unknown
61   Un Dx Psychosis
62   Un Dx Psychosis, w/ hospitalization
63   Un Dx Psychiatric Disorder w/o hospitalization
290.10 Presenile dementia NOS OR Primary degenerative dementia of the Alzheimer type, presenile onset, uncomplicated
290.40 Multi-infarct dementia, uncomplicated
291.20 Dementia associated with alcoholism
292.12 Other or unspecified psychoactive substance hallucinosis
293.00 Delirium (etiology noted on Axis III or is unknown)
293.81 Organic delusional disorder (etiology noted on Axis III or is unknown)
293.82 Organic hallucinosis (etiology noted on Axis III or is unknown)
293.83 Organic mood disorder (etiology noted on Axis III or is unknown)

294.10 Dementia (etiology noted on Axis III or is unknown)
296.20 Major depression, single episode, unspecified
296.24 Major depression, single episode, with psychotic features
296.30 Major depression, recurrent, unspecified
296.34 Major depression, recurrent, with psychotic features
296.40 Bipolar disorder, manic, unspecified
296.44 Bipolar disorder, manic, with psychotic features
296.60 Bipolar disorder, mixed, unspecified
296.64 Bipolar disorder, mixed, with psychotic features
299.80 Pervasive developmental disorder NOS
300.90 Unspecified mental disorder (nonpsychotic)
304.00 Opioid dependence
304.10 Sedative, hypnotic, or anxiolytic dependence
304.20 Cocaine dependence
304.40 Amphetamine or similarly acting sympathomimetic dependence
304.50 Hallucinogen dependence OR Phencyclidine (PCP) or similarly acting arylcyclohexylamine dependence
304.60 Inhalant dependence
304.90 Polysubstance dependence OR Psychoactive substance dependence NOS
305.30 Hallucinogen abuse OR Hallucinogen hallucinosis
305.40 Sedative, hypnotic, or anxiolytic abuse OR Sedative, hypnotic, or anxiolytic intoxication
305.50 Opioid abuse OR Opioid intoxication
305.60 Cocaine abuse OR Cocaine intoxication
305.70 Amphetamine or similarly acting sympathomimetic abuse OR Amphetamine or similarly acting sympathomimetic intoxication
310.10 Organic personality disorder (etiology noted on Axis III or is unknown)
317.00 Mild mental retardation
318.00 Moderate mental retardation
319.00 Unspecified mental retardation
555.00 Any other diagnosis
290.00A Senile dementia NOS
290.00B Primary degenerative dementia of the Alzheimer type, senile onset, uncomplicated
292.00F Amphetamine or similarly acting sympathomimetic withdrawal
292.11C Other or unspecified psychoactive substance delusional disorder
292.11D Hallucinogen delusional disorder
292.84A Hallucinogen mood disorder
292.84B Other or unspecified psychoactive substance mood disorder
294.80A Organic mental disorder NOS (etiology noted on Axis III or is unknown)
296.24C Major depression, single episode, with psychotic features, mood congruent
296.24I Major depression, single episode, with psychotic features, mood incongruent
296.34C Major depression, recurrent, with psychotic features, mood congruent
296.34I Major depression, recurrent, with psychotic features, mood incongruent
296.44C Bipolar disorder, manic, with psychotic features, mood congruent
296.44I Bipolar disorder, manic, with psychotic features, mood incongruent
304.50A Hallucinogen dependence
304.50B Phencyclidine (PCP) or similarly acting arylcyclohexylamine dependence

304.90A Polysubstance dependence
304.90B Psychoactive substance dependence NOS
305.30A Hallucinogen hallucinosis
305.30B Hallucinogen Abuse/Dep.
305.40A Sedative, hypnotic, or axiolytic abuse
305.50A Opiod abuse
305.50B Opioid intoxication
305.60A Cocaine abuse
305.70B Amphetamine or similarly acting sympathomimetic abuse
305.90A Phencyclidine (PCP) or similarly acting arlycyclohexylamine abuse
305.90C Inhalant abuse
305.90D Psychoactive substance abuse NOS
305.90E Other or unspecified psychoactive substance intoxication
305.90G Inhalant intoxication
315.90A Development disorder NOS
799.90B Diagnosis or condition deferred on Axis I
V40.00 Borderline intellectual functioning

[UNAFF_EXCLUDE]
296.89 Bipolar II Disorder
296.90 Mood Disorder NOS
301.90 Personality disorder NOS
311.00 Depressive disorder NOS
296.70A Bipolar disorder, NOS
799.90A Diagnosis or condition deferred on Axis II

[SZ_EXCLUDE]

[SZ_DEMOTE1]
296.31 Major depression, recurrent, mild
296.32 Major depression, recurrent, moderate
296.33 Major depression, recurrent, severe, without psychotic features
296.35 Major depression, recurrent, in partial remission
296.36 Major depression, recurrent, in full remission
296.52 Bipolar disorder, depressed, moderate
296.55 Bipolar disorder, depressed, in partial remission
296.89 Bipolar II Disorder
296.90 Mood Disorder NOS
311.00 Depressive disorder NOS
296.70A Bipolar disorder, NOS

[SZ_DEMOTE2]

[SA_EXCLUDE]

[SA_DEMOTE]

[BS_EXCLUDE]

DSMIV

[SZ_CODES]
295.00 Schizophrenia, unspecified
295.10 Schizophrenia, Disorganized Type
295.20 Schizophrenia, Catatonic Type
295.30 Schizophrenia, Paranoid Type
295.60 Schizophrenia, Residual Type
295.90 Schizophrenia, Undifferentiated Type
295.92 Schizophrenia, undifferentiated type, chronic

[SA_CODES]
295.70 Schizoaffective Disorder
295.70D Schizoaffective disorder, depressive type
295.70M Schizoaffective disorder, bipolar type

[BS_CODES]
295.40 Schizophreniform Disorder
298.8   Brief Psychotic Disorder
298.9   Psychotic Disorder NOS
301.0   Paranoid Personality Disorder
301.20 Schizoid Personality Disorder
301.22 Schizotypal Personality Disorder
297.10/298   Delusional Disorder/Pychotic Disorder
297.1   Delusional Disorder

[GLOBAL_EXCLUDE]
290.0   Dementia of the Alzheimer's Type, With Late Onset, Uncomplicated (DSMIV)
291.2   Alcohol-Induced Persisting Dementia
291.3   Alcohol-Induced Psychotic Disorder, With Hallucinations
291.5   Alcohol-Induced Psychotic Disorder, With Delusions
291.9   Alcohol-Related Disorder NOS
292.11 Amphetamine-Induced Psychotic Disorder, With Delusions OR Cannabis-Induced Psychotic Disorder, With Delusions OR Cocaine-Induced Psychotic Disorder, With Delusions OR Hallucinogen-Induced Psychotic Disorder, With Delusions OR Inhalant-Induced Psychotic Disorder, With Delusions OR Opioid-Induced Psychotic Disorder, With Delusions OR Other (or Unknown) Substance-Induced Psychotic Disorder, With Delusions OR Phencyclidine-Induced Psychotic Disorder, With Delusions OR Sedative-, Hypnotic-, or Anxiolytic-Induced Psychotic Disorder, With Delusions
292.89 Amphetamine-Induced Anxiety Disorder OR Amphetamine-Induced Sexual Dysfunction OR Amphetamine-Induced Sleep Disorder OR Amphetamine Intoxication OR Caffeine-Induced Anxiety Disorder OR Caffeine-Induced Sleep Disorder OR Cannabis-Induced Anxiety Disorder OR Cannabis Intoxication OR Cocaine-Induced Anxiety Disorder OR Cocaine-Induced Sexual Dysfunction OR Cocaine-Induced Sleep Disorder OR Cocaine

Intoxication OR Hallucinogen-Induced Anxiety Disorder OR Hallucinogen Intoxication OR Hallucinogen Persisting Perception Disorder OR Inhalant-Induced Anxiety Disorder OR Inhalant Intoxication OR Opioid-Induced Sexual Dysfunction OR Opioid-Induced Sleep Disorder OR Opioid Intoxication OR Other (or Unknown) Substance-Induced Anxiety Disorder OR Other (or Unknown) Substance-Induced Sexual Dysfunction OR Other (or Unknown) Substance-Induced Sleep Disorder OR Other (or Unknown) Substance Intoxication OR Phencyclidine-Induced Anxiety Disorder OR Phencyclidine Intoxication OR Sedative-, Hypnotic-, or Anxiolytic-Induced Anxiety Disorder OR Sedative-, Hypnotic-, or Anxiolytic-Induced Sexual Dysfunction OR Sedative-, Hypnotic-, or Anxiolytic-Induced Sleep Disorder OR Sedative, Hypnotic, or Anxiolytic Intoxication
293.81 Psychotic Disorder Due to ... [Indicate the General Medical Condition], With Delusions
293.82 Psychotic Disorder Due to ... [Indicate the General Medical Condition], With Hallucinations
293.83 Mood Disorder Due to ... [Indicate the General Medical Condition]
293.9   Mental Disorder NOS Due to ... [Indicate the General Medical Condition]
294.1   Dementia Due to ... [Indicate the General Medical Condition] (DSMIV)
294.8   Amnestic Disorder NOS OR Dementia NOS
296.00 Bipolar I Disorder, Single Manic Episode, Unspecified
296.04 Bipolar I Disorder, Single Manic Episode, Severe With Psychotic Features
296.20 Major Depressive Disorder, Single Episode, Unspecified
296.24 Major Depressive Disorder, Single Episode, Severe With Psychotic Features
296.30 Major Depressive Disorder, Recurrent, Unspecified
296.34 Major Depressive Disorder, Recurrent, Severe With Psychotic Features
296.44 Bipolar I Disorder, Most Recent Episode Manic, Severe With Psychotic Features
296.54 Bipolar I Disorder, Most Recent Episode Depressed, Severe With Psychotic Features
296.60 Bipolar I Disorder, Most Recent Episode Mixed, Unspecified
296.64 Bipolar I Disorder, Most Recent Episode Mixed, Severe With Psychotic Features
296.7   Bipolar I Disorder, Most Recent Episode Unspecified
300.9   Unspecified Mental Disorder (nonpsychotic)
304.00 Opioid Dependence
304.10 Sedative, Hypnotic, or Anxiolytic Dependence
304.20 Cocaine Dependence
304.40 Amphetamine Dependence
304.50 Hallucinogen Dependence
304.60 Inhalant Dependence (DSMIV) OR Inhalant Dependence OR Phencyclidine Dependence (DSMIVTR)
304.80 Polysubstance Dependence
305.30 Hallucinogen Abuse
305.40 Sedative, Hypnotic, or Anxiolytic Abuse
305.50 Opioid Abuse
305.60 Cocaine Abuse
305.70 Amphetamine Abuse
305.90 Caffeine Intoxication OR Inhalant Abuse OR Other (or Unknown) Substance Abuse OR Phencyclidine Abuse
315.9   Learning Disorder NOS
317   Mild Mental Retardation
318.0   Moderate Mental Retardation
318.1   Severe Mental Retardation
319   Mental Retardation, Severity Unspecified
555.00 Any other diagnosis

799.9   Diagnosis Deferred on Axis II OR Diagnosis or Condition Deferred on Axis I
290.10A Dementia due to Creutzfeldt-Jakob disease
290.10C Dementia of the Alzheimer's type, with early onset, uncomplicated
291.8B Alcohol-induced mood disorder
292.11A Amphetamine-induced psychotic disorder, with delusions
292.11G Other (or unknown) substance-induced psychotic disorder, with delusions
292.12A Amphetamine-induced psychotic disorder, with hallucinations
292.12B "Cannabis-induced psychotic disorder, with hallucinations"
292.12C Cocaine-induced psychotic disorder, with hallucinations
292.12G Other (or unknown) substance-induced psychotic disorder, with hallucinations
292.84B Cocaine-induced mood disorder
292.84F Other (or unknown) substance-induced mood disorder
292.9I Other (or unknown) substance-related disorder NOS
293.89B Catatonic disorder due to [Indicate the general medical condition]
294.8B Dementia NOS
294.9A Cognitive disorder NOS
294.9B Dementia due to HIV disease
296.34C Major Depressive Disorder, Recurrent, Severe With Psychotic Features, Mood Congruent
296.34I Major Depressive Disorder, Recurrent, Severe With Psychotic Features, Mood Incongruent
296.40A Bipolar I disorder, most recent episode hypomanic
296.40B Bipolar I disorder, most recent episode manic, unspecified
296.74C Bipolar I Disorder, Most Recent Episode Unspecified, Severe With Psychotic Features, Mood Congruent
296.74I Bipolar I Disorder, Most Recent Episode Unspecified, Severe With Psychotic Features, Mood Incongruent
299.80B Pervasive developmental disorder NOS
304.90A Other (or unknown) substance dependence
304.90B Phencyclidine dependence
304.xx/305   Other (or Unknown) Substance Dependence/Alcohol Abuse
305.90B Inhalant abuse
305.90C Other (or unknown) substance abuse
305.90D Phencyclidine abuse
799.9B Diagnosis or condition deferred on Axis I
V62.89 Borderline Intellectual Functioning OR Phase of Life Problem OR Religious or Spiritual Problem
V62.89A Borderline intellectual functioning

[UNAFF_EXCLUDE]
296.80 Bipolar Disorder NOS
296.89 Bipolar II Disorder
296.90 Mood Disorder NOS
301.9   Personality Disorder NOS
311   Depressive Disorder NOS
799.9A Diagnosis deferred on Axis II

[SZ_EXCLUDE]

[SZ_DEMOTE1]
296.02 Bipolar I Disorder, Single Manic Episode, Moderate
296.31 Major Depressive Disorder, Recurrent, Mild
296.32 Major Depressive Disorder, Recurrent, Moderate
296.33 Major Depressive Disorder, Recurrent, Severe Without Psychotic Features
296.35 Major Depressive Disorder, Recurrent, In Partial Remission
296.36 Major Depressive Disorder, Recurrent, In Full Remission
296.41 Bipolar I Disorder, Most Recent Episode Manic, Mild
296.46 Most Recent Episode Manic, In Full Remission
296.52 Bipolar I Disorder, Most Recent Episode Depressed, Moderate
296.55 Bipolar I Disorder, Most Recent Episode Depressed, In Partial Remission
296.56 Bipolar I Disorder, Most Recent Episode Depressed, In Full Remission
296.80 Bipolar Disorder NOS
296.89 Bipolar II Disorder
296.90 Mood Disorder NOS
311   Depressive Disorder NOS

[SZ_DEMOTE2]

[SA_EXCLUDE]

[SA_DEMOTE]

[BS_EXCLUDE]
