# Supplementary material for: Meta-Analysis of Repository Data: Impact of Data Regularization on NIMH Schizophrenia Linkage Results
Source: PLoS One. 2014 Jan 14;9(1):e84696. doi: 10.1371/journal.pone.0084696 (PMC3891773; doi:10.1371/journal.pone.0084696)

Study 1 African American

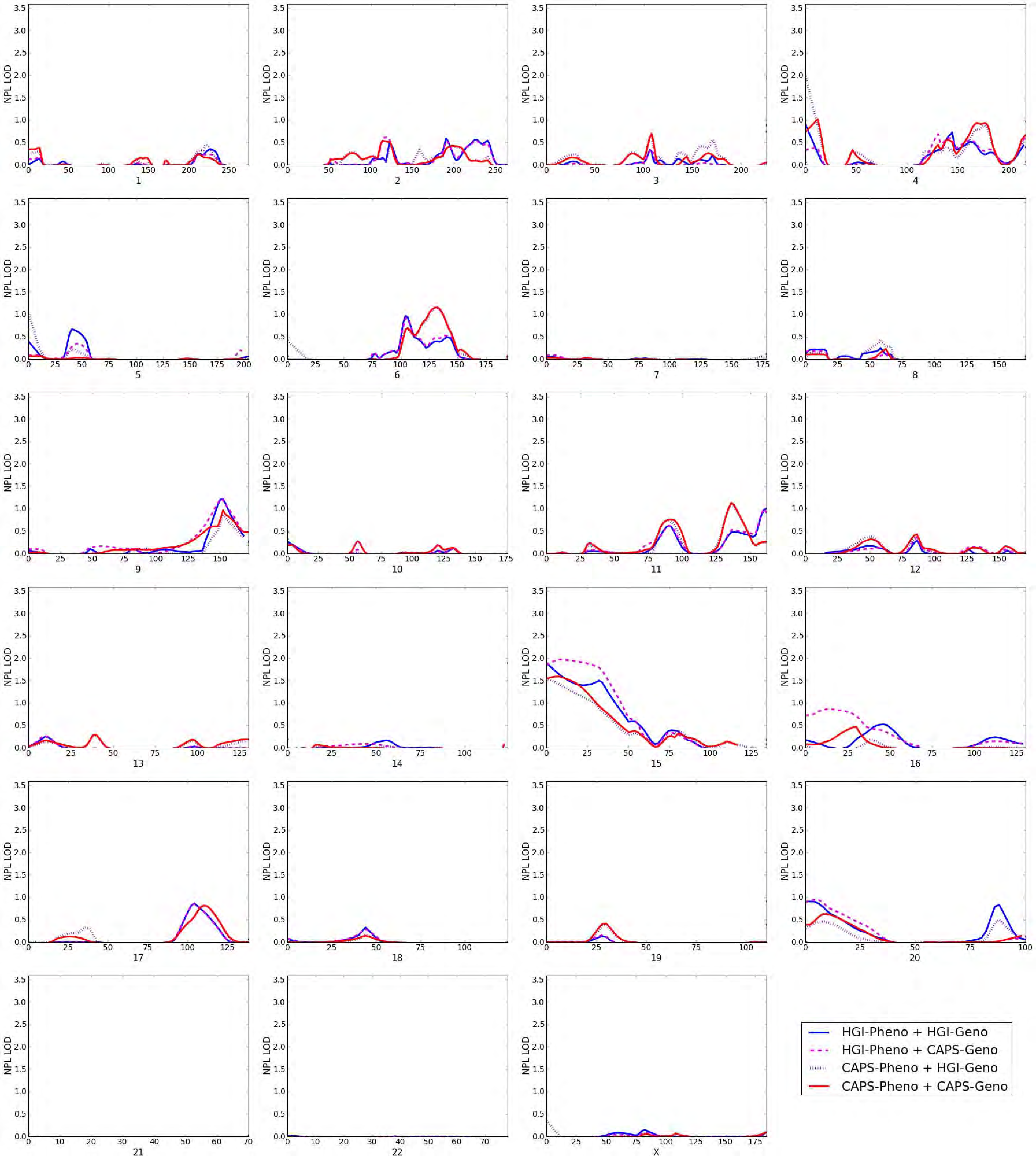

Study 1 European American

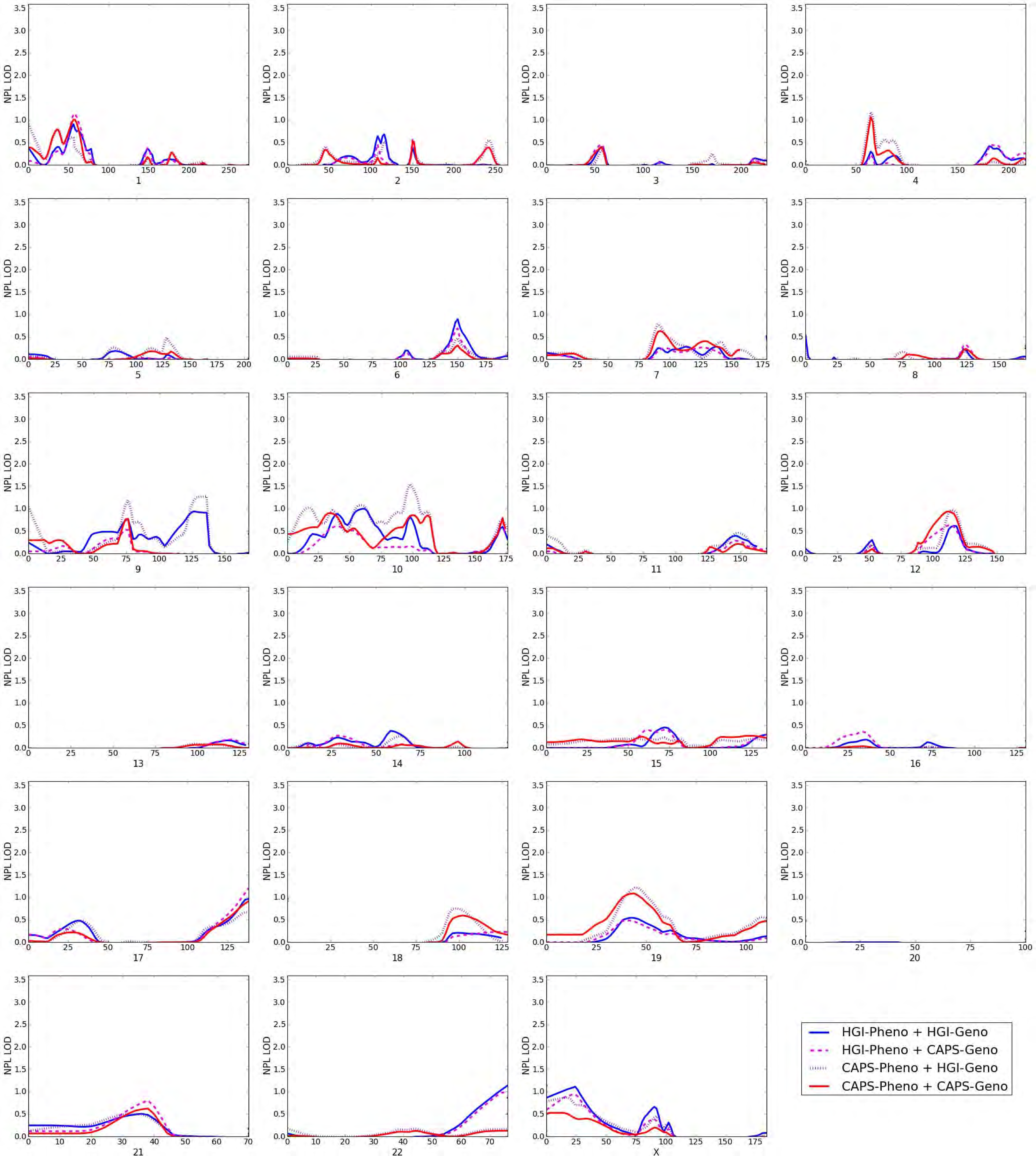

Study 2 African American

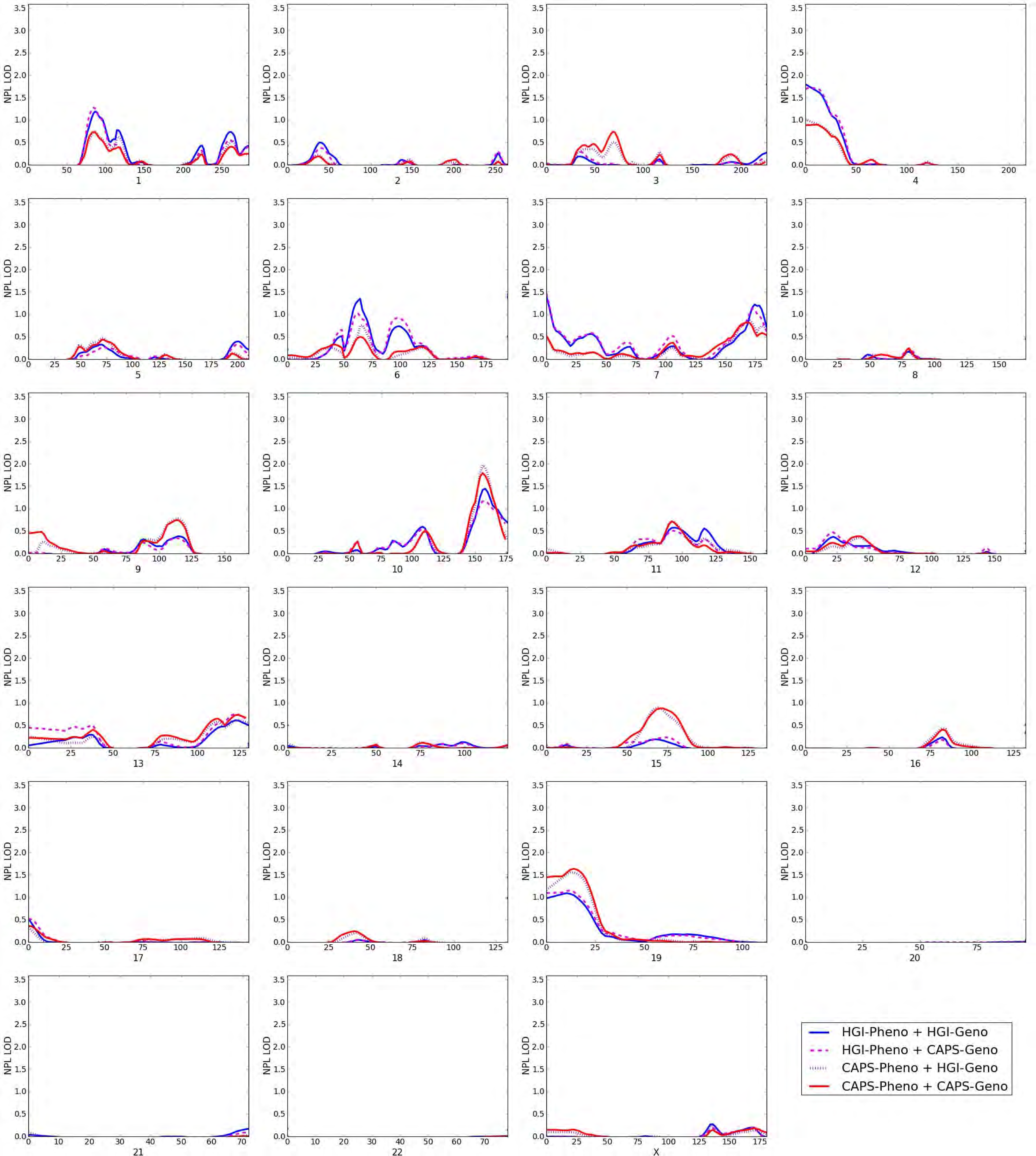

Study 2 European American

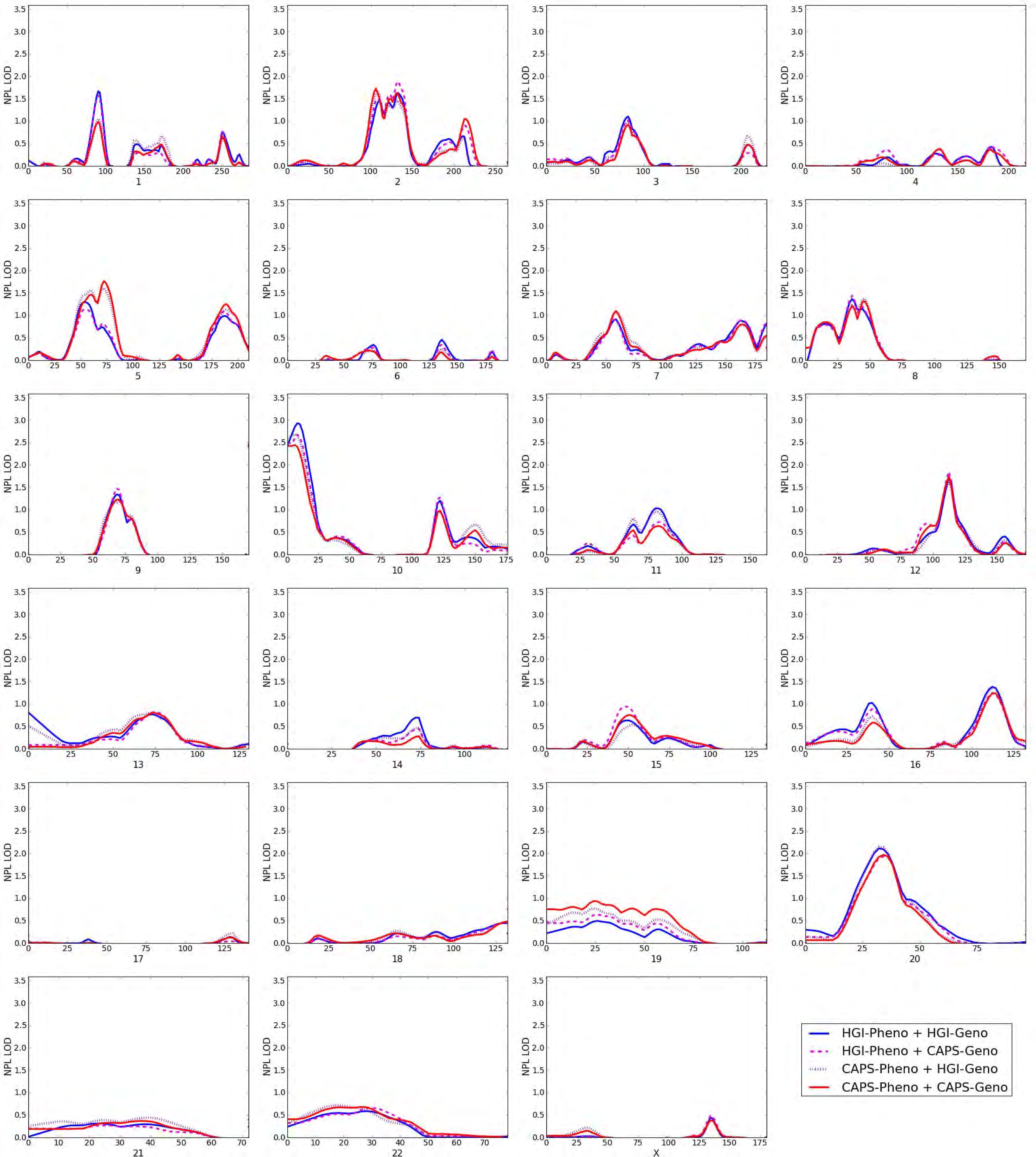

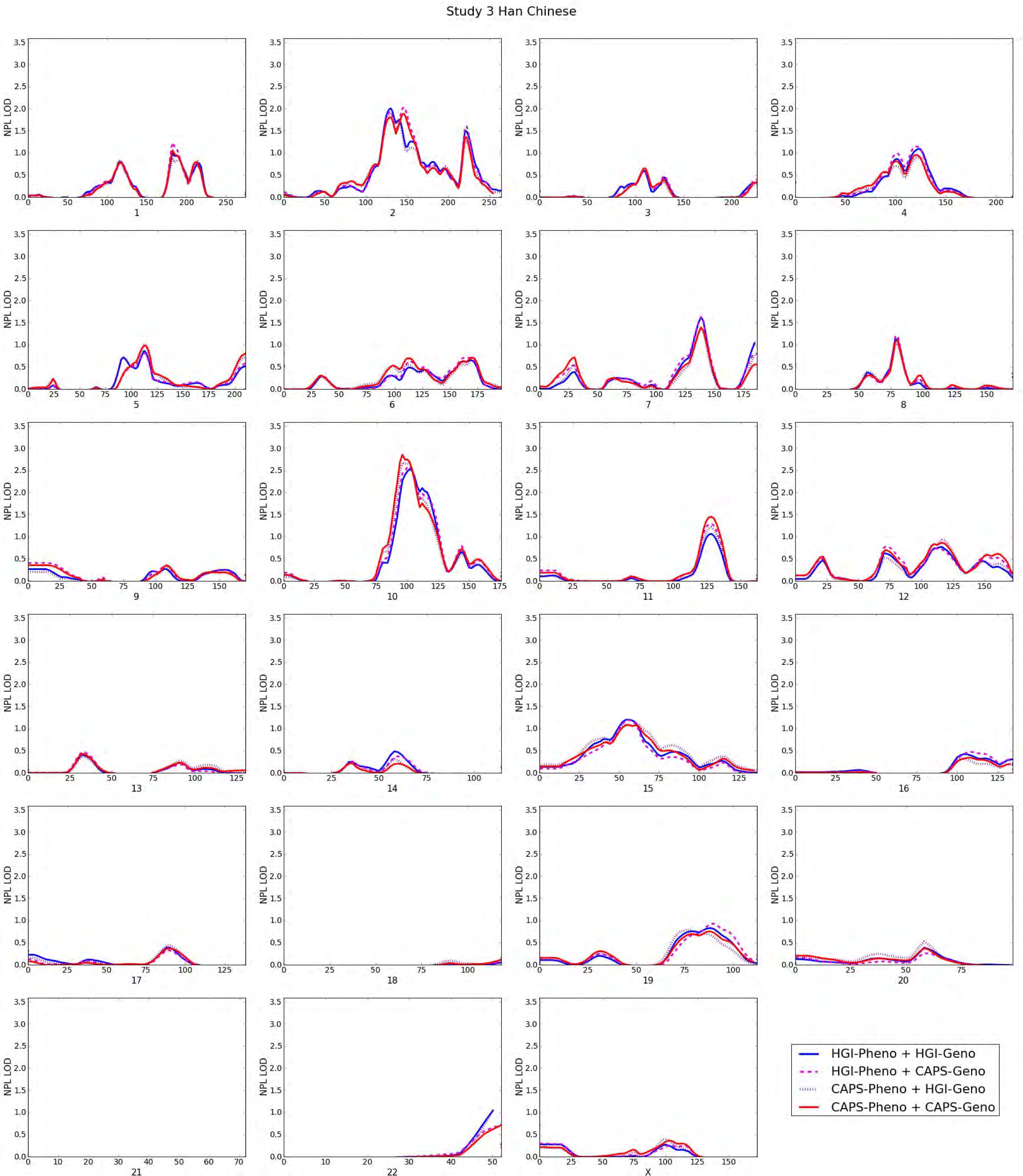

Study 3 Han Chinese
NPL LOD
1
2
3
4
5
6
7
8
9
10
11
12
13
14
15
16
17
18
19
20
21
22
X
HGI-Pheno + HGI-Geno
HGI-Pheno + CAPS-Geno
CAPS-Pheno + HGI-Geno
CAPS-Pheno + CAPS-Geno

Study 4 European American

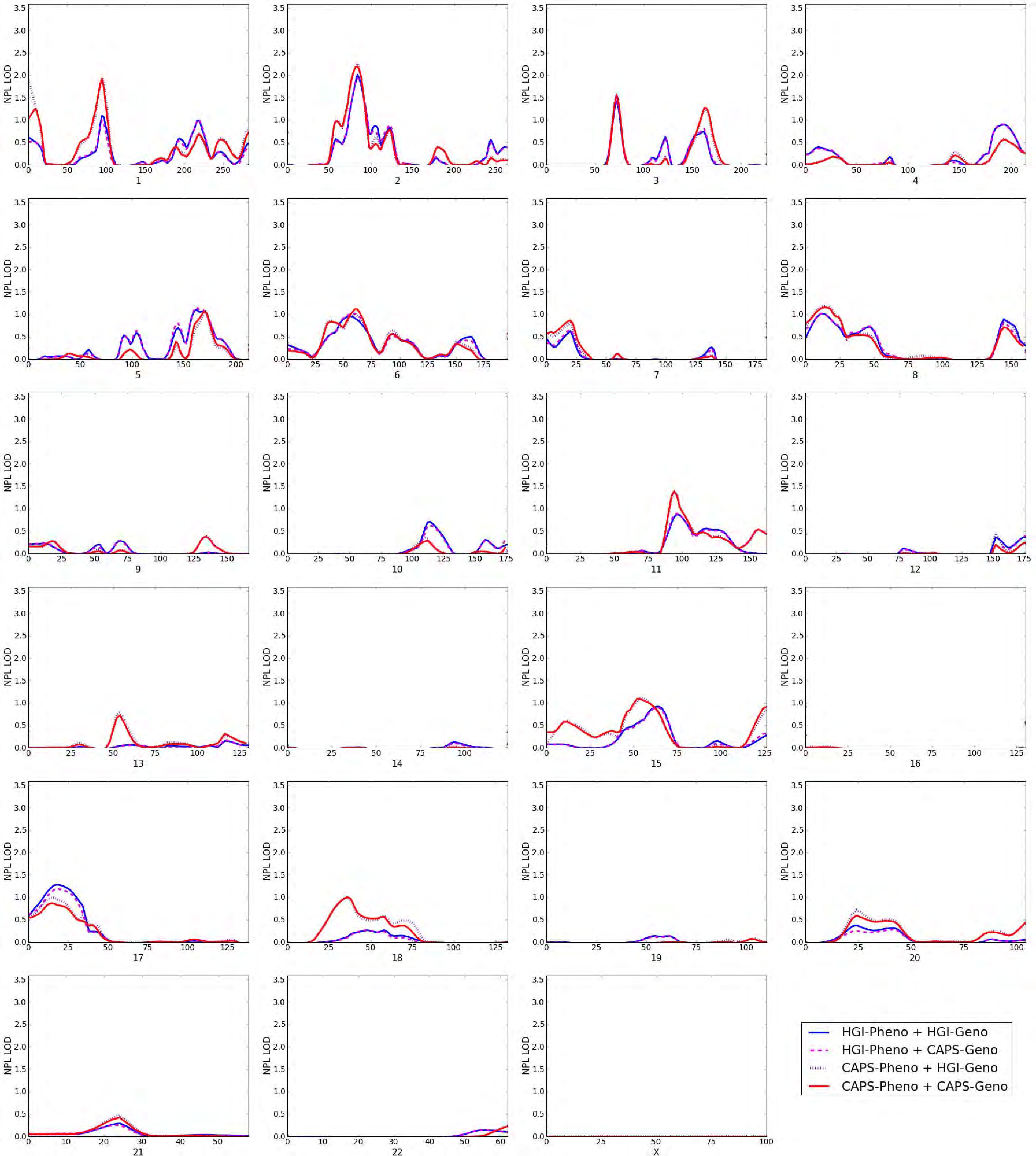

Study 5 Hispanic

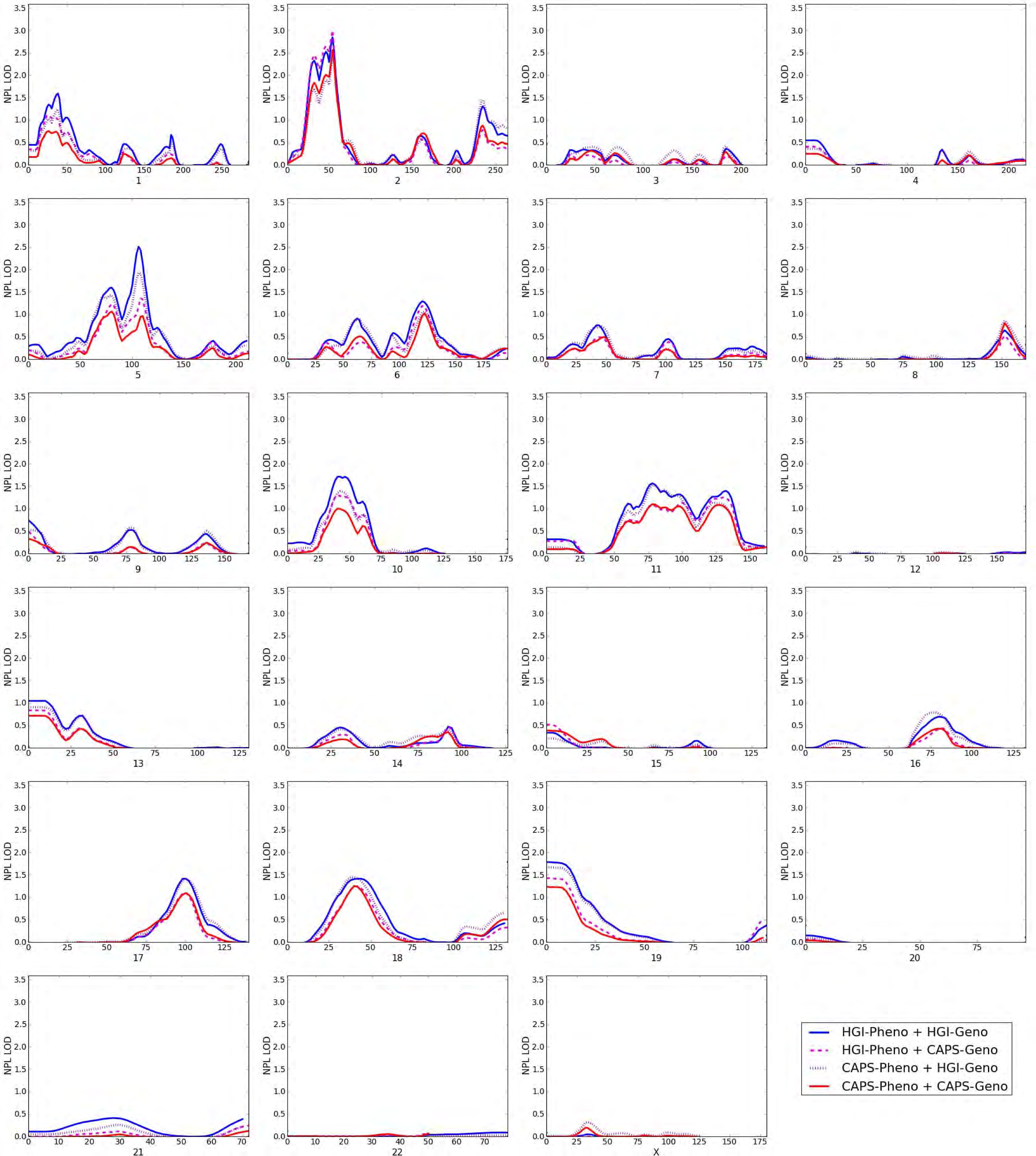

Study 6 Hispanic

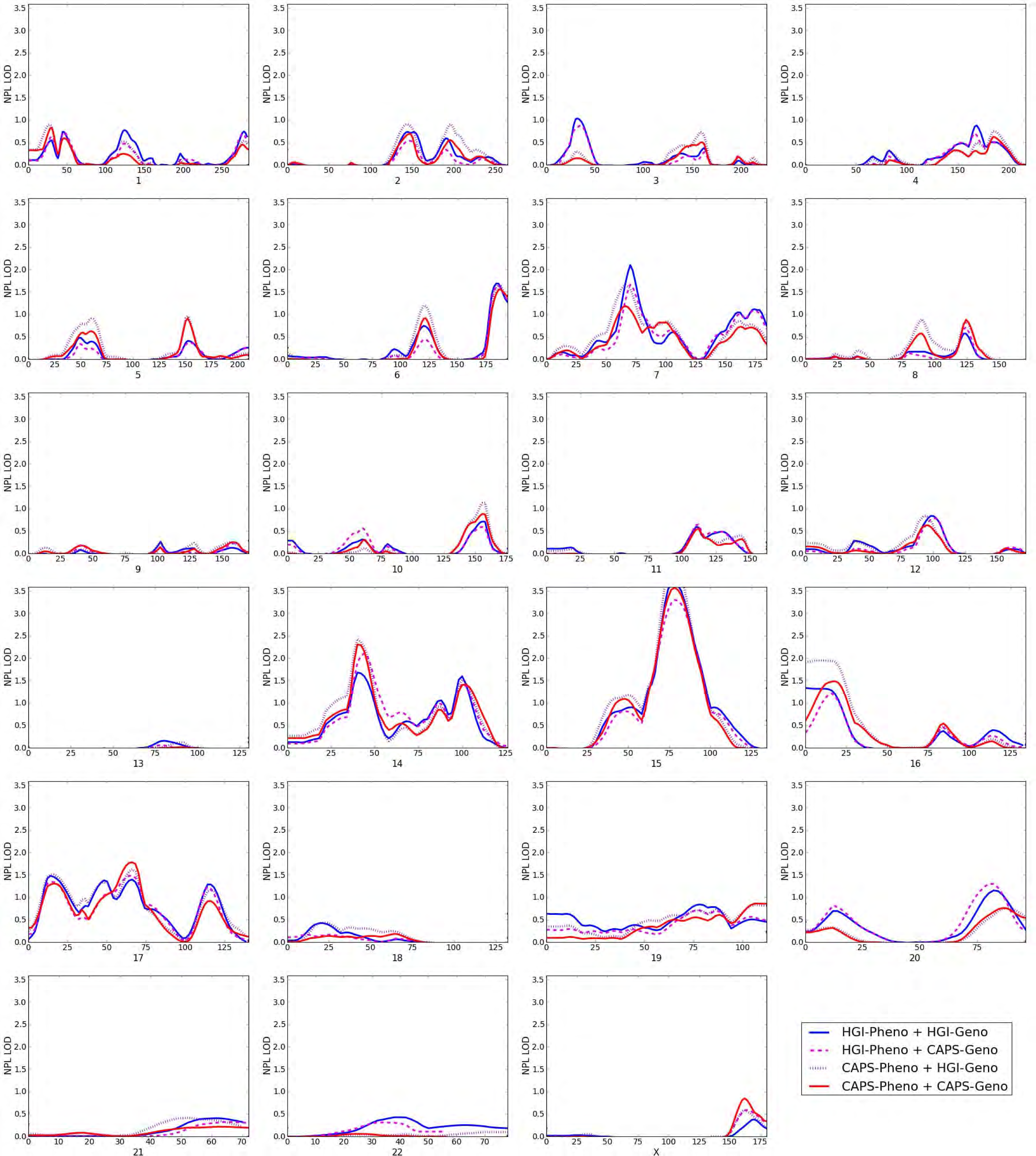

Study 7 African American

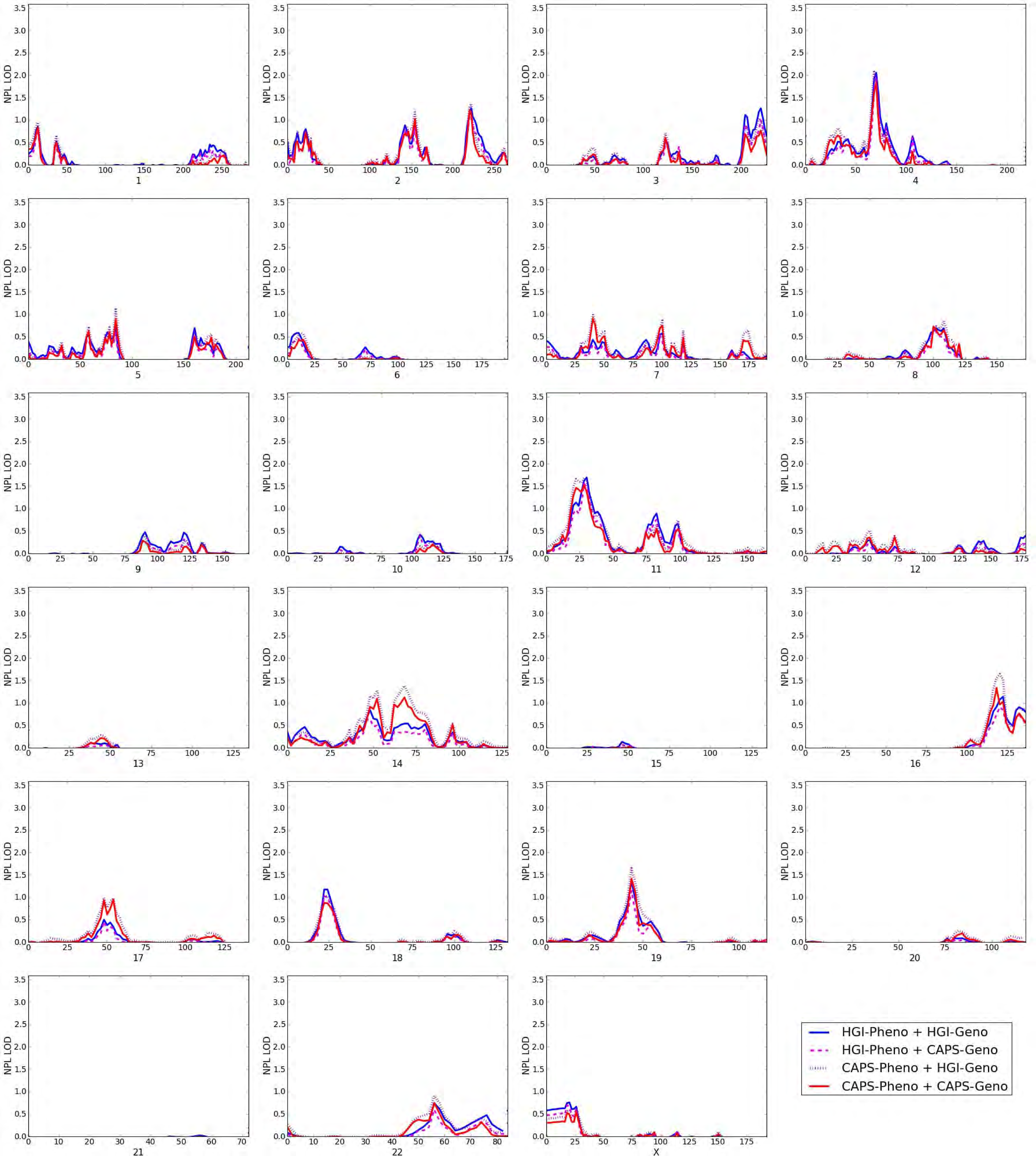

Supplement: Appendix S3 — Complete linkage results by subset over all processing states. The labels for each line indicate state of phenotype (Pheno) and genotype (Geno) data, which can be Human Genetics Initiative (HGI) or Combined Analysis of Psychiatric Studies (CAPS). (PDF) [file pone.0084696.s003.pdf]
