# Supplementary material for: Meta-Analysis of Repository Data: Impact of Data Regularization on NIMH Schizophrenia Linkage Results
Source: PLoS One. 2014 Jan 14;9(1):e84696. doi: 10.1371/journal.pone.0084696 (PMC3891773; doi:10.1371/journal.pone.0084696)

META-ANALYSIS VERSUS POOLED "MEGA-ANALYSIS."

In the main text we analyzed the multi-site HGI data using meta-analysis. An alternative approach is to simply combine all data into a single file for "pooled" or what is sometimes called "mega-analysis." We chose meta-analysis as the primary approach in part in order to take advantage of group-specific genetic maps, since genetic distances can vary across major population groups. By contrast, in mega-analysis it is necessary to impose a single genetic map across all groups.

However, for completeness, we include mega-analysis results here. To do this, we combined all genotypic data into a single file, forcing unique names for any markers recurring across studies, and assigning slight offsets (0.001 cM) between any resulting pairs of duplicate markers. Genetic map positions were based on the common reference map (see main text). Group-specific marker allele frequency estimates were used. All families were combined into a single file, with genotypes coded as 'missing' for all markers other than those which a family was genotyped.

The figure below summarizes results of comparing HGI-data with CAPS-data based on mega-analysis. Overall comparative results are comparable to what is shown in Figure 3 in the main text, with the exception of chromosome 10, where the signal goes up after CAPS processing based on meta-analysis but down based on mega-analysis. This signal is driven by the Han Chinese group and is therefore confounded by the change in genetic map. Other salient differences (especially on chromosomes 11, 15) reflect in part the impact of using population-specific maps in the main analysis, and in part, small-sample differences between meta-analysis and mega-analysis. Note that largest $-\log_{10}$(p-values) are quite a bit larger here than in Figure 3 (main text); the mega-analysis also has the advantage of yielding narrower peaks, since it does not depend on binning. However, confounding by the inter-study gaps in marker maps is possibly an issue in interpretation here.

Nevertheless, the overall conclusion is in keeping with the results of GSMA: data processing and regularization changes the overall picture of the SZ genome, both in terms of magnitudes of peaks (in this case, with multiple peaks > 3) and in terms of the rank ordering of peaks.

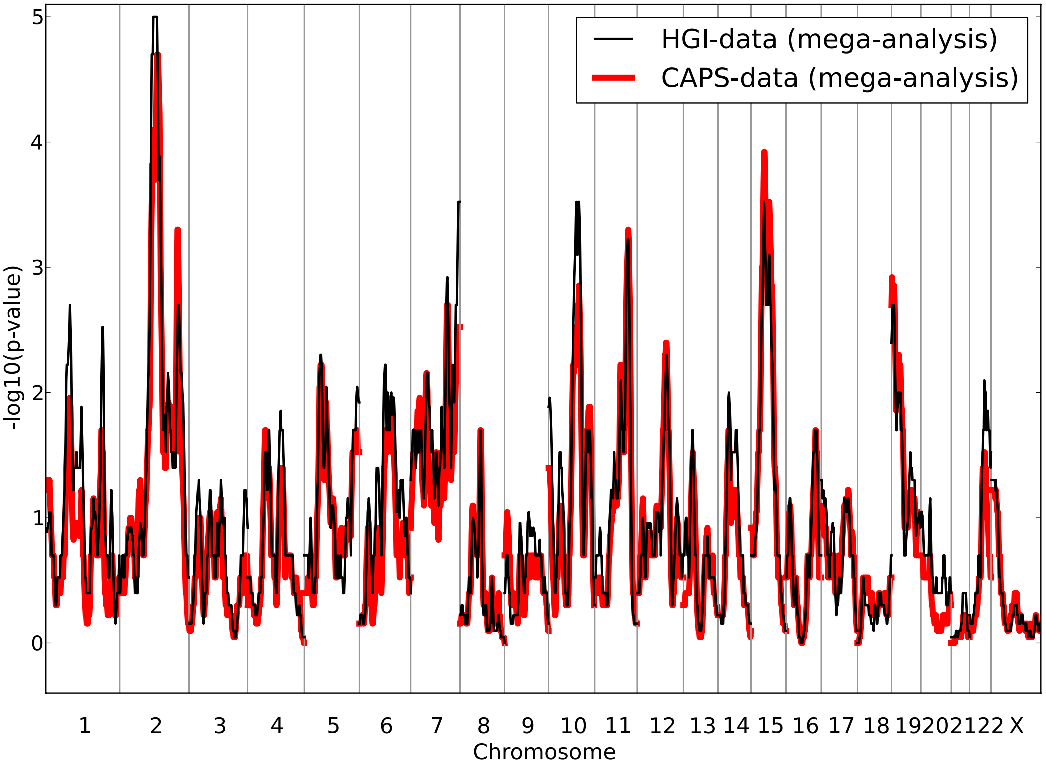

Supplement: Appendix S4 — Meta-analysis versus pooled mega-analysis. (PDF) [file pone.0084696.s004.pdf]
