# Supplementary material for: Meta-Analysis of Repository Data: Impact of Data Regularization on NIMH Schizophrenia Linkage Results
Source: PLoS One. 2014 Jan 14;9(1):e84696. doi: 10.1371/journal.pone.0084696 (PMC3891773; doi:10.1371/journal.pone.0084696)
Supplement: Table S1 — Complete list of family IDs and processing status indicators. Column header descriptions are as follows: dataset ID and family ID refer to NIMH Human Genetics Initiative (HGI) identifiers; merged indicates whether pedigree was joined during genotype processing by Combined Analysis of Psychiatric Studies (CAPS); CAPS-Geno indicates whether pedigree passed genotype regularization, and the excess Mendel or relationship error columns show reasons for failure; multiplex refers to families meeting both CAPS and HGI multiplex criteria; high bitsize indicates 16 pedigrees with bitsize of 25 or more; analysis indicates the 1,020 pedigrees used in this study, i.e., those passing CAPS-Geno, multiplex and not high bitsize. (PDF) [file pone.0084696.s005.pdf]

Dataset ID	Family ID	Merged	Mendel errors	Relationship errors	CAPS-Geno	Multiplex	High bitsize	Analysis
12	3501064	-	-	-	TRUE	TRUE	-	TRUE
12	3501073	-	-	-	TRUE	-	-	-
12	3501077	TRUE	-	-	TRUE	TRUE	-	TRUE
12	3501096	-	-	-	TRUE	TRUE	-	TRUE
12	3501119	-	-	-	TRUE	-	-	-
12	3501200	-	-	-	TRUE	TRUE	-	TRUE
12	3501266	-	-	-	TRUE	TRUE	-	TRUE
12	3501272	-	-	-	TRUE	TRUE	-	TRUE
12	3501607	-	-	-	TRUE	TRUE	-	TRUE
12	3501612	-	-	-	TRUE	TRUE	-	TRUE
12	3502240	-	-	-	TRUE	TRUE	-	TRUE
12	3502298	-	-	-	TRUE	TRUE	-	TRUE
12	3502347	-	-	-	TRUE	TRUE	-	TRUE
12	3502382	-	-	-	TRUE	TRUE	-	TRUE
12	3502383	-	-	-	TRUE	TRUE	-	TRUE
12	3502431	-	-	-	TRUE	TRUE	-	TRUE
12	3502435	-	-	-	TRUE	TRUE	-	TRUE
12	3502450	-	-	-	TRUE	TRUE	-	TRUE
12	3502451	-	-	-	TRUE	TRUE	-	TRUE
12	3502453	-	-	-	TRUE	TRUE	-	TRUE
12	3502478	-	-	-	TRUE	TRUE	-	TRUE
12	3502497	-	-	-	TRUE	TRUE	-	TRUE
12	3502578	-	-	-	TRUE	TRUE	-	TRUE
12	3503196	-	-	-	TRUE	TRUE	-	TRUE
12	3503603	-	-	-	TRUE	TRUE	-	TRUE
12	3504135	-	-	-	TRUE	TRUE	-	TRUE
12	3504138	-	-	-	TRUE	TRUE	-	TRUE
12	3504151	-	-	-	TRUE	TRUE	-	TRUE
12	3504163	-	-	-	TRUE	-	-	-
12	3504176	-	-	-	TRUE	TRUE	-	TRUE
12	3504187	-	-	-	TRUE	TRUE	-	TRUE
12	3504193	-	-	-	TRUE	TRUE	-	TRUE
12	3504194	-	-	-	TRUE	TRUE	-	TRUE
12	3504244	-	-	-	TRUE	-	-	-
12	3504267	-	-	-	TRUE	TRUE	-	TRUE
12	3504299	-	-	-	TRUE	TRUE	-	TRUE
12	3504317	-	-	-	TRUE	TRUE	-	TRUE
12	3504318	-	-	-	TRUE	TRUE	-	TRUE
12	3504329	-	-	-	TRUE	TRUE	-	TRUE
12	3504335	-	-	-	TRUE	-	-	-
12	3504338	-	-	-	TRUE	TRUE	-	TRUE
12	3504349	-	-	-	TRUE	TRUE	-	TRUE
12	3504353	-	-	-	TRUE	TRUE	-	TRUE
12	3504358	-	-	-	TRUE	TRUE	-	TRUE
12	3504361	-	-	-	TRUE	TRUE	-	TRUE
12	3504396	-	-	-	TRUE	TRUE	-	TRUE
12	3504398	-	-	-	TRUE	-	-	-
12	3504401	-	-	-	TRUE	TRUE	-	TRUE
12	3504404	-	-	-	TRUE	TRUE	-	TRUE
12	3504418	-	-	-	TRUE	-	-	-
12	3504419	-	-	-	TRUE	-	-	-
12	3504427	-	-	-	TRUE	TRUE	-	TRUE
12	3504442	-	-	-	TRUE	TRUE	-	TRUE
12	3504445	-	-	-	TRUE	TRUE	-	TRUE
12	3504455	-	-	-	TRUE	TRUE	-	TRUE
12	3504480	-	-	-	TRUE	TRUE	-	TRUE
12	3504515	-	-	-	TRUE	-	-	-

Dataset ID	Family ID	Merged	Mendel errors	Relationship errors	CAPS-Geno	Multiplex	High bitsize	Analysis
12	3504521	-	-	-	TRUE	TRUE	-	TRUE
12	3504524	TRUE	-	-	TRUE	TRUE	-	TRUE
12	3504529	-	-	-	TRUE	TRUE	-	TRUE
12	3504531	-	-	-	TRUE	TRUE	-	TRUE
12	3504537	-	-	-	TRUE	TRUE	-	TRUE
12	3504557	-	-	-	TRUE	TRUE	-	TRUE
12	3504560	-	-	-	TRUE	TRUE	-	TRUE
12	3504574	-	-	-	TRUE	TRUE	-	TRUE
12	3504597	-	-	-	TRUE	TRUE	-	TRUE
12	3504601	-	-	-	TRUE	TRUE	-	TRUE
12	3504610	-	-	-	TRUE	TRUE	-	TRUE
12	3505001	-	-	-	TRUE	TRUE	-	TRUE
12	3505221	-	-	-	TRUE	TRUE	-	TRUE
12	3505295	-	-	-	TRUE	TRUE	-	TRUE
12	3505359	-	-	-	TRUE	-	-	-
12	3505460	-	-	-	TRUE	TRUE	-	TRUE
12	3505498	-	-	-	TRUE	TRUE	-	TRUE
12	3505506	-	-	-	TRUE	TRUE	-	TRUE
12	3505543	-	-	-	TRUE	TRUE	-	TRUE
12	3505585	-	-	-	TRUE	TRUE	-	TRUE
12	3505587	TRUE	-	-	TRUE	TRUE	-	TRUE
12	3505598	-	-	-	TRUE	TRUE	-	TRUE
12	3505602	TRUE	-	-	TRUE	TRUE	-	TRUE
12	3505608	-	-	-	TRUE	TRUE	-	TRUE
12	3505615	-	-	-	TRUE	-	-	-
12	3506268	-	-	-	TRUE	TRUE	-	TRUE
12	3506277	-	-	-	TRUE	TRUE	-	TRUE
12	3506278	-	-	-	TRUE	TRUE	-	TRUE
12	3506331	-	-	-	TRUE	-	-	-
12	3506539	-	-	-	TRUE	TRUE	-	TRUE
12	3507111	-	-	-	TRUE	TRUE	-	TRUE
12	3507112	-	-	-	TRUE	-	-	-
12	3507178	-	-	-	TRUE	TRUE	-	TRUE
12	3507204	-	-	-	TRUE	TRUE	-	TRUE
12	3507257	-	-	-	TRUE	TRUE	-	TRUE
12	3507260	-	-	-	TRUE	TRUE	-	TRUE
12	3507372	-	-	-	TRUE	TRUE	-	TRUE
12	3507546	-	-	-	TRUE	TRUE	-	TRUE
12	3508160	-	-	-	TRUE	TRUE	-	TRUE
12	3508161	-	-	-	TRUE	TRUE	-	TRUE
12	3508162	-	-	-	TRUE	TRUE	-	TRUE
12	3508276	-	-	-	TRUE	TRUE	-	TRUE
12	3508286	-	-	-	TRUE	TRUE	-	TRUE
12	3508294	-	-	-	TRUE	TRUE	-	TRUE
12	3508400	-	-	-	TRUE	TRUE	-	TRUE
12	3508447	-	-	-	TRUE	TRUE	-	TRUE
12	3509217	-	-	-	TRUE	TRUE	-	TRUE
12	3509239	-	-	-	TRUE	TRUE	-	TRUE
12	3509370	-	-	-	TRUE	TRUE	-	TRUE
12	3510428	-	-	-	TRUE	TRUE	-	TRUE
12	3510489	-	-	-	TRUE	TRUE	-	TRUE
12	3510513	-	-	-	TRUE	TRUE	-	TRUE
12	3511222	-	-	-	TRUE	TRUE	-	TRUE
12	3511281	-	-	-	TRUE	TRUE	-	TRUE
12	3512219	-	-	-	TRUE	TRUE	-	TRUE
12	3512228	-	-	-	TRUE	TRUE	-	TRUE
12	3512229	-	-	-	TRUE	TRUE	-	TRUE

Dataset ID	Family ID	Merged	Mendel errors	Relationship errors	CAPS-Geno	Multiplex	High bitsize	Analysis
12	3512258	-	-	-	TRUE	-	-	-
12	3512259	-	-	-	TRUE	TRUE	-	TRUE
12	3512265	-	-	-	TRUE	TRUE	-	TRUE
12	3512313	-	-	-	TRUE	-	-	-
12	3513253	-	-	-	TRUE	TRUE	-	TRUE
12	3513523	-	-	-	TRUE	TRUE	-	TRUE
12	3513534	-	-	-	TRUE	TRUE	-	TRUE
12	3515471	-	-	-	TRUE	TRUE	-	TRUE
12	3516416	-	-	-	TRUE	TRUE	-	TRUE
12	3517540	-	-	-	TRUE	TRUE	-	TRUE
12	3518242	-	-	-	TRUE	TRUE	-	TRUE
12	3518300	-	-	-	TRUE	TRUE	-	TRUE
12	3520345	-	-	-	TRUE	TRUE	-	TRUE
12	3520397	-	-	-	TRUE	TRUE	-	TRUE
12	3521371	-	-	-	TRUE	TRUE	-	TRUE
12	3521415	-	-	-	TRUE	TRUE	-	TRUE
12	3521424	-	-	-	TRUE	-	-	-
12	3521430	-	-	-	TRUE	TRUE	-	TRUE
12	3521438	-	-	-	TRUE	TRUE	-	TRUE
12	3521446	-	-	-	TRUE	TRUE	-	TRUE
12	3521458	-	-	-	TRUE	TRUE	-	TRUE
12	3521463	-	-	-	TRUE	TRUE	-	TRUE
12	3521469	-	-	-	TRUE	-	-	-
12	3521472	-	-	-	TRUE	TRUE	-	TRUE
12	3521490	-	-	-	TRUE	TRUE	-	TRUE
12	3522549	-	-	-	TRUE	TRUE	-	TRUE
12	3522561	-	-	-	TRUE	TRUE	-	TRUE
12	3522576	-	-	-	TRUE	TRUE	-	TRUE
12	3524590	-	-	-	TRUE	TRUE	-	TRUE
12	3531518	-	-	-	TRUE	-	-	-
12	3531519	-	-	-	TRUE	-	-	-
12	3531535	-	-	-	TRUE	TRUE	-	TRUE
12	3531538	-	-	-	TRUE	TRUE	-	TRUE
12	3531573	-	-	-	TRUE	TRUE	-	TRUE
12	3531579	-	-	-	TRUE	-	-	-
12	3532220	-	-	-	TRUE	TRUE	-	TRUE
12	3532252	-	-	-	TRUE	-	-	-
12	3532422	-	-	-	TRUE	TRUE	-	TRUE
12	3532484	-	-	-	TRUE	-	-	-
12	3534316	-	-	-	TRUE	TRUE	-	TRUE
12	3534388	-	-	-	TRUE	TRUE	-	TRUE
12	3534411	-	-	-	TRUE	TRUE	-	TRUE
12	3534420	-	-	-	TRUE	TRUE	-	TRUE
12	3534503	-	-	-	TRUE	TRUE	-	TRUE
12	3534542	-	-	-	TRUE	TRUE	-	TRUE
12	3534586	-	-	-	TRUE	-	-	-
12	3534609	-	-	-	TRUE	TRUE	-	TRUE
12	3535623	-	-	-	TRUE	TRUE	-	TRUE
12	3537577	-	-	-	TRUE	TRUE	-	TRUE
12	3538575	-	-	-	TRUE	TRUE	-	TRUE
12	3539611	-	-	-	TRUE	TRUE	-	TRUE
12	3539614	-	-	-	TRUE	TRUE	-	TRUE
12	3539618	-	-	-	TRUE	TRUE	-	TRUE
12	3539622	-	-	-	TRUE	TRUE	-	TRUE
12	3540449	-	-	-	TRUE	TRUE	-	TRUE
12	3541001	-	-	-	TRUE	-	-	-
12	3541010	-	-	-	TRUE	TRUE	-	TRUE

Dataset ID	Family ID	Merged	Mendel errors	Relationship errors	CAPS-Geno	Multiplex	High bitsize	Analysis
12	3541011	-	-	-	TRUE	TRUE	-	TRUE
12	3541013	-	-	-	TRUE	TRUE	-	TRUE
12	3541025	-	-	-	TRUE	TRUE	-	TRUE
12	3541029	-	-	-	TRUE	TRUE	-	TRUE
12	3541030	-	-	-	TRUE	TRUE	-	TRUE
12	3541038	-	-	-	TRUE	-	-	-
12	3541065	-	-	-	TRUE	TRUE	-	TRUE
12	3541070	-	-	-	TRUE	TRUE	-	TRUE
12	3541071	-	-	-	TRUE	-	-	-
12	3541072	-	-	-	TRUE	TRUE	-	TRUE
12	3541080	-	-	-	TRUE	-	-	-
12	3541084	-	-	-	TRUE	TRUE	-	TRUE
12	3541090	-	-	-	TRUE	TRUE	-	TRUE
12	3541091	-	-	-	TRUE	TRUE	-	TRUE
12	3541101	-	-	-	TRUE	TRUE	-	TRUE
12	3541102	-	-	-	TRUE	TRUE	-	TRUE
12	3541166	-	-	-	TRUE	TRUE	-	TRUE
12	3541167	-	-	-	TRUE	TRUE	-	TRUE
12	3541177	-	-	-	TRUE	TRUE	-	TRUE
12	3541182	-	-	-	TRUE	TRUE	-	TRUE
12	3541183	-	-	-	TRUE	TRUE	-	TRUE
12	3541256	-	-	-	TRUE	TRUE	-	TRUE
12	3541273	-	-	-	TRUE	TRUE	-	TRUE
12	3541285	-	-	-	TRUE	TRUE	-	TRUE
12	3541288	-	-	-	TRUE	TRUE	-	TRUE
12	3541308	-	-	-	TRUE	TRUE	-	TRUE
12	3541408	-	-	-	TRUE	-	-	-
12	3541461	-	-	-	TRUE	TRUE	-	TRUE
12	3541462	-	-	-	TRUE	TRUE	-	TRUE
12	3541465	-	-	-	TRUE	TRUE	-	TRUE
12	3541467	-	-	-	TRUE	TRUE	-	TRUE
12	3541473	-	-	-	TRUE	-	-	-
12	3541477	-	-	-	TRUE	TRUE	-	TRUE
12	3541491	-	-	-	TRUE	TRUE	-	TRUE
12	3541494	-	-	-	TRUE	TRUE	-	TRUE
12	3541502	-	-	-	TRUE	-	-	-
12	3541508	-	-	-	TRUE	TRUE	-	TRUE
12	3541510	-	-	-	TRUE	TRUE	-	TRUE
12	3541522	-	-	-	TRUE	TRUE	-	TRUE
12	3541532	-	-	-	TRUE	TRUE	-	TRUE
12	3541550	-	-	-	TRUE	TRUE	-	TRUE
12	3541551	-	-	-	TRUE	TRUE	-	TRUE
12	3541555	-	-	-	TRUE	TRUE	-	TRUE
12	3541559	-	-	-	TRUE	TRUE	-	TRUE
12	3541564	-	-	-	TRUE	TRUE	-	TRUE
12	3541565	-	-	-	TRUE	-	-	-
12	3541588	-	-	-	TRUE	TRUE	-	TRUE
12	3541589	-	-	-	TRUE	TRUE	-	TRUE
12	3541596	-	-	-	TRUE	TRUE	-	TRUE
12	3541599	-	-	-	TRUE	TRUE	-	TRUE
12	3541600	-	-	-	TRUE	TRUE	-	TRUE
12	3541604	-	-	-	TRUE	TRUE	-	TRUE
12	3541606	-	-	-	TRUE	TRUE	-	TRUE
12	3542563	-	-	-	TRUE	TRUE	-	TRUE
12	3543197	-	-	-	TRUE	TRUE	-	TRUE
12	3543212	-	-	-	TRUE	TRUE	-	TRUE
12	3543287	-	-	-	TRUE	TRUE	-	TRUE

Dataset ID	Family ID	Merged	Mendel errors	Relationship errors	CAPS-Geno	Multiplex	High bitsize	Analysis
12	3543322	-	-	-	TRUE	TRUE	-	TRUE
12	3543378	-	-	-	TRUE	TRUE	-	TRUE
12	3543413	-	-	-	TRUE	-	-	-
12	3543493	-	-	-	TRUE	TRUE	-	TRUE
12	3543504	-	-	-	TRUE	TRUE	-	TRUE
12	3545021	-	-	-	TRUE	TRUE	-	TRUE
12	3545026	-	-	-	TRUE	TRUE	-	TRUE
12	3545085	-	-	-	TRUE	TRUE	-	TRUE
12	3545113	-	-	-	TRUE	TRUE	-	TRUE
12	3545140	-	-	-	TRUE	-	-	-
12	3545141	-	-	-	TRUE	TRUE	-	TRUE
12	3545198	-	-	-	TRUE	TRUE	-	TRUE
12	3545243	-	-	-	TRUE	TRUE	-	TRUE
12	3545459	-	-	-	TRUE	TRUE	-	TRUE
12	3545526	-	-	-	TRUE	TRUE	-	TRUE
12	3545556	-	-	-	TRUE	TRUE	-	TRUE
12	3546022	-	-	-	TRUE	TRUE	-	TRUE
12	3546082	-	-	-	TRUE	TRUE	-	TRUE
12	3546095	-	-	-	TRUE	-	-	-
12	3546099	-	-	-	TRUE	TRUE	-	TRUE
12	3546134	-	-	-	TRUE	TRUE	-	TRUE
12	3546269	-	-	-	TRUE	TRUE	-	TRUE
12	3546270	-	-	-	TRUE	TRUE	-	TRUE
12	3546290	-	-	-	TRUE	TRUE	-	TRUE
12	3546293	-	-	-	TRUE	TRUE	-	TRUE
12	3546330	-	-	-	TRUE	-	-	-
12	3546351	-	-	-	TRUE	TRUE	-	TRUE
12	3546363	-	-	-	TRUE	TRUE	-	TRUE
12	3546414	-	-	-	TRUE	TRUE	-	TRUE
12	3546562	-	-	-	TRUE	TRUE	-	TRUE
12	3547289	-	-	-	TRUE	TRUE	-	TRUE
12	3547393	-	-	-	TRUE	TRUE	-	TRUE
12	3547501	-	-	-	TRUE	TRUE	-	TRUE
12	3548311	-	-	-	TRUE	TRUE	-	TRUE
12	3548356	-	-	-	TRUE	TRUE	-	TRUE
12	3548357	-	-	-	TRUE	TRUE	-	TRUE
12	3548410	-	-	-	TRUE	-	-	-
12	3548511	-	-	-	TRUE	TRUE	-	TRUE
12	3549325	-	-	-	TRUE	TRUE	-	TRUE
12	3549426	-	-	-	TRUE	TRUE	-	TRUE
12	3549496	-	-	-	TRUE	TRUE	-	TRUE
12	3549595	-	-	-	TRUE	TRUE	-	TRUE
12	3550391	-	-	-	TRUE	TRUE	-	TRUE
12	3550464	-	-	-	TRUE	TRUE	-	TRUE
12	3550483	-	-	-	TRUE	TRUE	-	TRUE
12	3550505	-	-	-	TRUE	TRUE	-	TRUE
12	3550509	-	-	-	TRUE	TRUE	-	TRUE
12	3552475	-	-	-	TRUE	TRUE	-	TRUE
12	3552530	-	-	-	TRUE	TRUE	-	TRUE
12	3552545	-	-	-	TRUE	TRUE	-	TRUE
12	3552548	-	-	-	TRUE	TRUE	-	TRUE
12	3552554	-	-	-	TRUE	TRUE	-	TRUE
12	3552558	-	-	-	TRUE	TRUE	-	TRUE
12	3552567	-	-	-	TRUE	TRUE	-	TRUE
12	3552581	-	-	-	TRUE	TRUE	-	TRUE
12	3553487	-	-	-	TRUE	TRUE	-	TRUE
12	3553514	-	-	-	TRUE	TRUE	-	TRUE

Dataset ID	Family ID	Merged	Mendel errors	Relationship errors	CAPS-Geno	Multiplex	High bitsize	Analysis
12	3553516	-	-	-	TRUE	TRUE	-	TRUE
12	3553517	-	-	-	TRUE	TRUE	-	TRUE
12	3553525	-	-	-	TRUE	TRUE	-	TRUE
12	3554488	-	-	-	TRUE	TRUE	-	TRUE
12	3555499	-	-	-	TRUE	TRUE	-	TRUE
12	3555500	-	-	-	TRUE	TRUE	-	TRUE
12	3555566	-	-	-	TRUE	TRUE	-	TRUE
12	3555571	-	-	-	TRUE	TRUE	-	TRUE
12	3556012	-	-	-	TRUE	-	-	-
12	3556017	-	-	-	TRUE	TRUE	-	TRUE
12	3556041	-	-	-	TRUE	TRUE	-	TRUE
12	3556048	-	-	-	TRUE	TRUE	-	TRUE
12	3556067	-	-	-	TRUE	TRUE	-	TRUE
12	3556083	-	-	-	TRUE	TRUE	-	TRUE
12	3556092	-	-	-	TRUE	TRUE	-	TRUE
12	3556120	-	-	-	TRUE	TRUE	-	TRUE
12	3556173	-	-	-	TRUE	TRUE	-	TRUE
12	3556195	-	-	-	TRUE	TRUE	-	TRUE
12	3556214	-	-	-	TRUE	TRUE	-	TRUE
12	3556226	-	-	-	TRUE	TRUE	-	TRUE
12	3556241	-	-	-	TRUE	TRUE	-	TRUE
12	3556381	-	-	-	TRUE	TRUE	-	TRUE
12	3556423	-	-	-	TRUE	TRUE	-	TRUE
12	3556592	-	-	-	TRUE	TRUE	-	TRUE
12	3556593	-	-	-	TRUE	-	-	-
12	3557027	-	-	-	TRUE	TRUE	-	TRUE
12	3557028	-	-	-	TRUE	-	-	-
12	3557031	-	-	-	TRUE	TRUE	-	TRUE
12	3557033	-	-	-	TRUE	TRUE	-	TRUE
12	3557039	-	-	-	TRUE	TRUE	-	TRUE
12	3557043	-	-	-	TRUE	-	-	-
12	3557059	-	-	-	TRUE	TRUE	-	TRUE
12	3557060	-	-	-	TRUE	TRUE	-	TRUE
12	3557061	-	-	-	TRUE	TRUE	-	TRUE
12	3557063	-	-	-	TRUE	TRUE	-	TRUE
12	3557066	-	-	-	TRUE	TRUE	-	TRUE
12	3557068	-	-	-	TRUE	TRUE	-	TRUE
12	3557069	-	-	-	TRUE	-	-	-
12	3557079	-	-	-	TRUE	TRUE	-	TRUE
12	3557081	-	-	-	TRUE	-	-	-
12	3557087	-	-	-	TRUE	TRUE	-	TRUE
12	3557088	-	-	-	TRUE	-	-	-
12	3557089	-	-	-	TRUE	-	-	-
12	3557093	-	-	-	TRUE	-	-	-
12	3557114	-	-	-	TRUE	TRUE	-	TRUE
12	3557115	-	-	-	TRUE	TRUE	-	TRUE
12	3557122	-	-	-	TRUE	TRUE	-	TRUE
12	3557123	-	-	-	TRUE	TRUE	-	TRUE
12	3557124	-	-	-	TRUE	TRUE	-	TRUE
12	3557125	-	-	-	TRUE	-	-	-
12	3557126	-	-	-	TRUE	TRUE	-	TRUE
12	3557131	-	-	-	TRUE	TRUE	-	TRUE
12	3557145	-	-	-	TRUE	TRUE	-	TRUE
12	3557146	-	-	-	TRUE	TRUE	-	TRUE
12	3557154	-	-	-	TRUE	TRUE	-	TRUE
12	3557155	-	-	-	TRUE	TRUE	-	TRUE
12	3557169	-	-	-	TRUE	TRUE	-	TRUE

Dataset ID	Family ID	Merged	Mendel errors	Relationship errors	CAPS-Geno	Multiplex	High bitsize	Analysis
12	3557171	-	-	-	TRUE	TRUE	-	TRUE
12	3557172	-	-	-	TRUE	TRUE	-	TRUE
12	3557189	-	-	-	TRUE	TRUE	-	TRUE
12	3557191	-	-	-	TRUE	TRUE	-	TRUE
12	3557209	-	-	-	TRUE	TRUE	-	TRUE
12	3557215	-	-	-	TRUE	TRUE	-	TRUE
12	3557254	-	-	-	TRUE	TRUE	-	TRUE
12	3557369	-	-	-	TRUE	TRUE	-	TRUE
12	3557387	-	-	-	TRUE	TRUE	-	TRUE
12	3557407	-	-	-	TRUE	TRUE	-	TRUE
12	3557492	-	-	-	TRUE	TRUE	-	TRUE
12	3557533	-	-	-	TRUE	TRUE	-	TRUE
12	3557613	-	-	-	TRUE	TRUE	-	TRUE
12	3558109	-	-	-	TRUE	TRUE	-	TRUE
12	3558118	-	-	-	TRUE	TRUE	-	TRUE
12	3558150	-	-	-	TRUE	TRUE	-	TRUE
12	3559055	-	-	-	TRUE	TRUE	-	TRUE
12	3559076	-	-	-	TRUE	TRUE	-	TRUE
12	3559097	-	-	-	TRUE	TRUE	-	TRUE
12	3559107	TRUE	-	-	TRUE	TRUE	-	TRUE
12	3559108	-	-	-	TRUE	TRUE	-	TRUE
12	3559121	-	-	-	TRUE	TRUE	-	TRUE
12	3559132	-	-	-	TRUE	TRUE	-	TRUE
12	3559139	-	-	-	TRUE	TRUE	-	TRUE
12	3559144	TRUE	-	-	TRUE	TRUE	-	TRUE
12	3559157	-	-	-	TRUE	TRUE	-	TRUE
12	3560034	-	-	-	TRUE	-	-	-
12	3560035	-	-	-	TRUE	TRUE	-	TRUE
12	3560036	-	-	-	TRUE	TRUE	-	TRUE
12	3560044	-	-	-	TRUE	-	-	-
12	3560049	-	-	-	TRUE	TRUE	-	TRUE
12	3560086	-	-	-	TRUE	TRUE	-	TRUE
12	3561174	-	-	-	TRUE	TRUE	-	TRUE
12	3562186	-	-	-	TRUE	TRUE	-	TRUE
12	3562380	-	-	-	TRUE	TRUE	-	TRUE
12	3562482	-	-	-	TRUE	TRUE	-	TRUE
12	3562536	-	-	-	TRUE	-	-	-
12	3563233	-	-	-	TRUE	TRUE	-	TRUE
12	3563367	-	-	-	TRUE	TRUE	-	TRUE
12	3563368	-	-	-	TRUE	TRUE	-	TRUE
12	3563377	-	-	-	TRUE	TRUE	-	TRUE
12	3563389	-	-	-	TRUE	TRUE	-	TRUE
12	3563390	-	-	-	TRUE	-	-	-
12	3564292	-	-	-	TRUE	TRUE	-	TRUE
12	3565250	-	-	-	TRUE	TRUE	-	TRUE
12	3565251	-	-	-	TRUE	-	-	-
12	3565263	-	-	-	TRUE	TRUE	-	TRUE
12	3565323	-	-	-	TRUE	TRUE	-	TRUE
12	3565324	-	-	-	TRUE	TRUE	-	TRUE
12	3566352	-	-	-	TRUE	-	-	-
12	3566474	-	-	-	TRUE	TRUE	-	TRUE
12	3567366	-	-	-	TRUE	TRUE	-	TRUE
12	3568355	-	-	-	TRUE	TRUE	-	TRUE
12	3568443	-	-	-	TRUE	TRUE	-	TRUE
12	3568444	-	-	-	TRUE	TRUE	-	TRUE
12	3569452	-	-	-	TRUE	-	-	-
12	3570470	-	-	-	TRUE	TRUE	-	TRUE

Dataset ID	Family ID	Merged	Mendel errors	Relationship errors	CAPS-Geno	Multiplex	High bitsize	Analysis
12	3570486	-	-	-	TRUE	TRUE	-	TRUE
12	3570507	-	-	-	TRUE	TRUE	-	TRUE
12	3571005	-	-	-	TRUE	TRUE	-	TRUE
12	3571014	-	-	-	TRUE	-	-	-
12	3571015	-	-	-	TRUE	-	-	-
12	3571023	-	-	-	TRUE	TRUE	-	TRUE
12	3571024	-	-	-	TRUE	TRUE	-	TRUE
12	3571040	-	-	-	TRUE	TRUE	-	TRUE
12	3571042	-	-	-	TRUE	TRUE	-	TRUE
12	3571046	-	-	-	TRUE	TRUE	-	TRUE
12	3571062	-	-	-	TRUE	TRUE	-	TRUE
12	3571098	-	-	-	TRUE	TRUE	-	TRUE
12	3571100	-	-	-	TRUE	TRUE	-	TRUE
12	3571127	-	-	-	TRUE	TRUE	-	TRUE
12	3571128	-	-	-	TRUE	TRUE	-	TRUE
12	3571130	-	-	-	TRUE	TRUE	-	TRUE
12	3571133	-	-	-	TRUE	TRUE	-	TRUE
12	3571147	-	-	-	TRUE	TRUE	-	TRUE
12	3571148	-	-	-	TRUE	TRUE	-	TRUE
12	3571153	-	-	-	TRUE	TRUE	-	TRUE
12	3571158	-	-	-	TRUE	TRUE	-	TRUE
12	3571164	-	-	-	TRUE	TRUE	-	TRUE
12	3571188	-	-	-	TRUE	TRUE	-	TRUE
12	3571201	-	-	-	TRUE	TRUE	-	TRUE
12	3571248	-	-	-	TRUE	TRUE	-	TRUE
12	3571279	-	-	-	TRUE	-	-	-
12	3571283	-	-	-	TRUE	TRUE	-	TRUE
12	3571301	-	-	-	TRUE	TRUE	-	TRUE
12	3571336	-	-	-	TRUE	TRUE	-	TRUE
12	3571337	-	-	-	TRUE	TRUE	-	TRUE
12	3571342	-	-	-	TRUE	TRUE	-	TRUE
12	3571343	-	-	-	TRUE	TRUE	-	TRUE
12	3571346	-	-	-	TRUE	TRUE	-	TRUE
12	3571412	-	-	-	TRUE	-	-	-
12	3571433	-	-	-	TRUE	TRUE	-	TRUE
12	3571434	-	-	-	TRUE	TRUE	-	TRUE
12	3571436	-	-	-	TRUE	TRUE	-	TRUE
12	3571437	-	-	-	TRUE	TRUE	-	TRUE
12	3571485	-	-	-	TRUE	TRUE	-	TRUE
12	3571594	-	-	-	TRUE	TRUE	-	TRUE
12	3572006	-	-	-	TRUE	TRUE	-	TRUE
12	3572007	-	-	-	TRUE	TRUE	-	TRUE
12	3572009	-	-	-	TRUE	TRUE	-	TRUE
12	3572016	-	-	-	TRUE	TRUE	-	TRUE
12	3572051	-	-	-	TRUE	TRUE	-	TRUE
12	3572075	-	-	-	TRUE	TRUE	-	TRUE
12	3572143	-	-	-	TRUE	TRUE	-	TRUE
12	3573047	-	-	-	TRUE	TRUE	-	TRUE
12	3573054	-	-	-	TRUE	TRUE	-	TRUE
12	3573058	-	-	-	TRUE	TRUE	-	TRUE
12	3573105	-	-	-	TRUE	TRUE	-	TRUE
12	3573142	-	-	-	TRUE	TRUE	-	TRUE
12	3573175	-	-	-	TRUE	TRUE	-	TRUE
12	3573216	-	-	-	TRUE	TRUE	-	TRUE
12	3573223	-	-	-	TRUE	TRUE	-	TRUE
12	3573280	-	-	-	TRUE	-	-	-
12	3573291	-	-	-	TRUE	TRUE	-	TRUE

Dataset ID	Family ID	Merged	Mendel errors	Relationship errors	CAPS-Geno	Multiplex	High bitsize	Analysis
12	3573360	-	-	-	TRUE	-	-	-
12	3573432	-	-	-	TRUE	TRUE	-	TRUE
12	3573454	-	-	-	TRUE	TRUE	-	TRUE
12	3573457	-	-	-	TRUE	TRUE	-	TRUE
12	3573476	-	-	-	TRUE	-	-	-
12	3573520	-	-	-	TRUE	TRUE	-	TRUE
12	3573528	-	-	-	TRUE	TRUE	-	TRUE
12	3574106	-	-	-	TRUE	TRUE	-	TRUE
12	3574110	-	-	-	TRUE	TRUE	-	TRUE
12	3574137	-	-	-	TRUE	TRUE	-	TRUE
12	3574168	-	-	-	TRUE	TRUE	-	TRUE
12	3574255	-	-	-	TRUE	TRUE	-	TRUE
12	3574296	-	-	-	TRUE	-	-	-
12	3574409	-	-	-	TRUE	-	-	-
12	3574417	-	-	-	TRUE	TRUE	-	TRUE
12	3574439	-	-	-	TRUE	TRUE	-	TRUE
12	3575184	-	-	-	TRUE	TRUE	-	TRUE
12	3575185	-	-	-	TRUE	TRUE	-	TRUE
12	3575202	-	-	-	TRUE	-	-	-
12	3575208	-	-	-	TRUE	TRUE	-	TRUE
12	3575211	-	-	-	TRUE	TRUE	-	TRUE
12	3575225	-	-	-	TRUE	TRUE	-	TRUE
12	3575227	-	-	-	TRUE	TRUE	-	TRUE
12	3575232	-	-	-	TRUE	TRUE	-	TRUE
12	3575375	-	-	-	TRUE	TRUE	-	TRUE
12	3576008	-	-	-	TRUE	-	-	-
12	3576045	-	-	-	TRUE	TRUE	-	TRUE
12	3576053	-	-	-	TRUE	TRUE	-	TRUE
12	3576074	-	-	-	TRUE	TRUE	-	TRUE
12	3576078	-	-	-	TRUE	TRUE	-	TRUE
12	3576116	-	-	-	TRUE	TRUE	-	TRUE
12	3576206	-	-	-	TRUE	TRUE	-	TRUE
12	3576213	-	-	-	TRUE	-	-	-
12	3576297	-	-	-	TRUE	TRUE	-	TRUE
12	3576333	-	-	-	TRUE	TRUE	-	TRUE
12	3576376	-	-	-	TRUE	TRUE	-	TRUE
12	3576394	-	-	-	TRUE	TRUE	-	TRUE
12	3576395	-	-	-	TRUE	TRUE	-	TRUE
12	3577152	-	-	-	TRUE	TRUE	-	TRUE
12	3577165	-	-	-	TRUE	TRUE	-	TRUE
12	3577170	-	-	-	TRUE	TRUE	-	TRUE
12	3577180	-	-	-	TRUE	TRUE	-	TRUE
12	3577181	-	-	-	TRUE	TRUE	-	TRUE
12	3577190	-	-	-	TRUE	TRUE	-	TRUE
12	3578117	-	-	-	TRUE	-	-	-
12	3578136	-	-	-	TRUE	TRUE	-	TRUE
12	3578149	-	-	-	TRUE	TRUE	-	TRUE
12	3578247	-	-	-	TRUE	TRUE	-	TRUE
12	3578274	-	-	-	TRUE	-	-	-
12	3579199	-	-	-	TRUE	TRUE	-	TRUE
12	3579205	-	-	-	TRUE	TRUE	-	TRUE
12	3579275	-	-	-	TRUE	-	-	-
12	3579307	-	-	-	TRUE	TRUE	-	TRUE
12	3579309	-	-	-	TRUE	TRUE	-	TRUE
12	3579332	-	-	-	TRUE	-	-	-
12	3579350	-	-	-	TRUE	TRUE	-	TRUE
12	3579373	-	-	-	TRUE	-	-	-

Dataset ID	Family ID	Merged	Mendel errors	Relationship errors	CAPS-Geno	Multiplex	High bitsize	Analysis
12	3579399	-	-	-	TRUE	TRUE	-	TRUE
12	3579466	-	-	-	TRUE	TRUE	-	TRUE
12	3579495	-	-	-	TRUE	TRUE	-	TRUE
12	3579541	-	-	-	TRUE	TRUE	-	TRUE
12	3579553	-	-	-	TRUE	TRUE	-	TRUE
12	3580234	-	-	-	TRUE	-	-	-
12	3580235	-	-	-	TRUE	TRUE	-	TRUE
12	3581339	-	-	-	TRUE	TRUE	-	TRUE
12	3581341	-	-	-	TRUE	TRUE	-	TRUE
12	3581365	-	-	-	TRUE	TRUE	-	TRUE
12	3581374	-	-	-	TRUE	TRUE	-	TRUE
12	3581384	-	-	-	TRUE	TRUE	-	TRUE
12	3581440	-	-	-	TRUE	TRUE	-	TRUE
12	3582456	-	-	-	TRUE	TRUE	-	TRUE
12	3590584	-	-	-	TRUE	TRUE	-	TRUE
12	3592001	-	-	-	TRUE	-	-	-
12	3592002	-	-	-	TRUE	-	-	-
12	3592018	-	-	-	TRUE	TRUE	-	TRUE
12	3592037	-	-	-	TRUE	TRUE	-	TRUE
12	3592104	-	-	-	TRUE	-	-	-
12	3592264	-	-	-	TRUE	TRUE	-	TRUE
12	3592282	-	-	-	TRUE	TRUE	-	TRUE
12	3592302	-	-	-	TRUE	TRUE	-	TRUE
12	3592315	-	-	-	TRUE	TRUE	-	TRUE
12	3592319	-	-	-	TRUE	TRUE	-	TRUE
12	3592320	-	-	-	TRUE	TRUE	-	TRUE
12	3592354	-	-	-	TRUE	TRUE	-	TRUE
12	3592386	-	-	-	TRUE	TRUE	-	TRUE
12	3592406	-	-	-	TRUE	TRUE	-	TRUE
12	3592568	-	-	-	TRUE	TRUE	-	TRUE
12	3592569	-	-	-	TRUE	TRUE	-	TRUE
12	3592570	-	-	-	TRUE	TRUE	-	TRUE
12	3593050	-	-	-	TRUE	TRUE	-	TRUE
12	3593103	-	-	-	TRUE	TRUE	-	TRUE
12	3593156	-	-	-	TRUE	TRUE	-	TRUE
12	3593192	-	-	-	TRUE	TRUE	-	TRUE
12	3593306	-	-	-	TRUE	TRUE	-	TRUE
12	3593310	-	-	-	TRUE	TRUE	-	TRUE
12	3593321	-	-	-	TRUE	TRUE	-	TRUE
12	3593326	-	-	-	TRUE	TRUE	-	TRUE
12	3593327	-	-	-	TRUE	TRUE	-	TRUE
12	3593328	-	-	-	TRUE	-	-	-
12	3593340	-	-	-	TRUE	TRUE	-	TRUE
12	3593364	-	-	-	TRUE	TRUE	-	TRUE
12	3593402	-	-	-	TRUE	TRUE	-	TRUE
12	3593405	-	-	-	TRUE	-	-	-
12	3593441	-	-	-	TRUE	TRUE	-	TRUE
12	3593468	-	-	-	TRUE	TRUE	-	TRUE
12	3593479	-	-	-	TRUE	TRUE	-	TRUE
12	3594057	-	-	-	TRUE	TRUE	-	TRUE
12	3595348	-	-	-	TRUE	TRUE	-	TRUE
12	3595392	-	-	-	TRUE	TRUE	-	TRUE
12	3595429	-	-	-	TRUE	TRUE	-	TRUE
12	3595527	-	-	-	TRUE	-	-	-
12	3596236	-	-	-	TRUE	TRUE	-	TRUE
12	3596237	-	-	-	TRUE	TRUE	-	TRUE
12	3596245	-	-	-	TRUE	TRUE	-	TRUE

Dataset ID	Family ID	Merged	Mendel errors	Relationship errors	CAPS-Geno	Multiplex	High bitsize	Analysis
12	3596334	-	-	-	TRUE	TRUE	-	TRUE
12	3596379	-	-	-	TRUE	TRUE	-	TRUE
12	3599425	-	-	-	TRUE	TRUE	-	TRUE
12	3599552	-	-	-	TRUE	TRUE	-	TRUE
21	7111	-	-	-	TRUE	TRUE	-	TRUE
21	7133	-	-	-	TRUE	TRUE	-	TRUE
21	7144	-	-	-	TRUE	TRUE	TRUE	-
21	701032	-	-	-	TRUE	TRUE	TRUE	-
21	701071	-	-	-	TRUE	-	-	-
21	701073	-	-	-	TRUE	TRUE	-	TRUE
21	701086	-	-	-	TRUE	TRUE	-	TRUE
21	701088	-	-	-	TRUE	TRUE	TRUE	-
21	701096	-	-	-	TRUE	TRUE	TRUE	-
21	701103	-	-	-	TRUE	-	-	-
21	701104	-	-	-	TRUE	TRUE	-	TRUE
21	701105	-	-	-	TRUE	-	-	-
21	701106	-	-	-	TRUE	-	-	-
21	701112	-	-	-	TRUE	TRUE	-	TRUE
21	701120	-	-	-	TRUE	TRUE	TRUE	-
21	701123	-	-	-	TRUE	TRUE	-	TRUE
21	701130	-	-	-	TRUE	TRUE	TRUE	-
21	701135	-	-	-	TRUE	-	-	-
21	701136	-	-	-	TRUE	TRUE	TRUE	-
21	701140	-	-	-	TRUE	TRUE	TRUE	-
21	701153	-	-	-	TRUE	-	-	-
21	701170	-	-	-	TRUE	TRUE	-	TRUE
21	701179	-	-	-	TRUE	TRUE	-	TRUE
21	701182	-	-	-	TRUE	-	-	-
21	701184	-	-	-	TRUE	-	-	-
21	701214	-	-	-	TRUE	TRUE	-	TRUE
21	715077	-	-	-	TRUE	TRUE	TRUE	-
21	7110250	-	-	-	TRUE	TRUE	TRUE	-
21	7120136	-	-	-	TRUE	TRUE	TRUE	-
21	7126743	-	-	-	TRUE	TRUE	-	TRUE
21	7140080	-	-	-	TRUE	TRUE	TRUE	-
21	7152060	-	-	-	TRUE	TRUE	-	TRUE
21	7170010	-	-	-	TRUE	TRUE	-	TRUE
21	7175019	-	-	-	TRUE	TRUE	TRUE	-
21	7176112	-	-	-	TRUE	-	-	-
21	7180048	-	-	-	TRUE	TRUE	-	TRUE
21	7180306	-	-	-	TRUE	TRUE	-	TRUE
21	7185236	-	-	-	TRUE	TRUE	-	TRUE
21	7185501	-	-	-	TRUE	TRUE	-	TRUE
21	7196065	-	-	-	TRUE	-	-	-
22	53101	-	-	-	TRUE	TRUE	-	TRUE
22	53102	-	-	-	TRUE	TRUE	-	TRUE
22	53106	-	-	-	TRUE	TRUE	-	TRUE
22	53107	-	-	-	TRUE	TRUE	-	TRUE
22	53108	-	-	-	TRUE	TRUE	-	TRUE
22	53111	-	-	-	TRUE	-	-	-
22	53118	-	-	-	TRUE	-	-	-
22	54101	-	-	-	TRUE	TRUE	-	TRUE
22	54102	-	-	-	TRUE	-	-	-
22	54104	-	-	-	TRUE	TRUE	-	TRUE
22	54106	-	-	-	TRUE	-	-	-
22	54107	-	-	-	TRUE	TRUE	-	TRUE
22	54108	-	-	-	TRUE	TRUE	-	TRUE

Dataset ID	Family ID	Merged	Mendel errors	Relationship errors	CAPS-Geno	Multiplex	High bitsize	Analysis
22	54109	-	-	-	TRUE	TRUE	-	TRUE
22	54110	-	-	-	TRUE	TRUE	-	TRUE
22	54111	-	-	-	TRUE	-	-	-
22	54112	-	-	-	TRUE	-	-	-
22	54115	-	-	-	TRUE	TRUE	-	TRUE
22	54116	-	-	-	TRUE	-	-	-
22	54117	-	-	-	TRUE	-	-	-
22	54118	-	-	-	TRUE	TRUE	-	TRUE
22	54119	-	-	-	TRUE	-	-	-
22	54121	-	-	-	TRUE	TRUE	-	TRUE
22	54123	-	-	-	TRUE	-	-	-
22	54124	-	-	-	TRUE	TRUE	-	TRUE
22	54125	-	-	-	TRUE	-	-	-
22	55101	-	-	-	TRUE	-	-	-
22	55102	-	-	-	TRUE	TRUE	-	TRUE
22	55103	-	-	-	TRUE	TRUE	-	TRUE
22	55104	-	-	-	TRUE	TRUE	-	TRUE
22	55105	-	-	-	TRUE	TRUE	-	TRUE
22	55106	-	-	-	TRUE	-	-	-
22	56102	-	-	-	TRUE	TRUE	-	TRUE
22	56103	-	-	-	TRUE	TRUE	-	TRUE
22	56104	-	-	-	TRUE	TRUE	-	TRUE
22	56105	-	-	-	TRUE	TRUE	-	TRUE
22	56106	-	-	-	TRUE	-	-	-
22	56107	-	-	-	TRUE	TRUE	-	TRUE
22	56108	-	-	-	TRUE	TRUE	-	TRUE
22	56109	-	-	-	TRUE	TRUE	-	TRUE
22	56110	-	-	-	TRUE	TRUE	-	TRUE
22	56111	-	-	-	TRUE	TRUE	-	TRUE
22	56112	-	-	-	TRUE	TRUE	-	TRUE
22	56113	-	-	-	TRUE	TRUE	-	TRUE
22	56114	-	-	-	TRUE	TRUE	-	TRUE
22	56115	-	-	-	TRUE	TRUE	-	TRUE
22	56116	-	-	-	TRUE	TRUE	-	TRUE
22	56117	-	-	-	TRUE	TRUE	-	TRUE
22	56120	-	-	-	TRUE	TRUE	-	TRUE
22	56121	-	-	-	TRUE	TRUE	-	TRUE
22	56122	-	-	-	TRUE	TRUE	-	TRUE
22	56123	-	-	-	TRUE	TRUE	-	TRUE
22	56126	-	-	-	TRUE	TRUE	-	TRUE
22	56127	-	-	-	TRUE	TRUE	-	TRUE
22	56128	-	-	-	TRUE	TRUE	-	TRUE
22	56129	-	-	-	TRUE	TRUE	-	TRUE
22	56130	-	-	-	TRUE	TRUE	-	TRUE
22	56131	-	-	-	TRUE	-	-	-
22	56132	-	-	-	TRUE	TRUE	-	TRUE
22	57101	-	-	-	TRUE	-	-	-
22	57103	-	-	-	TRUE	TRUE	-	TRUE
22	57104	-	-	-	TRUE	TRUE	-	TRUE
22	57105	-	-	-	TRUE	TRUE	-	TRUE
22	57107	-	-	-	TRUE	TRUE	-	TRUE
22	57108	-	-	-	TRUE	TRUE	-	TRUE
22	57109	-	-	-	TRUE	TRUE	-	TRUE
22	57110	-	-	-	TRUE	TRUE	-	TRUE
22	57111	-	-	TRUE	-	-	-	-
22	57112	-	-	-	TRUE	TRUE	-	TRUE
22	57113	-	-	-	TRUE	TRUE	-	TRUE

Dataset ID	Family ID	Merged	Mendel errors	Relationship errors	CAPS-Geno	Multiplex	High bitsize	Analysis
22	57114	-	-	-	TRUE	TRUE	-	TRUE
22	57115	-	-	-	TRUE	TRUE	-	TRUE
22	57116	-	-	-	TRUE	TRUE	-	TRUE
22	57118	-	-	-	TRUE	-	-	-
22	57119	-	-	-	TRUE	TRUE	-	TRUE
22	57121	-	-	-	TRUE	-	-	-
22	57122	-	-	-	TRUE	TRUE	-	TRUE
22	57124	-	-	-	TRUE	TRUE	-	TRUE
22	57125	-	-	-	TRUE	-	-	-
22	57126	-	-	-	TRUE	TRUE	-	TRUE
22	57127	-	-	-	TRUE	-	-	-
22	57128	-	-	-	TRUE	TRUE	-	TRUE
22	58101	-	-	-	TRUE	-	-	-
22	58104	-	-	-	TRUE	TRUE	-	TRUE
22	58105	-	-	-	TRUE	TRUE	-	TRUE
22	58106	-	-	-	TRUE	-	-	-
22	58108	-	-	-	TRUE	TRUE	-	TRUE
22	58109	-	-	-	TRUE	TRUE	-	TRUE
22	58110	-	-	-	TRUE	TRUE	-	TRUE
22	58111	-	-	-	TRUE	-	-	-
22	58112	-	-	-	TRUE	TRUE	-	TRUE
22	58114	-	-	-	TRUE	-	-	-
22	58115	-	-	-	TRUE	TRUE	-	TRUE
22	58116	-	-	-	TRUE	-	-	-
22	58117	-	-	-	TRUE	TRUE	-	TRUE
23	53104	-	-	-	TRUE	TRUE	-	TRUE
23	53105	-	-	-	TRUE	TRUE	-	TRUE
23	53109	-	-	-	TRUE	TRUE	-	TRUE
23	53110	-	-	-	TRUE	TRUE	-	TRUE
23	53113	-	-	-	TRUE	TRUE	-	TRUE
23	53114	-	-	-	TRUE	TRUE	TRUE	-
23	53116	-	-	-	TRUE	TRUE	-	TRUE
23	53117	-	-	-	TRUE	TRUE	-	TRUE
23	53119	-	-	-	TRUE	TRUE	-	TRUE
23	53120	-	-	-	TRUE	-	-	-
23	53121	-	-	-	TRUE	TRUE	TRUE	-
23	53123	-	-	-	TRUE	-	-	-
23	53124	-	TRUE	-	-	-	-	-
23	53126	-	-	-	TRUE	-	-	-
23	53127	-	-	-	TRUE	-	-	-
23	53128	-	-	-	TRUE	TRUE	-	TRUE
23	53129	-	-	-	TRUE	TRUE	-	TRUE
23	53130	-	TRUE	-	-	-	-	-
23	53131	-	-	-	TRUE	TRUE	-	TRUE
23	53133	-	-	-	TRUE	-	-	-
23	53136	-	-	-	TRUE	-	-	-
23	53139	-	-	-	TRUE	TRUE	-	TRUE
23	53140	-	-	-	TRUE	-	-	-
23	53143	-	-	-	TRUE	-	-	-
23	53145	-	-	-	TRUE	-	-	-
23	53146	-	-	-	TRUE	-	-	-
23	53149	-	-	-	TRUE	-	-	-
23	53150	-	-	-	TRUE	-	-	-
23	53151	-	-	-	TRUE	-	-	-
23	53152	-	-	-	TRUE	-	-	-
23	53153	-	TRUE	-	-	-	-	-
23	53154	-	-	-	TRUE	-	-	-

Dataset ID	Family ID	Merged	Mendel errors	Relationship errors	CAPS-Geno	Multiplex	High bitsize	Analysis
23	53155	-	-	-	TRUE	TRUE	-	TRUE
23	53156	-	-	-	TRUE	-	-	-
23	53157	-	-	-	TRUE	-	-	-
23	53158	-	-	-	TRUE	TRUE	-	TRUE
23	54105	-	-	-	TRUE	TRUE	-	TRUE
23	54113	-	-	-	TRUE	TRUE	-	TRUE
23	54127	-	-	-	TRUE	TRUE	-	TRUE
23	54128	-	-	-	TRUE	-	-	-
23	54129	-	-	-	TRUE	TRUE	-	TRUE
23	54130	-	-	-	TRUE	-	-	-
23	54131	-	-	-	TRUE	-	-	-
23	54132	-	-	-	TRUE	-	-	-
23	54134	-	-	-	TRUE	-	-	-
23	54135	-	-	-	TRUE	-	-	-
23	54136	-	TRUE	-	-	-	-	-
23	54137	-	-	-	TRUE	-	-	-
23	54138	-	-	-	TRUE	TRUE	-	TRUE
23	54139	-	-	-	TRUE	-	-	-
23	54140	-	-	-	TRUE	-	-	-
23	54141	-	-	-	TRUE	TRUE	-	TRUE
23	54142	-	-	-	TRUE	-	-	-
23	54501	-	-	-	TRUE	TRUE	-	TRUE
23	54502	-	-	-	TRUE	TRUE	-	TRUE
23	54503	-	-	-	TRUE	-	-	-
23	54505	-	-	-	TRUE	-	-	-
23	54509	-	-	-	TRUE	TRUE	-	TRUE
23	54510	-	-	-	TRUE	-	-	-
23	54511	-	-	-	TRUE	-	-	-
23	54512	-	-	-	TRUE	TRUE	-	TRUE
23	54513	-	-	-	TRUE	-	-	-
23	54514	-	-	-	TRUE	-	-	-
23	54515	-	TRUE	-	-	-	-	-
23	54516	-	-	-	TRUE	TRUE	-	TRUE
23	54517	-	-	-	TRUE	-	-	-
23	55107	-	-	-	TRUE	-	-	-
23	55108	-	-	-	TRUE	TRUE	-	TRUE
23	55109	-	-	-	TRUE	-	-	-
23	55110	-	-	-	TRUE	TRUE	-	TRUE
23	56101	-	-	-	TRUE	TRUE	-	TRUE
23	56119	-	-	-	TRUE	TRUE	-	TRUE
23	56133	-	-	-	TRUE	TRUE	-	TRUE
23	56134	-	-	-	TRUE	TRUE	-	TRUE
23	56135	-	-	-	TRUE	TRUE	-	TRUE
23	56136	-	-	-	TRUE	TRUE	-	TRUE
23	56137	-	-	-	TRUE	-	-	-
23	56138	-	-	-	TRUE	TRUE	-	TRUE
23	56139	-	-	-	TRUE	TRUE	-	TRUE
23	56140	-	-	-	TRUE	TRUE	-	TRUE
23	56141	-	-	-	TRUE	TRUE	-	TRUE
23	56142	-	-	-	TRUE	TRUE	-	TRUE
23	56143	-	-	-	TRUE	TRUE	-	TRUE
23	56144	-	-	-	TRUE	TRUE	-	TRUE
23	56145	-	-	-	TRUE	TRUE	-	TRUE
23	56146	-	-	-	TRUE	TRUE	-	TRUE
23	56147	-	-	-	TRUE	-	-	-
23	56148	-	-	-	TRUE	TRUE	-	TRUE
23	56149	-	-	-	TRUE	-	-	-

Dataset ID	Family ID	Merged	Mendel errors	Relationship errors	CAPS-Geno	Multiplex	High bitsize	Analysis
23	56150	-	-	-	TRUE	TRUE	-	TRUE
23	56151	-	-	-	TRUE	TRUE	-	TRUE
23	56152	-	-	-	TRUE	TRUE	-	TRUE
23	56153	-	-	-	TRUE	-	-	-
23	56154	-	-	-	TRUE	TRUE	-	TRUE
23	56155	-	-	-	TRUE	TRUE	-	TRUE
23	56156	-	-	-	TRUE	-	-	-
23	56157	-	-	-	TRUE	-	-	-
23	56158	-	-	-	TRUE	TRUE	-	TRUE
23	56159	-	-	-	TRUE	-	-	-
23	56161	-	-	-	TRUE	TRUE	-	TRUE
23	56162	-	-	-	TRUE	-	-	-
23	56163	-	TRUE	-	-	-	-	-
23	56164	-	-	-	TRUE	-	-	-
23	56165	-	TRUE	-	-	-	-	-
23	56166	-	-	-	TRUE	-	-	-
23	56167	-	-	-	TRUE	-	-	-
23	56168	-	-	-	TRUE	-	-	-
23	56169	-	-	-	TRUE	TRUE	-	TRUE
23	56170	-	-	-	TRUE	-	-	-
23	56171	-	-	-	TRUE	-	-	-
23	56174	-	-	-	TRUE	TRUE	-	TRUE
23	56175	-	-	-	TRUE	TRUE	-	TRUE
23	56176	-	-	-	TRUE	TRUE	-	TRUE
23	56177	-	-	-	TRUE	TRUE	-	TRUE
23	56179	-	-	TRUE	-	-	-	-
23	56180	-	-	-	TRUE	-	-	-
23	56181	-	-	-	TRUE	TRUE	-	TRUE
23	56182	-	-	-	TRUE	-	-	-
23	56184	-	-	-	TRUE	TRUE	-	TRUE
23	56185	-	-	-	TRUE	-	-	-
23	56186	-	-	-	TRUE	TRUE	-	TRUE
23	56187	-	-	-	TRUE	TRUE	-	TRUE
23	56189	-	-	-	TRUE	TRUE	-	TRUE
23	56190	-	-	-	TRUE	TRUE	-	TRUE
23	56191	-	-	-	TRUE	-	-	-
23	56192	-	-	-	TRUE	TRUE	-	TRUE
23	56193	-	-	-	TRUE	-	-	-
23	56194	-	-	-	TRUE	-	-	-
23	56195	-	-	-	TRUE	TRUE	-	TRUE
23	56196	-	-	-	TRUE	TRUE	-	TRUE
23	56197	-	-	-	TRUE	-	-	-
23	56198	-	-	-	TRUE	TRUE	-	TRUE
23	56199	-	-	-	TRUE	TRUE	-	TRUE
23	56200	-	-	-	TRUE	TRUE	-	TRUE
23	57102	-	-	-	TRUE	TRUE	-	TRUE
23	57106	-	-	-	TRUE	TRUE	-	TRUE
23	57120	-	-	-	TRUE	-	-	-
23	57129	-	-	-	TRUE	-	-	-
23	57130	-	-	-	TRUE	TRUE	-	TRUE
23	57131	-	-	-	TRUE	-	-	-
23	57132	-	-	-	TRUE	TRUE	-	TRUE
23	57133	-	-	-	TRUE	TRUE	-	TRUE
23	57134	-	-	-	TRUE	TRUE	-	TRUE
23	57135	-	-	-	TRUE	TRUE	-	TRUE
23	57136	-	-	-	TRUE	TRUE	-	TRUE
23	57137	-	-	-	TRUE	-	-	-

Dataset ID	Family ID	Merged	Mendel errors	Relationship errors	CAPS-Geno	Multiplex	High bitsize	Analysis
23	57138	-	-	-	TRUE	TRUE	-	TRUE
23	57139	-	-	-	TRUE	TRUE	-	TRUE
23	57140	-	-	-	TRUE	-	-	-
23	57141	-	-	-	TRUE	-	-	-
23	57142	-	-	-	TRUE	-	-	-
23	57143	-	-	-	TRUE	TRUE	-	TRUE
23	57144	-	-	-	TRUE	TRUE	-	TRUE
23	57145	-	-	-	TRUE	TRUE	-	TRUE
23	57146	-	-	-	TRUE	TRUE	-	TRUE
23	57147	-	-	-	TRUE	TRUE	-	TRUE
23	57148	-	-	-	TRUE	TRUE	-	TRUE
23	57149	-	-	-	TRUE	TRUE	-	TRUE
23	57150	-	-	-	TRUE	TRUE	-	TRUE
23	57151	-	-	-	TRUE	TRUE	-	TRUE
23	57152	-	-	-	TRUE	TRUE	-	TRUE
23	57153	-	-	-	TRUE	TRUE	-	TRUE
23	57154	-	TRUE	-	-	-	-	-
23	57155	-	-	-	TRUE	TRUE	-	TRUE
23	57156	-	-	-	TRUE	TRUE	-	TRUE
23	57157	-	-	-	TRUE	-	-	-
23	57158	-	-	-	TRUE	TRUE	-	TRUE
23	57159	-	TRUE	-	-	-	-	-
23	57160	-	-	-	TRUE	TRUE	-	TRUE
23	58119	-	-	-	TRUE	-	-	-
23	58120	-	-	-	TRUE	-	-	-
23	59003	-	-	-	TRUE	-	-	-
23	59005	-	-	-	TRUE	TRUE	-	TRUE
23	59006	-	-	-	TRUE	TRUE	-	TRUE
23	59007	-	-	-	TRUE	-	-	-
23	59008	-	-	-	TRUE	TRUE	-	TRUE
23	59009	-	-	-	TRUE	-	-	-
23	59010	-	-	-	TRUE	TRUE	-	TRUE
23	59011	-	-	-	TRUE	-	-	-
23	59012	-	-	-	TRUE	TRUE	-	TRUE
23	59014	-	-	-	TRUE	TRUE	-	TRUE
23	59015	-	-	-	TRUE	TRUE	-	TRUE
23	59019	-	-	-	TRUE	TRUE	-	TRUE
23	59020	-	-	-	TRUE	-	-	-
23	59023	-	-	-	TRUE	TRUE	-	TRUE
24	10100131	-	-	-	TRUE	-	-	-
24	10100132	-	-	-	TRUE	TRUE	-	TRUE
24	10100134	-	-	-	TRUE	-	-	-
24	10100146	-	-	-	TRUE	-	-	-
24	10100252	-	-	-	TRUE	-	-	-
24	10100255	-	-	-	TRUE	-	-	-
24	10100257	-	-	-	TRUE	-	-	-
24	10100268	-	-	-	TRUE	-	-	-
24	10100269	-	-	-	TRUE	-	-	-
24	10100273	-	-	-	TRUE	-	-	-
24	10100376	-	-	-	TRUE	TRUE	-	TRUE
24	10100501	-	-	-	TRUE	-	-	-
24	10100503	-	-	-	TRUE	TRUE	-	TRUE
24	10100604	-	-	-	TRUE	-	-	-
24	10100605	-	-	-	TRUE	-	-	-
24	10100606	-	-	-	TRUE	TRUE	-	TRUE
24	10100609	-	-	-	TRUE	TRUE	-	TRUE
24	10100611	-	-	-	TRUE	-	-	-

Dataset ID	Family ID	Merged	Mendel errors	Relationship errors	CAPS-Geno	Multiplex	High bitsize	Analysis
24	10100613	-	-	-	TRUE	TRUE	-	TRUE
24	10100618	-	-	-	TRUE	TRUE	-	TRUE
24	10100619	-	-	-	TRUE	-	-	-
24	10210203	-	-	-	TRUE	-	-	-
24	10210208	-	-	-	TRUE	TRUE	TRUE	-
24	10210210	-	-	-	TRUE	-	-	-
24	10210223	-	-	-	TRUE	-	-	-
24	10210241	-	-	-	TRUE	TRUE	-	TRUE
24	10210242	-	-	-	TRUE	TRUE	-	TRUE
24	10220033	-	-	-	TRUE	-	-	-
24	10220176	-	-	-	TRUE	-	-	-
24	10220189	-	-	-	TRUE	TRUE	-	TRUE
24	10226722	-	-	-	TRUE	-	-	-
24	10226768	-	-	-	TRUE	-	-	-
24	10240214	-	-	-	TRUE	-	-	-
24	10246011	-	-	TRUE	-	-	-	-
24	10252021	-	-	-	TRUE	-	-	-
24	10252024	-	-	-	TRUE	-	-	-
24	10252067	-	-	-	TRUE	-	-	-
24	10255133	-	-	-	TRUE	-	-	-
24	10276138	-	-	-	TRUE	-	-	-
24	10276230	-	-	-	TRUE	-	-	-
24	10277043	-	-	-	TRUE	-	-	-
24	10278004	-	TRUE	-	-	-	-	-
24	10278013	-	-	-	TRUE	-	-	-
24	10296061	-	-	-	TRUE	-	-	-
24	10296076	-	-	-	TRUE	-	-	-
24	10400001	-	-	-	TRUE	TRUE	-	TRUE
24	10400006	-	-	-	TRUE	TRUE	-	TRUE
24	10400007	-	-	-	TRUE	TRUE	-	TRUE
24	10400009	-	-	-	TRUE	-	-	-
24	10400010	-	-	-	TRUE	-	-	-
24	10400011	-	-	-	TRUE	-	-	-
24	10400014	-	-	-	TRUE	-	-	-
24	10400016	-	-	-	TRUE	-	-	-
24	10400017	-	-	-	TRUE	-	-	-
24	10400020	-	-	-	TRUE	-	-	-
24	10400023	-	-	-	TRUE	-	-	-
24	10400030	-	-	-	TRUE	-	-	-
24	10400035	-	-	-	TRUE	-	-	-
24	10400042	-	-	-	TRUE	-	-	-
24	10400045	-	-	-	TRUE	-	-	-
24	10400053	-	-	-	TRUE	-	-	-
24	10400063	-	-	-	TRUE	-	-	-
24	10400066	-	-	-	TRUE	-	-	-
24	10400068	-	-	-	TRUE	-	-	-
24	10400069	-	-	-	TRUE	-	-	-
24	10400073	-	-	-	TRUE	-	-	-
24	10400076	-	-	-	TRUE	-	-	-
24	10400080	-	-	-	TRUE	-	-	-
24	10400082	-	-	-	TRUE	-	-	-
24	10400101	-	-	-	TRUE	-	-	-
24	10400104	-	-	-	TRUE	TRUE	-	TRUE
24	10530002	-	-	-	TRUE	TRUE	-	TRUE
24	10530003	-	-	-	TRUE	TRUE	-	TRUE
24	10530004	-	-	-	TRUE	-	-	-
24	10530006	-	-	-	TRUE	TRUE	-	TRUE

Dataset ID	Family ID	Merged	Mendel errors	Relationship errors	CAPS-Geno	Multiplex	High bitsize	Analysis
24	10530007	-	-	-	TRUE	-	-	-
24	10530008	-	-	-	TRUE	TRUE	-	TRUE
24	10530011	-	-	-	TRUE	TRUE	-	TRUE
24	10530012	-	-	-	TRUE	TRUE	-	TRUE
24	10530017	-	-	-	TRUE	-	-	-
24	10530018	-	-	-	TRUE	-	-	-
24	10530020	-	-	-	TRUE	-	-	-
24	10530026	-	-	-	TRUE	TRUE	-	TRUE
24	10530029	-	-	-	TRUE	-	-	-
24	10530039	-	-	-	TRUE	TRUE	-	TRUE
24	10530041	-	-	-	TRUE	TRUE	-	TRUE
24	10530044	-	-	-	TRUE	-	-	-
24	10530052	-	-	-	TRUE	-	-	-
24	10530053	-	-	-	TRUE	TRUE	-	TRUE
24	10530054	-	-	-	TRUE	-	-	-
24	10530062	-	-	-	TRUE	-	-	-
24	10530063	-	-	-	TRUE	-	-	-
24	10530064	-	-	-	TRUE	-	-	-
24	10530067	-	-	-	TRUE	-	-	-
24	10530068	-	-	-	TRUE	TRUE	-	TRUE
24	10530071	-	-	-	TRUE	-	-	-
24	10530072	-	-	-	TRUE	-	-	-
24	10530073	-	-	-	TRUE	-	-	-
24	10530078	-	-	-	TRUE	-	-	-
24	10530079	-	-	-	TRUE	-	-	-
24	10600251	-	-	-	TRUE	-	-	-
24	10600252	-	-	-	TRUE	-	-	-
24	10600259	-	-	-	TRUE	-	-	-
24	10600266	-	-	-	TRUE	-	-	-
24	10600270	-	-	-	TRUE	-	-	-
24	10600274	-	-	-	TRUE	-	-	-
24	10600281	-	-	-	TRUE	-	-	-
24	10600283	-	-	-	TRUE	-	-	-
24	10600284	-	-	-	TRUE	-	-	-
24	10600291	-	-	-	TRUE	-	-	-
24	10600294	-	-	-	TRUE	-	-	-
24	10600297	-	-	-	TRUE	TRUE	-	TRUE
24	10600507	-	-	-	TRUE	-	-	-
24	10600512	-	-	-	TRUE	TRUE	-	TRUE
24	10600521	-	-	-	TRUE	TRUE	-	TRUE
24	10600523	-	-	-	TRUE	TRUE	-	TRUE
24	10711004	-	-	-	TRUE	TRUE	-	TRUE
24	10711006	-	-	-	TRUE	TRUE	-	TRUE
24	10711007	-	-	-	TRUE	TRUE	-	TRUE
24	10711010	-	-	-	TRUE	TRUE	-	TRUE
24	10711012	-	-	-	TRUE	-	-	-
24	10711013	-	-	-	TRUE	-	-	-
24	10711015	-	-	-	TRUE	-	-	-
24	10711019	-	-	-	TRUE	-	-	-
24	10711023	-	-	-	TRUE	TRUE	-	TRUE
24	10711034	-	-	-	TRUE	-	-	-
24	10711039	-	-	-	TRUE	-	-	-
24	10711040	-	-	-	TRUE	-	-	-
24	10711042	-	-	-	TRUE	TRUE	-	TRUE
24	10711050	-	-	-	TRUE	TRUE	-	TRUE
24	10711052	-	-	-	TRUE	-	-	-
24	10711058	-	-	-	TRUE	TRUE	-	TRUE

Dataset ID	Family ID	Merged	Mendel errors	Relationship errors	CAPS-Geno	Multiplex	High bitsize	Analysis
24	10711060	-	-	-	TRUE	TRUE	-	TRUE
24	10711061	-	-	-	TRUE	-	-	-
24	10810830	-	-	-	TRUE	-	-	-
24	10810950	-	-	-	TRUE	-	-	-
24	10811010	-	-	-	TRUE	-	-	-
24	10811070	-	-	-	TRUE	TRUE	-	TRUE
24	10811150	-	-	-	TRUE	-	-	-
24	10811160	-	-	-	TRUE	-	-	-
24	10811210	-	-	-	TRUE	-	-	-
24	10811220	-	-	-	TRUE	-	-	-
24	10811240	-	-	-	TRUE	TRUE	-	TRUE
24	10811280	-	-	-	TRUE	-	-	-
24	10811320	-	-	-	TRUE	TRUE	-	TRUE
24	10811370	-	-	-	TRUE	-	-	-
24	10811420	-	-	-	TRUE	-	-	-
24	10811470	-	-	-	TRUE	-	-	-
24	10811540	-	-	-	TRUE	-	-	-
24	10811550	-	-	-	TRUE	-	-	-
24	10811560	-	-	-	TRUE	TRUE	-	TRUE
24	10811870	-	-	-	TRUE	-	-	-
24	10811930	-	-	-	TRUE	TRUE	-	TRUE
24	10811960	-	-	-	TRUE	-	-	-
24	10812040	-	-	-	TRUE	-	-	-
24	10812090	-	-	-	TRUE	-	-	-
24	10812110	-	-	-	TRUE	-	-	-
24	10812150	-	-	-	TRUE	-	-	-
24	103MS002	-	-	TRUE	-	-	-	-
24	103MS010	-	-	-	TRUE	-	-	-
24	103MS016	-	-	-	TRUE	-	-	-
24	103MS020	-	-	-	TRUE	-	-	-
24	103MS024	-	-	-	TRUE	-	-	-
24	103MS032	-	-	-	TRUE	-	-	-
24	103MS046	-	-	-	TRUE	-	-	-
24	103MS049	-	-	-	TRUE	-	-	-
24	103MS053	-	-	-	TRUE	-	-	-
24	103MS059	-	-	-	TRUE	-	-	-
24	103MS066	-	-	-	TRUE	-	-	-
24	103MS069	-	-	-	TRUE	-	-	-
24	103MS082	-	-	-	TRUE	-	-	-
24	103MS099	-	-	-	TRUE	TRUE	-	TRUE
1_AA	3030101	-	-	-	TRUE	TRUE	-	TRUE
1_AA	3030104	-	-	-	TRUE	TRUE	-	TRUE
1_AA	3030105	-	-	-	TRUE	-	-	-
1_AA	3030107	-	-	-	TRUE	TRUE	-	TRUE
1_AA	3030110	-	-	-	TRUE	-	-	-
1_AA	3030113	-	-	-	TRUE	TRUE	-	TRUE
1_AA	3030116	-	-	-	TRUE	TRUE	-	TRUE
1_AA	3030117	-	-	-	TRUE	TRUE	-	TRUE
1_AA	3030120	-	-	-	TRUE	TRUE	-	TRUE
1_AA	3030121	-	-	-	TRUE	-	-	-
1_AA	3030123	-	-	-	TRUE	TRUE	-	TRUE
1_AA	3030124	-	-	-	TRUE	TRUE	-	TRUE
1_AA	3030126	-	-	-	TRUE	TRUE	-	TRUE
1_AA	3030127	-	-	-	TRUE	-	-	-
1_AA	3030130	-	-	-	TRUE	TRUE	-	TRUE
1_AA	3030131	-	-	-	TRUE	-	-	-
1_AA	3030135	-	-	-	TRUE	TRUE	-	TRUE

Dataset ID	Family ID	Merged	Mendel errors	Relationship errors	CAPS-Geno	Multiplex	High bitsize	Analysis
1_AA	3030136	-	-	-	TRUE	TRUE	-	TRUE
1_AA	3030138	-	-	-	TRUE	TRUE	-	TRUE
1_AA	3030140	-	-	-	TRUE	TRUE	-	TRUE
1_AA	3131109	-	-	-	TRUE	TRUE	-	TRUE
1_AA	3131115	-	-	-	TRUE	-	-	-
1_AA	3131116	-	-	-	TRUE	TRUE	-	TRUE
1_AA	3131117	-	-	-	TRUE	TRUE	-	TRUE
1_AA	3131135	-	-	-	TRUE	-	-	-
1_AA	3232206	-	-	-	TRUE	TRUE	-	TRUE
1_AA	3232301	-	-	-	TRUE	-	-	-
1_AA	3232309	-	-	-	TRUE	-	-	-
1_EA	3030103	-	-	-	TRUE	-	-	-
1_EA	3030106	-	-	-	TRUE	TRUE	-	TRUE
1_EA	3030108	-	-	-	TRUE	TRUE	-	TRUE
1_EA	3030111	-	-	-	TRUE	TRUE	-	TRUE
1_EA	3030114	-	-	-	TRUE	-	-	-
1_EA	3030119	-	-	-	TRUE	-	-	-
1_EA	3030122	-	-	-	TRUE	-	-	-
1_EA	3030128	-	-	-	TRUE	TRUE	-	TRUE
1_EA	3030134	-	-	-	TRUE	TRUE	-	TRUE
1_EA	3030142	-	-	-	TRUE	TRUE	-	TRUE
1_EA	3131102	-	-	-	TRUE	-	-	-
1_EA	3131104	-	-	-	TRUE	TRUE	-	TRUE
1_EA	3131107	-	-	-	TRUE	-	-	-
1_EA	3131110	-	-	-	TRUE	-	-	-
1_EA	3131113	-	-	-	TRUE	TRUE	-	TRUE
1_EA	3131119	-	-	-	TRUE	TRUE	-	TRUE
1_EA	3131120	-	-	-	TRUE	-	-	-
1_EA	3131126	-	-	-	TRUE	-	-	-
1_EA	3131137	-	-	-	TRUE	TRUE	-	TRUE
1_EA	3131148	-	-	-	TRUE	-	-	-
1_EA	3131152	-	-	-	TRUE	TRUE	-	TRUE
1_EA	3131154	-	-	-	TRUE	TRUE	-	TRUE
1_EA	3131155	-	-	-	TRUE	-	-	-
1_EA	3232101	-	-	-	TRUE	TRUE	-	TRUE
1_EA	3232102	-	-	-	TRUE	-	-	-
1_EA	3232103	-	-	-	TRUE	TRUE	-	TRUE
1_EA	3232104	-	-	-	TRUE	-	-	-
1_EA	3232108	-	-	-	TRUE	-	-	-
1_EA	3232201	-	-	-	TRUE	-	-	-
1_EA	3232204	-	-	-	TRUE	TRUE	-	TRUE
1_EA	3232205	-	-	-	TRUE	-	-	-
1_EA	3232208	-	-	-	TRUE	TRUE	-	TRUE
1_EA	3232209	-	-	-	TRUE	-	-	-
1_EA	3232210	-	-	-	TRUE	TRUE	-	TRUE
1_EA	3232211	-	-	-	TRUE	-	-	-
1_EA	3232212	-	-	-	TRUE	TRUE	-	TRUE
1_EA	3232213	-	-	TRUE	-	-	-	-
1_EA	3232218	-	-	-	TRUE	TRUE	-	TRUE
1_EA	3232219	-	-	-	TRUE	TRUE	-	TRUE
1_EA	3232304	-	-	-	TRUE	-	-	-
1_EA	3232306	-	-	-	TRUE	-	-	-
1_EA	3232307	-	-	-	TRUE	-	-	-
1_EA	3232310	-	-	-	TRUE	-	-	-
1_EA	3232311	-	-	-	TRUE	-	-	-
1_EA	3232312	-	-	-	TRUE	TRUE	-	TRUE
1_EA	3232313	-	-	-	TRUE	-	-	-

Dataset ID	Family ID	Merged	Mendel errors	Relationship errors	CAPS-Geno	Multiplex	High bitsize	Analysis
11_AA	421002	-	-	-	TRUE	TRUE	-	TRUE
11_AA	421003	-	-	-	TRUE	TRUE	-	TRUE
11_AA	421004	-	-	-	TRUE	TRUE	-	TRUE
11_AA	421008	-	-	-	TRUE	TRUE	-	TRUE
11_AA	421009	-	-	-	TRUE	TRUE	-	TRUE
11_AA	421013	-	-	-	TRUE	-	-	-
11_AA	421014	-	-	-	TRUE	TRUE	-	TRUE
11_AA	421021	-	-	-	TRUE	-	-	-
11_AA	421022	-	-	-	TRUE	TRUE	-	TRUE
11_AA	421028	-	-	-	TRUE	TRUE	-	TRUE
11_AA	421029	-	-	-	TRUE	TRUE	-	TRUE
11_AA	421031	-	-	-	TRUE	-	-	-
11_AA	421033	-	-	-	TRUE	TRUE	-	TRUE
11_AA	421038	-	-	-	TRUE	TRUE	-	TRUE
11_AA	421042	-	-	-	TRUE	TRUE	-	TRUE
11_AA	421045	-	-	-	TRUE	-	-	-
11_AA	421047	-	-	-	TRUE	-	-	-
11_AA	421049	-	-	-	TRUE	-	-	-
11_AA	421051	-	-	-	TRUE	TRUE	-	TRUE
11_AA	421054	-	-	-	TRUE	-	-	-
11_AA	421057	-	-	-	TRUE	-	-	-
11_AA	421058	-	-	-	TRUE	-	-	-
11_AA	421060	-	-	-	TRUE	TRUE	-	TRUE
11_AA	421062	-	-	-	TRUE	-	-	-
11_AA	421064	-	-	-	TRUE	-	-	-
11_AA	421065	-	-	-	TRUE	TRUE	-	TRUE
11_AA	421068	-	-	-	TRUE	-	-	-
11_AA	421071	-	-	-	TRUE	-	-	-
11_AA	421078	-	-	-	TRUE	-	-	-
11_AA	431013	-	-	-	TRUE	-	-	-
11_AA	431015	-	-	-	TRUE	-	-	-
11_AA	431018	-	-	-	TRUE	TRUE	-	TRUE
11_AA	431028	-	-	-	TRUE	TRUE	-	TRUE
11_AA	431032	-	-	-	TRUE	-	-	-
11_AA	431037	-	-	-	TRUE	-	-	-
11_AA	431038	-	-	-	TRUE	-	-	-
11_AA	431062	-	-	-	TRUE	TRUE	-	TRUE
11_AA	431072	-	-	-	TRUE	-	-	-
11_AA	441003	-	-	-	TRUE	TRUE	-	TRUE
11_AA	441004	-	-	-	TRUE	TRUE	-	TRUE
11_AA	441009	-	-	-	TRUE	TRUE	-	TRUE
11_AA	441011	-	-	-	TRUE	-	-	-
11_AA	441019	-	-	-	TRUE	TRUE	-	TRUE
11_AA	441020	-	-	-	TRUE	TRUE	-	TRUE
11_AA	441022	-	-	-	TRUE	TRUE	-	TRUE
11_AA	441028	-	-	-	TRUE	TRUE	-	TRUE
11_AA	441030	-	-	-	TRUE	-	-	-
11_AA	441033	-	-	-	TRUE	TRUE	-	TRUE
11_AA	441034	-	-	-	TRUE	TRUE	-	TRUE
11_AA	441037	-	-	-	TRUE	-	-	-
11_AA	441038	-	-	-	TRUE	-	-	-
11_AA	441040	-	-	-	TRUE	-	-	-
11_AA	441041	-	-	-	TRUE	TRUE	-	TRUE
11_AA	441043	-	-	-	TRUE	TRUE	-	TRUE
11_AA	441046	-	-	-	TRUE	TRUE	-	TRUE
11_AA	441047	-	-	-	TRUE	TRUE	-	TRUE
11_AA	441049	-	-	-	TRUE	TRUE	-	TRUE

Dataset ID	Family ID	Merged	Mendel errors	Relationship errors	CAPS-Geno	Multiplex	High bitsize	Analysis
11_AA	441051	-	-	-	TRUE	-	-	-
11_AA	441053	-	-	-	TRUE	TRUE	-	TRUE
11_AA	441054	-	-	-	TRUE	TRUE	-	TRUE
11_AA	441056	-	-	-	TRUE	-	-	-
11_AA	441057	-	-	-	TRUE	TRUE	-	TRUE
11_AA	441060	-	-	-	TRUE	TRUE	-	TRUE
11_AA	441062	-	-	-	TRUE	TRUE	-	TRUE
11_AA	441064	-	-	-	TRUE	TRUE	-	TRUE
11_AA	441066	-	-	-	TRUE	TRUE	-	TRUE
11_AA	441067	-	-	-	TRUE	-	-	-
11_AA	441070	-	-	-	TRUE	TRUE	-	TRUE
11_AA	441071	-	-	-	TRUE	TRUE	-	TRUE
11_AA	441072	-	-	-	TRUE	-	-	-
11_AA	441074	-	-	-	TRUE	-	-	-
11_AA	451006	-	-	-	TRUE	-	-	-
11_AA	451013	-	-	-	TRUE	TRUE	-	TRUE
11_AA	451015	-	-	-	TRUE	-	-	-
11_AA	451020	-	-	-	TRUE	TRUE	-	TRUE
11_AA	451029	-	-	-	TRUE	TRUE	-	TRUE
11_AA	451032	-	-	-	TRUE	TRUE	-	TRUE
11_AA	451034	-	-	-	TRUE	TRUE	-	TRUE
11_AA	451036	-	-	-	TRUE	TRUE	-	TRUE
11_AA	461019	-	-	-	TRUE	TRUE	-	TRUE
11_AA	471004	-	-	-	TRUE	TRUE	-	TRUE
11_AA	471005	-	-	-	TRUE	TRUE	-	TRUE
11_AA	471007	-	-	-	TRUE	-	-	-
11_AA	471018	-	-	-	TRUE	-	-	-
11_AA	471027	-	-	-	TRUE	-	-	-
11_AA	471029	-	-	-	TRUE	TRUE	-	TRUE
11_AA	471030	-	-	-	TRUE	-	-	-
11_AA	471035	-	-	-	TRUE	-	-	-
11_AA	471039	-	-	-	TRUE	-	-	-
11_AA	471043	-	-	-	TRUE	TRUE	-	TRUE
11_AA	471047	-	-	-	TRUE	TRUE	-	TRUE
11_AA	471049	-	-	-	TRUE	TRUE	-	TRUE
11_AA	471051	-	-	-	TRUE	TRUE	-	TRUE
11_AA	471061	-	-	-	TRUE	-	-	-
11_AA	471063	-	-	-	TRUE	-	-	-
11_AA	471086	-	-	-	TRUE	TRUE	-	TRUE
11_AA	471087	-	-	-	TRUE	-	-	-
11_AA	471088	-	-	-	TRUE	TRUE	-	TRUE
11_AA	471089	-	-	-	TRUE	-	-	-
11_AA	477002	-	-	-	TRUE	-	-	-
11_AA	481002	-	-	-	TRUE	TRUE	-	TRUE
11_AA	481011	-	-	-	TRUE	-	-	-
11_AA	481015	-	-	-	TRUE	TRUE	-	TRUE
11_AA	481018	-	-	-	TRUE	-	-	-
11_AA	481024	-	-	-	TRUE	TRUE	-	TRUE
11_AA	481025	-	-	-	TRUE	TRUE	-	TRUE
11_AA	481028	-	-	-	TRUE	-	-	-
11_AA	481030	-	-	-	TRUE	-	-	-
11_AA	481032	-	-	-	TRUE	TRUE	-	TRUE
11_AA	481033	-	-	-	TRUE	-	-	-
11_AA	489001	-	-	-	TRUE	TRUE	-	TRUE
11_AA	489002	-	-	-	TRUE	TRUE	-	TRUE
11_AA	489003	-	-	-	TRUE	-	-	-
11_AA	489004	-	-	-	TRUE	TRUE	-	TRUE

Dataset ID	Family ID	Merged	Mendel errors	Relationship errors	CAPS-Geno	Multiplex	High bitsize	Analysis
11_AA	489007	-	-	-	TRUE	TRUE	-	TRUE
11_AA	489008	-	-	-	TRUE	-	-	-
11_AA	489009	-	-	-	TRUE	TRUE	-	TRUE
11_AA	489012	-	-	-	TRUE	TRUE	-	TRUE
11_AA	489013	-	-	-	TRUE	TRUE	-	TRUE
11_AA	489015	-	-	-	TRUE	-	-	-
11_AA	489016	-	-	-	TRUE	-	-	-
11_AA	489018	-	-	-	TRUE	TRUE	-	TRUE
11_AA	489019	-	-	-	TRUE	-	-	-
11_AA	489022	-	-	-	TRUE	-	-	-
11_AA	489023	-	-	-	TRUE	-	-	-
11_AA	489024	-	-	-	TRUE	TRUE	-	TRUE
11_AA	489025	-	-	-	TRUE	TRUE	-	TRUE
11_AA	489026	-	-	-	TRUE	TRUE	-	TRUE
11_AA	491001	-	-	-	TRUE	-	-	-
11_AA	491002	-	-	-	TRUE	TRUE	-	TRUE
11_AA	491004	-	-	-	TRUE	TRUE	-	TRUE
11_AA	491005	-	-	-	TRUE	-	-	-
11_AA	491007	-	-	-	TRUE	TRUE	-	TRUE
11_AA	491010	-	-	-	TRUE	TRUE	-	TRUE
11_AA	491011	-	-	-	TRUE	TRUE	-	TRUE
11_AA	491014	-	-	-	TRUE	TRUE	-	TRUE
11_AA	491015	-	-	-	TRUE	TRUE	-	TRUE
11_AA	491017	-	-	-	TRUE	TRUE	-	TRUE
11_AA	491018	-	-	-	TRUE	TRUE	-	TRUE
11_AA	491024	-	-	-	TRUE	TRUE	-	TRUE
11_AA	491025	-	-	-	TRUE	-	-	-
11_AA	491026	-	-	-	TRUE	TRUE	-	TRUE
11_AA	491028	-	-	-	TRUE	-	-	-
11_AA	491032	-	-	-	TRUE	-	-	-
11_AA	491033	-	-	-	TRUE	TRUE	-	TRUE
11_AA	491039	-	-	-	TRUE	TRUE	-	TRUE
11_EA	410001	-	-	-	TRUE	-	-	-
11_EA	410002	-	-	-	TRUE	TRUE	-	TRUE
11_EA	410003	-	-	-	TRUE	TRUE	-	TRUE
11_EA	410004	-	-	-	TRUE	-	-	-
11_EA	410005	-	-	-	TRUE	TRUE	-	TRUE
11_EA	410007	-	-	-	TRUE	TRUE	-	TRUE
11_EA	410008	-	-	-	TRUE	TRUE	-	TRUE
11_EA	410010	-	-	-	TRUE	-	-	-
11_EA	410011	-	-	-	TRUE	TRUE	-	TRUE
11_EA	410012	-	-	-	TRUE	TRUE	-	TRUE
11_EA	410013	-	-	-	TRUE	-	-	-
11_EA	410014	-	-	-	TRUE	-	-	-
11_EA	410015	-	-	-	TRUE	TRUE	-	TRUE
11_EA	410016	-	-	-	TRUE	-	-	-
11_EA	410017	-	-	-	TRUE	TRUE	-	TRUE
11_EA	410018	-	-	-	TRUE	-	-	-
11_EA	410019	-	-	-	TRUE	TRUE	-	TRUE
11_EA	410020	-	-	-	TRUE	TRUE	-	TRUE
11_EA	410021	-	-	-	TRUE	-	-	-
11_EA	410024	-	-	-	TRUE	-	-	-
11_EA	410025	-	-	-	TRUE	TRUE	-	TRUE
11_EA	410026	-	-	-	TRUE	-	-	-
11_EA	410028	-	-	-	TRUE	TRUE	-	TRUE
11_EA	410029	-	-	-	TRUE	TRUE	-	TRUE
11_EA	410030	-	-	-	TRUE	TRUE	-	TRUE

Dataset ID	Family ID	Merged	Mendel errors	Relationship errors	CAPS-Geno	Multiplex	High bitsize	Analysis
11_EA	410033	-	-	-	TRUE	-	-	-
11_EA	410034	-	-	-	TRUE	TRUE	-	TRUE
11_EA	410035	-	-	-	TRUE	-	-	-
11_EA	410036	-	-	-	TRUE	-	-	-
11_EA	410037	-	-	-	TRUE	TRUE	-	TRUE
11_EA	410038	-	-	-	TRUE	TRUE	-	TRUE
11_EA	410039	-	-	-	TRUE	TRUE	-	TRUE
11_EA	410040	-	-	-	TRUE	TRUE	-	TRUE
11_EA	410041	-	-	-	TRUE	TRUE	-	TRUE
11_EA	410042	-	-	-	TRUE	-	-	-
11_EA	410044	-	-	-	TRUE	-	-	-
11_EA	410045	-	-	-	TRUE	-	-	-
11_EA	410046	-	-	-	TRUE	-	-	-
11_EA	410047	-	-	-	TRUE	-	-	-
11_EA	410048	-	-	-	TRUE	TRUE	-	TRUE
11_EA	410049	-	-	-	TRUE	TRUE	-	TRUE
11_EA	410050	-	-	-	TRUE	-	-	-
11_EA	410051	-	-	-	TRUE	-	-	-
11_EA	410052	-	-	-	TRUE	-	-	-
11_EA	410053	-	-	-	TRUE	TRUE	-	TRUE
11_EA	410054	-	-	-	TRUE	TRUE	-	TRUE
11_EA	410056	-	-	-	TRUE	TRUE	-	TRUE
11_EA	410057	-	-	-	TRUE	TRUE	-	TRUE
11_EA	410058	-	-	-	TRUE	TRUE	-	TRUE
11_EA	410060	-	-	-	TRUE	TRUE	-	TRUE
11_EA	410061	-	-	-	TRUE	TRUE	-	TRUE
11_EA	410062	-	-	-	TRUE	-	-	-
11_EA	410063	-	-	-	TRUE	-	-	-
11_EA	410064	-	-	-	TRUE	-	-	-
11_EA	410065	-	-	-	TRUE	-	-	-
11_EA	410066	-	-	-	TRUE	TRUE	-	TRUE
11_EA	410067	-	-	-	TRUE	TRUE	-	TRUE
11_EA	410071	-	-	-	TRUE	TRUE	-	TRUE
11_EA	410072	-	-	-	TRUE	TRUE	-	TRUE
11_EA	410075	-	-	-	TRUE	TRUE	-	TRUE
11_EA	410076	-	-	-	TRUE	TRUE	-	TRUE
11_EA	410078	-	-	-	TRUE	TRUE	-	TRUE
11_EA	410079	-	-	-	TRUE	TRUE	-	TRUE
11_EA	410080	-	-	-	TRUE	-	-	-
11_EA	410082	-	-	-	TRUE	-	-	-
11_EA	410083	-	-	-	TRUE	TRUE	-	TRUE
11_EA	410084	-	-	-	TRUE	TRUE	-	TRUE
11_EA	410085	-	-	-	TRUE	TRUE	-	TRUE
11_EA	410086	-	-	-	TRUE	-	-	-
11_EA	410088	-	-	-	TRUE	-	-	-
11_EA	410090	-	-	-	TRUE	-	-	-
11_EA	410091	-	-	-	TRUE	-	-	-
11_EA	410092	-	-	-	TRUE	TRUE	-	TRUE
11_EA	410093	-	-	-	TRUE	-	-	-
11_EA	410094	-	-	-	TRUE	-	-	-
11_EA	410096	-	-	-	TRUE	TRUE	-	TRUE
11_EA	421001	-	-	-	TRUE	TRUE	-	TRUE
11_EA	421006	-	-	-	TRUE	TRUE	-	TRUE
11_EA	421007	-	-	-	TRUE	TRUE	-	TRUE
11_EA	421010	-	-	-	TRUE	TRUE	-	TRUE
11_EA	421011	-	-	-	TRUE	TRUE	-	TRUE
11_EA	421016	-	-	-	TRUE	TRUE	-	TRUE

Dataset ID	Family ID	Merged	Mendel errors	Relationship errors	CAPS-Geno	Multiplex	High bitsize	Analysis
11_EA	421024	-	-	-	TRUE	TRUE	-	TRUE
11_EA	421025	-	-	-	TRUE	TRUE	-	TRUE
11_EA	421026	-	-	-	TRUE	TRUE	-	TRUE
11_EA	421027	-	-	-	TRUE	-	-	-
11_EA	421030	-	-	-	TRUE	TRUE	-	TRUE
11_EA	421032	-	-	-	TRUE	TRUE	-	TRUE
11_EA	421034	-	-	-	TRUE	TRUE	-	TRUE
11_EA	421035	-	-	-	TRUE	TRUE	-	TRUE
11_EA	421036	-	-	-	TRUE	TRUE	-	TRUE
11_EA	421037	-	-	-	TRUE	-	-	-
11_EA	421039	-	-	-	TRUE	TRUE	-	TRUE
11_EA	421040	-	-	-	TRUE	TRUE	-	TRUE
11_EA	421041	-	-	-	TRUE	TRUE	-	TRUE
11_EA	421043	-	-	-	TRUE	TRUE	-	TRUE
11_EA	421044	-	-	-	TRUE	TRUE	-	TRUE
11_EA	421048	-	-	-	TRUE	TRUE	-	TRUE
11_EA	421053	-	-	-	TRUE	TRUE	-	TRUE
11_EA	421063	-	-	-	TRUE	TRUE	-	TRUE
11_EA	421072	-	-	-	TRUE	-	-	-
11_EA	421074	-	-	-	TRUE	-	-	-
11_EA	421077	-	-	-	TRUE	-	-	-
11_EA	431001	-	-	-	TRUE	TRUE	-	TRUE
11_EA	431002	-	-	-	TRUE	TRUE	-	TRUE
11_EA	431003	-	-	-	TRUE	TRUE	-	TRUE
11_EA	431004	-	-	-	TRUE	TRUE	-	TRUE
11_EA	431005	-	-	-	TRUE	TRUE	-	TRUE
11_EA	431006	-	-	-	TRUE	TRUE	-	TRUE
11_EA	431007	-	-	-	TRUE	-	-	-
11_EA	431008	-	-	-	TRUE	TRUE	-	TRUE
11_EA	431012	-	-	-	TRUE	-	-	-
11_EA	431014	-	-	-	TRUE	-	-	-
11_EA	431016	-	-	-	TRUE	TRUE	-	TRUE
11_EA	431019	-	-	-	TRUE	-	-	-
11_EA	431023	-	-	-	TRUE	-	-	-
11_EA	431024	-	-	-	TRUE	-	-	-
11_EA	431025	-	-	-	TRUE	-	-	-
11_EA	431026	-	-	-	TRUE	TRUE	-	TRUE
11_EA	431027	-	-	-	TRUE	TRUE	-	TRUE
11_EA	431029	-	-	-	TRUE	TRUE	-	TRUE
11_EA	431031	-	-	-	TRUE	TRUE	-	TRUE
11_EA	431034	-	-	-	TRUE	-	-	-
11_EA	431036	-	-	-	TRUE	TRUE	-	TRUE
11_EA	431040	-	-	-	TRUE	TRUE	-	TRUE
11_EA	431041	-	-	-	TRUE	-	-	-
11_EA	431044	-	-	-	TRUE	TRUE	-	TRUE
11_EA	431046	-	-	-	TRUE	-	-	-
11_EA	431050	-	-	-	TRUE	TRUE	-	TRUE
11_EA	431051	-	-	-	TRUE	-	-	-
11_EA	431052	-	-	-	TRUE	TRUE	-	TRUE
11_EA	431054	-	-	-	TRUE	TRUE	-	TRUE
11_EA	431056	-	-	-	TRUE	TRUE	-	TRUE
11_EA	431058	-	-	-	TRUE	-	-	-
11_EA	431060	-	-	-	TRUE	-	-	-
11_EA	431061	-	-	-	TRUE	-	-	-
11_EA	431064	-	-	-	TRUE	-	-	-
11_EA	431065	-	-	-	TRUE	TRUE	-	TRUE
11_EA	431066	-	-	-	TRUE	TRUE	-	TRUE

Dataset ID	Family ID	Merged	Mendel errors	Relationship errors	CAPS-Geno	Multiplex	High bitsize	Analysis
11_EA	431068	-	-	-	TRUE	-	-	-
11_EA	431070	-	-	-	TRUE	TRUE	-	TRUE
11_EA	431071	-	-	-	TRUE	-	-	-
11_EA	431073	-	-	-	TRUE	-	-	-
11_EA	431074	-	-	-	TRUE	TRUE	-	TRUE
11_EA	441001	-	-	-	TRUE	TRUE	-	TRUE
11_EA	441018	-	-	-	TRUE	-	-	-
11_EA	441025	-	-	-	TRUE	-	-	-
11_EA	441026	-	-	-	TRUE	-	-	-
11_EA	441027	-	-	-	TRUE	TRUE	-	TRUE
11_EA	441031	-	-	-	TRUE	-	-	-
11_EA	441044	-	-	-	TRUE	TRUE	-	TRUE
11_EA	441045	-	-	-	TRUE	-	-	-
11_EA	441048	-	-	-	TRUE	TRUE	-	TRUE
11_EA	441052	-	-	-	TRUE	-	-	-
11_EA	441069	-	-	-	TRUE	-	-	-
11_EA	441075	-	-	-	TRUE	TRUE	-	TRUE
11_EA	451001	-	-	-	TRUE	-	-	-
11_EA	451002	-	-	-	TRUE	-	-	-
11_EA	451004	-	-	-	TRUE	TRUE	-	TRUE
11_EA	451007	-	-	-	TRUE	TRUE	-	TRUE
11_EA	451009	-	-	-	TRUE	TRUE	-	TRUE
11_EA	451010	-	-	-	TRUE	TRUE	-	TRUE
11_EA	451011	-	-	-	TRUE	TRUE	-	TRUE
11_EA	451012	-	-	-	TRUE	TRUE	-	TRUE
11_EA	451030	-	-	-	TRUE	TRUE	-	TRUE
11_EA	451033	-	-	-	TRUE	TRUE	-	TRUE
11_EA	451037	-	-	-	TRUE	TRUE	-	TRUE
11_EA	451039	-	-	-	TRUE	TRUE	-	TRUE
11_EA	451040	-	-	-	TRUE	TRUE	-	TRUE
11_EA	451041	-	-	-	TRUE	TRUE	-	TRUE
11_EA	451044	-	-	-	TRUE	TRUE	-	TRUE
11_EA	451046	-	-	-	TRUE	TRUE	-	TRUE
11_EA	461002	-	-	-	TRUE	-	-	-
11_EA	461003	-	-	-	TRUE	TRUE	-	TRUE
11_EA	461004	-	-	-	TRUE	TRUE	-	TRUE
11_EA	461005	-	-	-	TRUE	-	-	-
11_EA	461007	-	-	-	TRUE	TRUE	-	TRUE
11_EA	461009	-	-	-	TRUE	TRUE	-	TRUE
11_EA	461010	-	-	-	TRUE	-	-	-
11_EA	461011	-	-	-	TRUE	TRUE	-	TRUE
11_EA	461013	-	-	-	TRUE	TRUE	-	TRUE
11_EA	461015	-	-	-	TRUE	-	-	-
11_EA	461017	-	-	-	TRUE	TRUE	-	TRUE
11_EA	461021	-	-	-	TRUE	-	-	-
11_EA	461022	-	-	-	TRUE	TRUE	-	TRUE
11_EA	461025	-	-	-	TRUE	TRUE	-	TRUE
11_EA	461028	-	-	-	TRUE	-	-	-
11_EA	461030	-	-	-	TRUE	TRUE	-	TRUE
11_EA	461033	-	-	-	TRUE	-	-	-
11_EA	461035	-	-	-	TRUE	TRUE	-	TRUE
11_EA	461036	-	-	-	TRUE	TRUE	-	TRUE
11_EA	461038	-	-	-	TRUE	TRUE	-	TRUE
11_EA	461039	-	-	-	TRUE	TRUE	-	TRUE
11_EA	461040	-	-	-	TRUE	TRUE	-	TRUE
11_EA	461042	-	-	-	TRUE	TRUE	-	TRUE
11_EA	461043	-	-	-	TRUE	-	-	-

Dataset ID	Family ID	Merged	Mendel errors	Relationship errors	CAPS-Geno	Multiplex	High bitsize	Analysis
11_EA	461044	-	-	-	TRUE	TRUE	-	TRUE
11_EA	461045	-	-	-	TRUE	TRUE	-	TRUE
11_EA	461050	-	-	-	TRUE	TRUE	-	TRUE
11_EA	461051	-	-	-	TRUE	TRUE	-	TRUE
11_EA	461052	-	-	-	TRUE	TRUE	-	TRUE
11_EA	471003	-	-	-	TRUE	-	-	-
11_EA	471008	-	-	-	TRUE	TRUE	-	TRUE
11_EA	471009	-	-	-	TRUE	TRUE	-	TRUE
11_EA	471010	-	-	-	TRUE	TRUE	-	TRUE
11_EA	471023	-	-	-	TRUE	-	-	-
11_EA	471024	-	-	-	TRUE	TRUE	-	TRUE
11_EA	471025	-	-	-	TRUE	TRUE	-	TRUE
11_EA	471033	-	-	-	TRUE	TRUE	-	TRUE
11_EA	471040	-	-	-	TRUE	-	-	-
11_EA	471044	-	-	-	TRUE	-	-	-
11_EA	471050	-	-	-	TRUE	-	-	-
11_EA	471053	-	-	-	TRUE	-	-	-
11_EA	471056	-	-	-	TRUE	TRUE	-	TRUE
11_EA	471059	-	-	-	TRUE	TRUE	-	TRUE
11_EA	471062	-	-	-	TRUE	TRUE	-	TRUE
11_EA	471064	-	-	-	TRUE	-	-	-
11_EA	471066	-	-	-	TRUE	TRUE	-	TRUE
11_EA	471067	-	-	-	TRUE	TRUE	-	TRUE
11_EA	471070	-	-	-	TRUE	TRUE	-	TRUE
11_EA	471085	-	-	-	TRUE	TRUE	-	TRUE
11_EA	471090	-	-	-	TRUE	TRUE	-	TRUE
11_EA	471091	-	-	-	TRUE	TRUE	-	TRUE
11_EA	471092	-	-	-	TRUE	TRUE	-	TRUE
11_EA	471093	-	-	-	TRUE	TRUE	-	TRUE
11_EA	481001	-	-	-	TRUE	TRUE	-	TRUE
11_EA	481003	-	-	-	TRUE	TRUE	-	TRUE
11_EA	481004	-	-	-	TRUE	TRUE	-	TRUE
11_EA	481007	-	-	-	TRUE	TRUE	-	TRUE
11_EA	481008	-	-	-	TRUE	TRUE	-	TRUE
11_EA	481010	-	-	-	TRUE	TRUE	-	TRUE
11_EA	481012	-	-	-	TRUE	TRUE	-	TRUE
11_EA	481014	-	-	-	TRUE	TRUE	-	TRUE
11_EA	481016	-	-	-	TRUE	TRUE	-	TRUE
11_EA	481017	-	-	-	TRUE	-	-	-
11_EA	481021	-	-	-	TRUE	-	-	-
11_EA	481026	-	-	-	TRUE	TRUE	-	TRUE
11_EA	481027	-	-	-	TRUE	-	-	-
11_EA	481031	-	-	-	TRUE	TRUE	-	TRUE
11_EA	481034	-	-	-	TRUE	TRUE	-	TRUE
11_EA	481035	-	-	-	TRUE	TRUE	-	TRUE
11_EA	481036	-	-	-	TRUE	-	-	-
11_EA	481037	-	-	-	TRUE	-	-	-
11_EA	481038	-	-	-	TRUE	-	-	-
11_EA	481039	-	-	-	TRUE	-	-	-
11_EA	481041	-	-	-	TRUE	-	-	-
11_EA	489010	-	-	-	TRUE	-	-	-
11_EA	491003	-	-	-	TRUE	-	-	-
11_EA	491006	-	-	-	TRUE	TRUE	-	TRUE
11_EA	491008	-	-	-	TRUE	-	-	-
11_EA	491009	-	-	-	TRUE	TRUE	-	TRUE
11_EA	491012	-	-	-	TRUE	TRUE	-	TRUE
11_EA	491016	-	-	-	TRUE	-	-	-

Dataset ID	Family ID	Merged	Mendel errors	Relationship errors	CAPS-Geno	Multiplex	High bitsize	Analysis
11_EA	491019	-	-	-	TRUE	-	-	-
11_EA	491020	-	-	-	TRUE	-	-	-
11_EA	491021	-	-	-	TRUE	-	-	-
11_EA	491023	-	-	-	TRUE	-	-	-
11_EA	491027	-	-	-	TRUE	TRUE	-	TRUE
11_EA	491029	-	-	-	TRUE	-	-	-
11_EA	491030	-	-	-	TRUE	TRUE	-	TRUE
11_EA	491031	-	-	-	TRUE	-	-	-
11_EA	491034	-	-	-	TRUE	-	-	-
11_EA	491035	-	-	-	TRUE	-	-	-
11_EA	491036	-	-	-	TRUE	TRUE	-	TRUE
11_EA	491037	-	-	-	TRUE	TRUE	-	TRUE
11_EA	491038	-	-	-	TRUE	TRUE	-	TRUE
